# Allosteric switching of Ca^2+^ permeability in the lysosomal ion channel TPC2 underpins biased agonist activation

**DOI:** 10.1038/s44318-026-00882-1

**Published:** 2026-08-07

**Authors:** Qianru Mu, Ryo Saito, Franceska Ozola, Dawid Jaślan, Pak Nin Chu, Afroditi-Maria Zaki, Marcos Rubio-Alarcon, Marco Keller, Yu Yuan, Alan Greig, Virginia Silio, Franz Bracher, Philip C Biggin, Gabriela C Brailoiu, Eugen Brailoiu, Jonathan S Marchant, Christian Grimm, Eric J Lambie, Taufiq Rahman, Sandip Patel

**Affiliations:** 1 Department of Cell and Developmental Biology, University College London, London, UK; 2 Department of Dermatology, Graduate School of Biomedical and Health Sciences, Hiroshima University, Hiroshima, Japan; 3 Walther Straub Institute of Pharmacology and Toxicology, Faculty of Medicine, Ludwig-Maximilian University, Munich, Germany; 4 Structural Bioinformatics and Computational Biochemistry, Department of Biochemistry, University of Oxford, Oxford, UK; 5 Department of Pharmacology, University of Cambridge, Cambridge, UK; 6 Department of Pharmacy-Center for Drug Research, Ludwig-Maximilian University, Munich, Germany; 7 Department of Pharmaceutical Sciences, Jefferson College of Pharmacy, Thomas Jefferson University, Philadelphia, PA USA; 8 Department of Neural Sciences and Center for Substance Abuse Research, Lewis Katz School of Medicine, Temple University, Philadelphia, PA USA; 9 Department of Cell Biology, Neurobiology & Anatomy, Medical College of Wisconsin, Milwaukee, WI USA; 10 Immunology, Infection and Pandemic Research IIP, Fraunhofer Institute for Translational Medicine and Pharmacology ITMP, Munich/Frankfurt, Germany; 11 Department of Pharmacology, University of Oxford, Oxford, UK

**Keywords:** Organelles, Structural Biology

## Abstract

Ion channels possess selectivity filters that are hardwired to ensure the selective passage of ions. Lysosomal two-pore channels are unusual as they are able to switch their cation selectivity in an agonist-specific manner, allowing differential control of organellar activity. TPC2 is permeable to Ca^2+^ when activated by the calcium-mobilizing messenger NAADP, but largely Na^+^-selective when activated by the signaling lipid PI(3,5)P_2_. Co-stimulation increases Ca^2+^ but not Na^+^ permeability; however, the molecular basis for these specificity switches is not well understood. Here we show that mutation of TPC2 residues within the distal cytosolic linker, which connects the first voltage-sensing-like domain to the pore, rendered TPC2 largely unable to discriminate its agonists and highly calcium-permeable, even in the presence of PI(3,5)P_2_. This mutation induced a co-activated-like state by disrupting a network of residues that connects the linker to the activation gate. Such deregulated agonist action increased lysosomal Ca^2+^ flux and compromised locomotion and viability when expressed in *C. elegans*. A proximal disease-linked mutation perturbed agonist action in a similar way both in vitro and in vivo. Biased signaling through TPC2 thus proceeds through molecular determinants that are remote from the selectivity filter, affecting Ca^2+^ permeability, endo-lysosomal integrity and disease.

## Synopsis

The selectivity of lysosomal TPC2 ion channels for either Ca²⁺ or Na⁺ depends on which agonists activate the channel. This article shows that this plasticity is governed by a distal allosteric network that allows TPC2 to discriminate between agonists and generate specific lysosomal ion fluxes.

- Ion selectivity of TPC2 can be controlled by residues outside the canonical selectivity filter.
- Gain-of-function mutations reduce discrimination between NAADP- and PI(3,5)P₂-dependent signalling modes.
- Mutant channels exhibit enhanced Ca²⁺ permeability and adopt a co-activated-like state.

## Abstract (web summary)

A distal allosteric network controls agonist-specific ion selectivity.

## Graphical abstract

**Figure:**
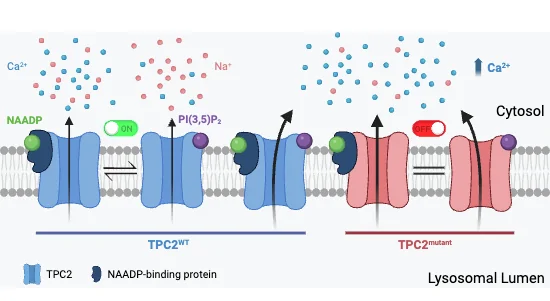

**Figure:**
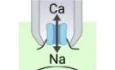

## Introduction

The movement of ions across biological membranes is fundamental to life. Ion channels permit this by forming pores that are selective for a single ion or a specific set of ions. Such selectivity is thought to be a defining, fixed feature of a given ion channel. In the large voltage-gated ion channel family, which includes the organellar two-pore channels (TPCs) (Patel, 2015), ion selectivity is achieved by the selectivity filter situated between two trans-membrane regions (S5-S6) that form the pore domain to which peripheral voltage sensing domains comprising four additional trans-membrane regions (S1–S4) are often attached (Catterall, 2023). These key modules are joined via the S4-S5 linker which together with a bundle of S6 helices that form the activation gate face the cytosol with the selectivity filter on the opposite, extracellular/luminal side of the membrane. The activation gate opens on demand allowing ion flow across the membrane through the selectivity filter. Mutations in the selectivity filter as well as the linker underpin disease underscoring their pathophysiological importance (Catterall, 2023).

TPCs hold a key position in the evolution of voltage-gated ion channels reflected by their dimeric structure comprising two-repeats of 6 trans-membrane regions (Guo et al, 2016; Patel, 2015; Rahman et al, 2014). This organization is intermediate between tetrameric family members such as K^+^ channels comprising a single shaker-like repeat and monomeric four-repeat family members exemplified by Na^+^ channels. And consistent with an evolutionary trajectory comprising two rounds of intragenic duplication of a primordial one repeat ancestor (Hille, 1984; Rahman et al, 2014). TPCs are targeted to acidic organelles such as lysosomes via their N-termini (Brailoiu et al, 2010; Larisch et al, 2012). In mammals, they regulate numerous aspects of membrane trafficking including transit of internalized cell surface receptors (Grimm et al, 2014), membrane contact site formation (Kilpatrick et al, 2017) and organelle morphology (Freeman et al, 2020; Lin-Moshier et al, 2014). Natural variants in the human TPC2 gene control pigmentation and mutations are linked to pigmentation disorders (Sulem et al, 2008; Wang et al, 2023). TPCs are emerging as drug targets in a number of diseases including neurodegeneration (Gregori et al, 2025; Hockey et al, 2015), cardiac dysfunction (Davidson et al, 2015), viral infection (Sakurai et al, 2015) and lysosomal storage disorders (Patel and Kilpatrick, 2018; Scotto Rosato et al, 2022).

Recent work has revealed the ability of TPCs to alter their ionic permeability in an agonist-selective manner (Gerndt et al, 2020a; Yuan et al, 2022). This highly unusual behavior has quelled debate regarding fundamental attributes of TPCs namely whether they are Ca^2+^ or Na^+^ channels and what their activating stimulus is (Marchant and Patel, 2013). TPCs were originally described as non-selective cation channels permeable to Ca^2+^ and gated by the second messenger NAADP (Brailoiu et al, 2009; Calcraft et al, 2009; Zong et al, 2009). This provided a molecular basis for NAADP-mediated Ca^2+^ release from lysosomes and other acidic organelles (Churchill et al, 2002; Galione et al, 2010; Lee and Aarhus, 1995). Independent work however described TPCs as Na^+^-selective channels activated by the lysosomal-enriched phosphoinositide, PI(3,5)P_2_, and insensitive to NAADP (Cang et al, 2013; Wang et al, 2012). Structural analyses identified the binding site for PI(3,5)P_2_ centered around a polybasic cluster within the S4-S5 linker of the first repeat of the channel (Patel, 2018; She et al, 2018; She et al, 2019). And we now know that NAADP regulates TPCs indirectly through the recently identified accessory NAADP binding proteins JPT2 and Lsm12 (Gunaratne et al, 2021; Gunaratne et al, 2023; Marchant et al, 2021; Roggenkamp et al, 2021; Zhang et al, 2021), loss of which might explain NAADP insensitivity in certain experimental settings. Parallel analyses of TPC2 under conditions where channel activity to both NAADP and PI(3,5)P_2_ was preserved yielded the very distinct agonist-evoked cation permeabilities reported in foundational analyses (Gerndt et al, 2020a). Moreover, the contrasting effects of NAADP and PI(3,5)P_2_ could be reliably mimicked by structurally distinct small molecules, TPC2-A1-N and TPC2-A1-P identified in a high throughput screen (Gerndt et al, 2020a). These lipophilic agonists had differential effects on lysosomal activity in live cells thereby linking distinct ionic profiles to unique functional outcomes (Gerndt et al, 2020b).

Yet more unusual channel behavior has emerged from findings that despite such marked segregation in the biophysics and downstream physiology of TPC2 evoked by its agonists, co-activation selectively favors its Ca^2+^ signaling modality (Yuan et al, 2022). Thus, Ca^2+^ but not Na^+^ permeability is synergistically increased by agonist combinations, be them natural or synthetic, even though individually the agonists signal very differently. This translated into synergistic increases in lysosomal pH and decreases in organelle motility (Yuan et al, 2022) and global Ca^2+^ signalling resulting from cross-talk with neighboring Ca^2+^ channels on the endoplasmic reticulum (Yuan et al, 2024). The permeability of TPC2 in response to its agonists is thus anything but fixed, results supported by recent computational analyses revealing marked structural plasticity of the selectivity filter (Zaki et al, 2024). These findings challenge the textbook view of ion channel workings.

But how is TPC2 so dramatically swayed by its agonists? Using site-directed mutagenesis in conjunction with single cell Ca^2+^ imaging, patch clamp electrophysiology, molecular dynamics simulations and transgenic animals, we reveal that key residues remote from the selectivity filter control ionic flux. We convert TPC2 into a ‘regular’ ion channel unable to distinguish its agonists and with properties resembling the co-activated state. Associated increased Ca^2+^ permeability and toxicity in vivo lead us to propose that agonist-dependent ion permeability changes in TPC2 constitute an essential organellar safeguard mechanism.

## Results

### Identification of gain-of-function mutations in TPC2

**Figure 1:**
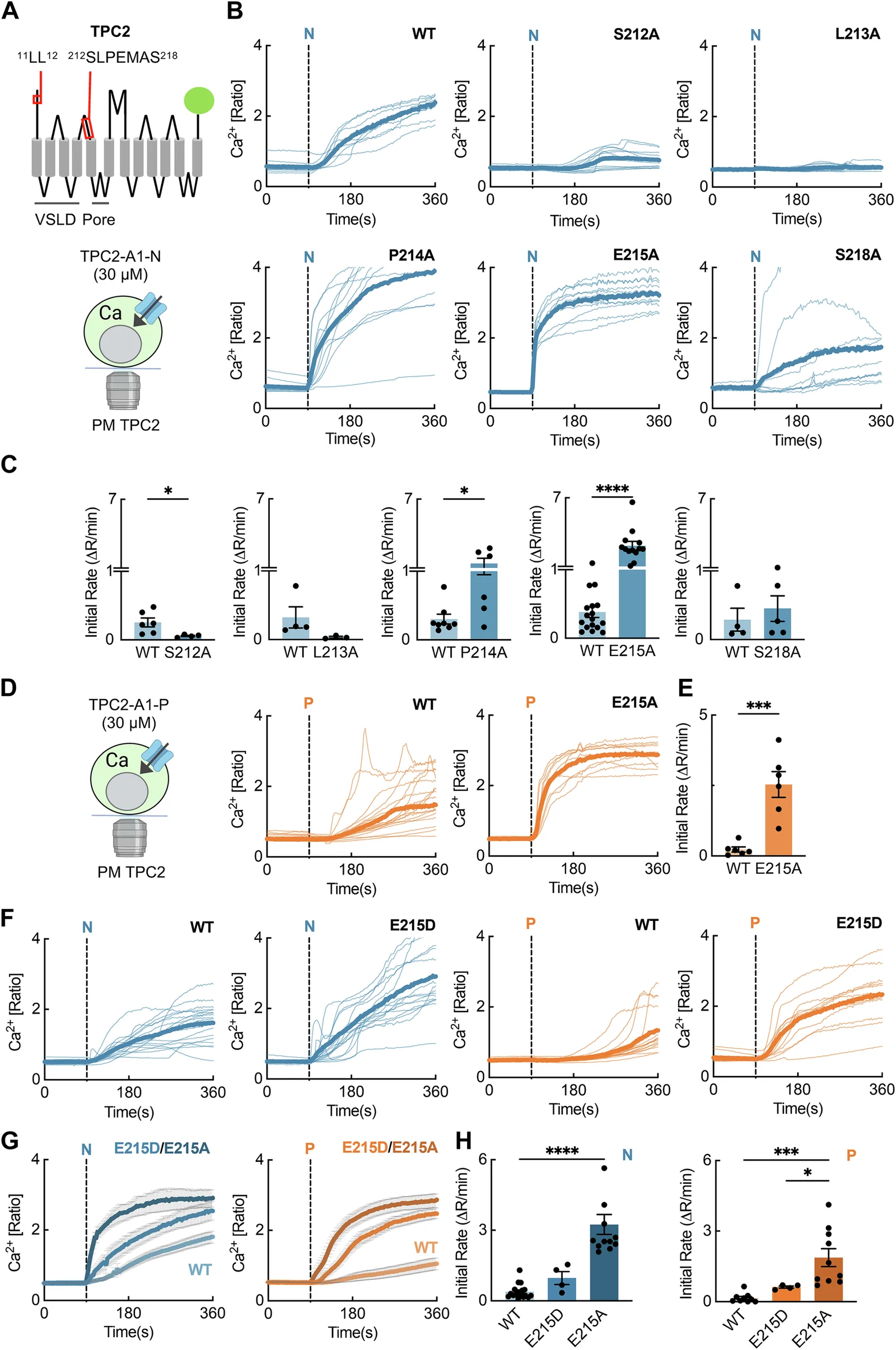
Identification of gain-of-function mutations in TPC2. (**A**) Schematic depicting the structure of an individual subunit of TPC2, the sequence subject to alanine scanning and the protocol used to infer channel activity using the functional NAADP mimetic TPC2-A1-N. Experiments were performed in an extracellular-like solution containing 2 mM Ca^2+^. (**B**) Ca^2+^ signals to TPC2-A1-N recorded from HeLa cells expressing the indicated TPC2 construct at the plasma membrane. Cells were loaded with the ratiometric Ca^2+^ indicator, Fura-2 and stimulated with 30 µM TPC2-A1-N. Each trace is the fluorescence ratio imaged from a single GFP-positive cell from a typical field of view. The thicker trace is the average of the population. (**C**) Pooled data (mean ± s.e.m.) quantifying the initial rate of change of the Fura-2 ratio from 3 to 17 independent experiments where each point represents the mean response from an individual population. *P = 0.04, 0.02 (left to right), ****P < 0.0001 (unpaired *t* test). (**D**) Ca^2+^ signals to a functional PI(3,5)P_2_ mimetic recorded from HeLa cells expressing wild-type TPC2 or the E215A mutant at the plasma membrane. Cells were loaded with the ratiometric Ca^2+^ indicator, Fura-2 and stimulated with 30 µM TPC2-A1-P in the presence of 2 mM extracellular Ca^2+^. Each trace is the fluorescence ratio imaged from a single GFP-positive cell from a typical field of view. The thicker trace is the average of the population. (**E**) Pooled data (mean ± s.e.m.) quantifying the initial rate of change of the Fura-2 ratio from 6 independent experiments where each point represents the mean response from an individual population. ***P = 0.003 (unpaired *t* test). (**F**) Ca^2+^ signals to TPC2 agonists recorded from HeLa cells expressing wild-type TPC2 or the E215D mutant at the plasma membrane. Cells were loaded with the ratiometric Ca^2+^ indicator, Fura-2 and stimulated with 30 µM TPC2-A1-N or 30 µM TPC2-A1-P. Each trace in (F) is the fluorescence ratio imaged from a single GFP-positive cell from a typical field of view. The thicker trace is the average of the population. (**G**) Pooled data (mean ± s.e.m.) comparing time-courses of agonist action in cells expressing the E215D mutant in TPC2 with E215A from where each point combines averages from multiple independent cell populations (*n* > 4). (**H**) Pooled data (mean ± s.e.m.) quantifying the initial rate of change of the Fura-2 ratio from >  4 independent experiments where each point represents the mean response from an individual population. *P = 0.04, ***P = 0.0005, ****P < 0.0001 (Kruskal–Wallis test (left) or one-way ANOVA (right) followed by Dunn’s post hoc test). Source data are available online for this figure.

To explore mechanisms underpinning agonist activation of TPC2, we performed site-directed mutagenesis and measured channel activity. Our previous study identified residues in and around the PI(3,5)P_2_ binding site as crucial for channel activation harboring both agonist-selective and pan-agonist molecular determinants (Saito et al, 2023). Here we report the results of an alanine scan of a nearby region between the distal, C-terminus of the S4-S5 linker and the proximal, N-terminus of the S5 helix in domain I (S212–S218) (Fig. 1A). All mutations were performed in the background of L11A and L12A to re-direct the channel to the cell surface (Brailoiu et al, 2010).

**Figure EV1:**
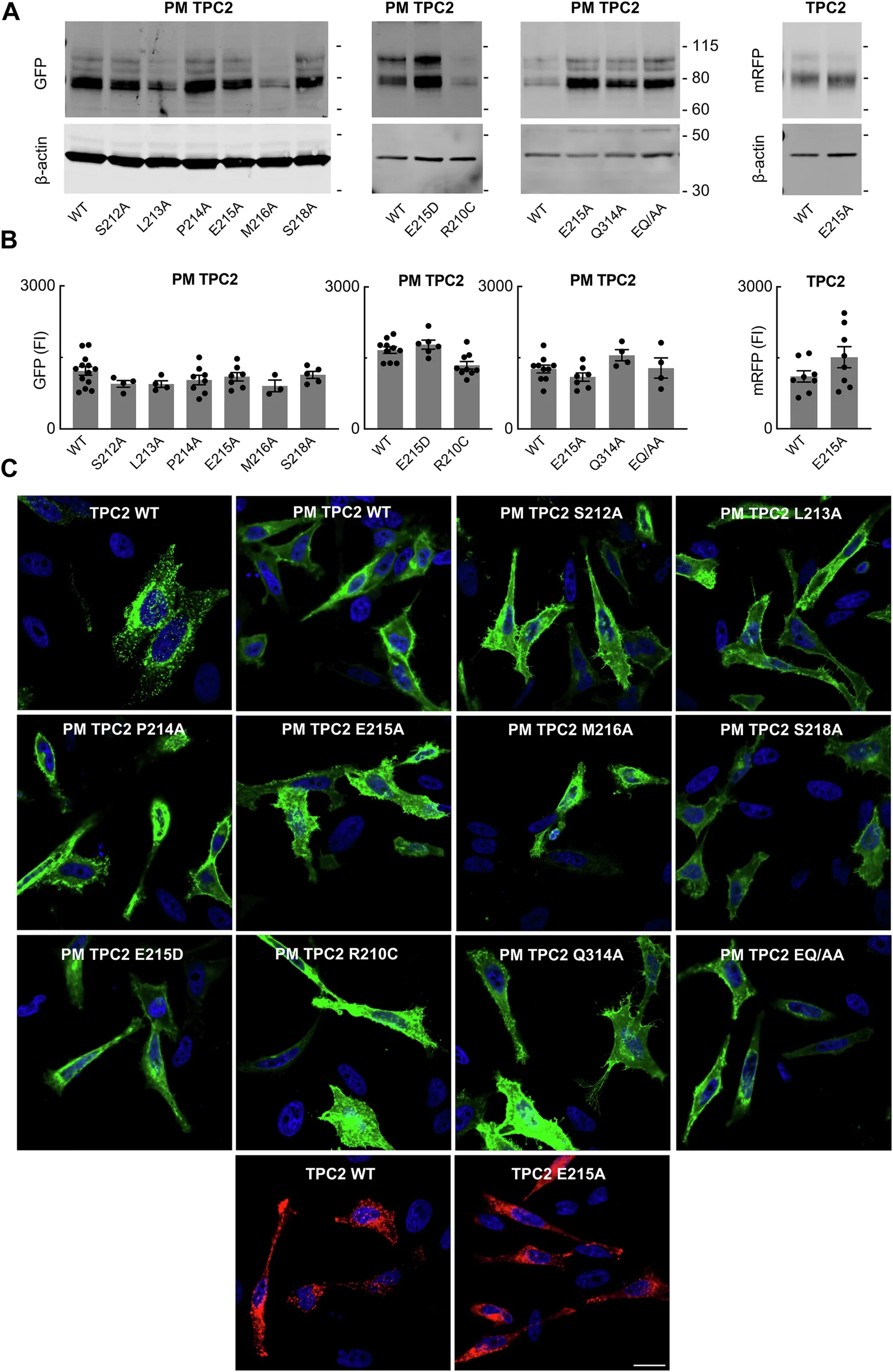
Expression analysis of recombinant TPC2. (**A**–**C**) HeLa cells were transiently transfected with GFP-tagged TPC2^L11A/L12A^ (PM TPC2) or mRFP-tagged TPC2 (TPC2) with and without (WT) the additional indicated mutation. Western blot analyses of cell lysates expressing TPC2 using an antibody to GFP, mRFP or β-actin (**A**). Data are representative of three experiments. Fluorescence intensity (FI) analysis of individual cells expressing TPC2 (**B**). Pooled data (mean ± s.e.m.) quantifying GFP or mRFP fluorescence from 3 to 23 independent experiments where each point represents the mean intensity from an individual population. Confocal analyses of PM (green) and lysosomal (red)-targeted TPC2 constructs (**C**). Nuclei were stained with DAPI (blue). Scale bar 20 µm. Data are representative of three experiments.

Figure EV1A shows the results of western blotting of lysates prepared from cells transiently expressing TPC2^L11A/L12A^ (here on referred to as PM TPC2 for plasma membrane) with the various mutants tagged with GFP at their C-termini. All constructs were readily expressed except the M216A mutant. To quantify channel expression in individual cells, we took two approaches. In the first, we measured fluorescence intensity of the GFP fusion tag for each of the constructs. As shown in Fig. EV1B, fluorescence of the cells expressing PM TPC2 was similar to that in cells expressing it with the additional mutation. In the second approach, we performed confocal microscopy to determine the subcellular location of the constructs. As shown in Fig. EV1C, all the mutants appeared to traffic to the PM.

To activate TPC2, we used the novel functional NAADP mimetic TPC2-A1-N (Gerndt et al, 2020a). Channel activity was inferred by measuring cytosolic Ca^2+^ levels with the ratiometric fluorescent Ca^2+^ indicator Fura-2 in individual GFP-positive cells upon agonist challenge (Fig. 1A). As shown in Fig. 1B, stimulation of cells expressing PM TPC2 with 30 µM TPC2-A1-N in a typical field of view resulted in readily detectable increase in the fluorescence ratio consistent with Ca^2+^ entry into the cell as reported previously (Gerndt et al, 2020a; Yuan et al, 2022). In contrast, cells expressing PM TPC2^S212A^ or PM TPC2^L213A^ were largely unresponsive. Responses to TPC2-A1-N of cells expressing PM TPC2^P214A^ were readily detectable and were quicker compared to controls. Strikingly, mutation of E215 resulted in a further pronounced increase in the rate of Ca^2+^ entry in response to TPC2-A1-N. We noted that in the limited single cells that were demonstrably GFP-positive for PM TPC2^M216A^, basal Ca^2+^ levels were markedly elevated (Appendix Fig. S1). Responses to TPC2-A1-N of cells expressing PM TPC2^S218A^ were similar to controls. Overall, there was variability in the responses between cells likely due to transient transfection. Data from single cells was therefore averaged across the field of view and experiments performed multiple times to generate a series of independent population averages based on single cell parameters for statistical analyses. Summary data quantifying the initial rate of change in this way are shown in Fig. 1C.

**Figure EV2:**
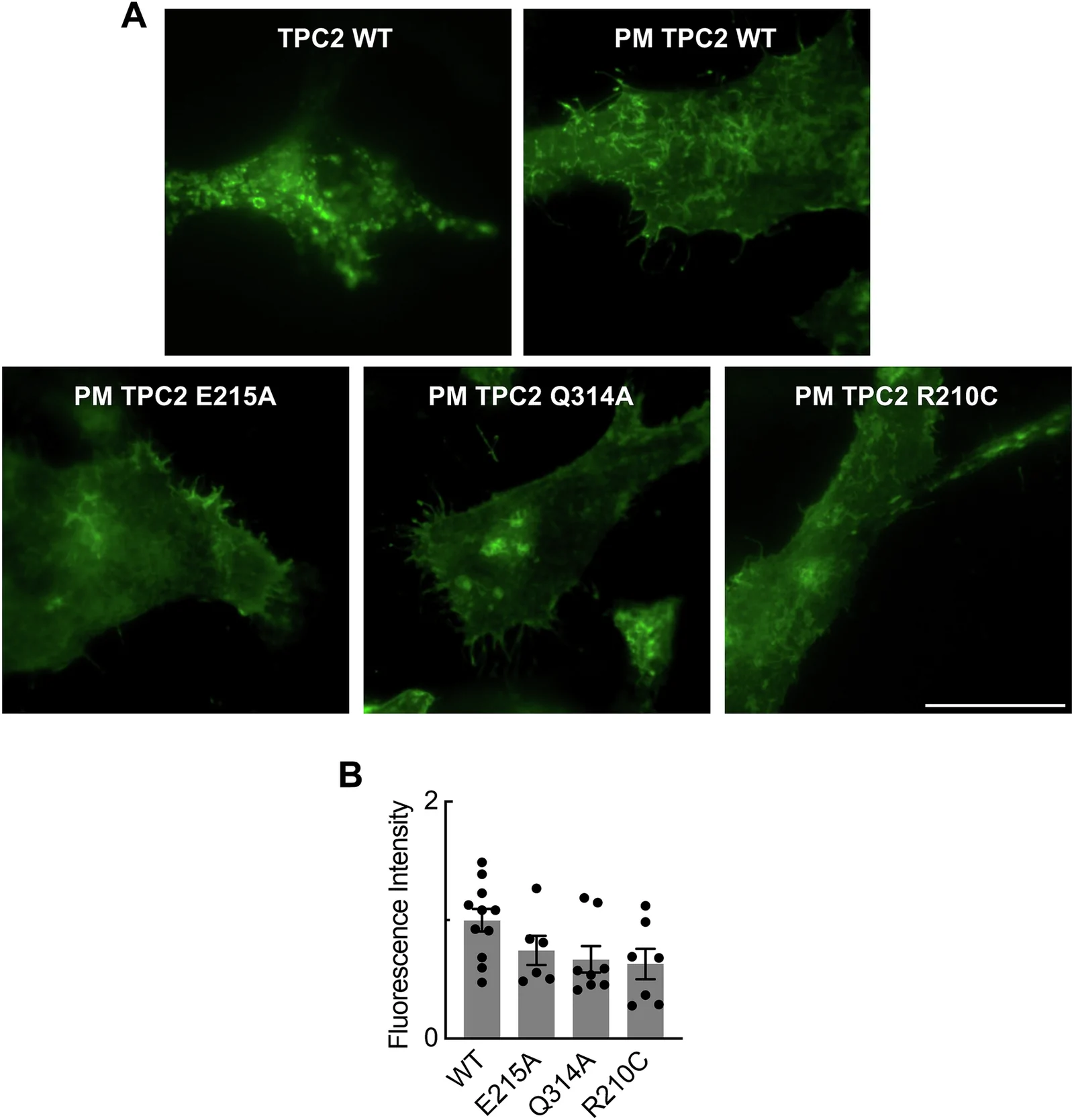
Expression analysis of plasma membrane-targeted TPC2. (**A**) Exemplar TIRF images of the indicated GFP-tagged TPC2 constructs expressed in HeLa cells. Scale bar 20 µm. (**B**) Pooled data (mean ± s.e.m.) quantifying fluorescence from two independent transfections where each point represents the intensity from an individual cell expressing the indicated plasma membrane-targeted mutant. Data are normalised to wild-type PM TPC2.

We further characterized the properties of the E215 mutant by comparing the effects of the functional PI(3,5)P_2_ mimetic TPC2-A1-P (Fig. 1D). TPC2-A1-P renders TPC2 largely Na^+^-selective but Ca^2+^ influx is detectable in cells expressing TPC2 re-routed to the plasma membrane. As reported previously (Gerndt et al, 2020a), TPC2-A1-P induced sluggish Ca^2+^ signals in cells expressing PM TPC2 (Fig. 1D). In contrast, TPC2-A1-P induced rapid and more robust signals in cells expressing PM TPC2^E215A^. These data, summarized in Fig. 1E, provide additional evidence for a gain-of-function toward both synthetic agonists. No significant differences were found in expression of PM TPC2 and PM TPC2^E215A^ using TIRF microscopy to assess expression at the cell surface (Fig. EV2).

We also examined the effect of mutating E215 to aspartate thereby preserving the negative charge at this position. As shown in Fig. 1F, the responses to both TPC2-A1-N and TPC2-A1-P in cells expressing PM TPC2^E215D^ were accelerated relative to PM TPC2. But the rate of Ca^2+^ entry was less potentiated than in cells expressing PM TPC2^E215A^ (Fig. 1G,H). These data indicate that gain-of-function upon mutation of E215 to alanine is likely driven by disruption of electrostatic interactions.

In sum, the S4-S5 linker is a key regulator of channel activity harboring residues that temper channel activity.

### Gain-of-function reduces agonist-selective changes in TPC2 permeability

**Figure 2:**
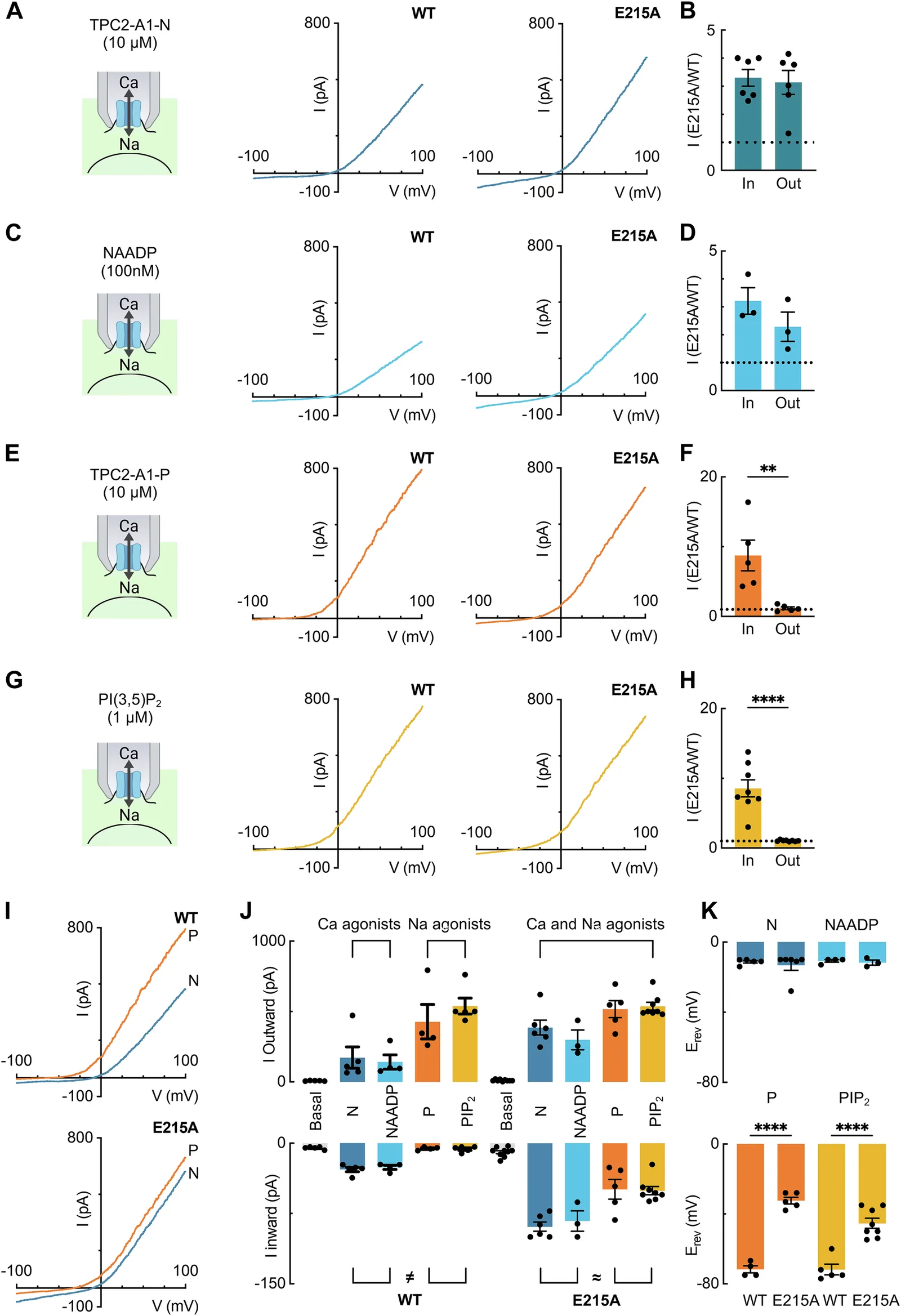
Gain-of-function reduces agonist-selective changes in TPC2 permeability. (**A**–**D**) Effect of NAADP or its mimetic on currents from HEK cells expressing wild-type TPC2 or the E215A mutant at the plasma membrane. Macropatches were stimulated with 10 µM TPC2-A1-N (**A**) or 100 nM NAADP (**C**) under bi-ionic conditions using a pipette (luminal) solution containing 105 mM CaCl_2_, 5 mM HEPES and 5 mM MES (pH 4.6) and a bath (cytosolic) solution containing 160 mM NaCl and 5 mM HEPES (pH 7.2). Exemplar recordings are shown. Pooled data (mean ± s.e.m.) quantifying the fold-increase in the inward Ca^2+^ currents at -100 mV or the outward Na^+^ currents at +100 mV in cells expressing the mutant channel versus the wild-type in response to TPC2-A1-N (**B**) or NAADP (**D**). Data are from three to six independent experiments where each point represents the response of an individual patch. Unpaired *t* test. (**E**–**H**) Effect of PI(3,5)P_2_ and its mimetic on currents from HEK cells expressing wild-type or the E215A mutant at the plasma membrane. Macropatches were stimulated with 10 µM TPC2-A1-P (**E**) or 1 µM PI(3,5)P_2_ (**G**) under bi-ionic conditions. Exemplar recordings are shown. Pooled data (mean ± s.e.m.) quantifying the fold-increase in the inward Ca^2+^ currents at -100 mV and the outward Na^+^ currents at +100 mV in cells expressing the mutant channel versus the wild-type in response to TPC2-A1-P (**F**) or PI(3,5)P_2_ (**H**). Data are from five to eight independent experiments where each point represents the response of an individual patch. **P = 0.009, ****P < 0.0001 (unpaired *t* test). (**I**–**K**) Comparison of the effects of TPC2 activators on currents from HEK cells expressing wild-type or the E215A mutant at the plasma membrane. Exemplar recordings for TPC2-A1-N and TPC2-A1-P overlaid in (**I**) are the same as those shown in (**A**, **E**), respectively. Pooled data (mean ± s.e.m.) quantifying the amplitudes of the inward Ca^2+^ currents at −100 mV and the outward Na^+^ currents at +100 mV (**J**) and the reversal potentials (**K**) in cells expressing the mutant channel versus the wild-type in response to the indicated agonist. Data are from three to eight independent experiments where each point represents the response of an individual patch. ****P < 0.0001 (one-way ANOVA followed by Dunnett’s post hoc test). Source data are available online for this figure.

To directly assess channel activity, we performed electrophysiology of TPC2 in macropatches excised from the plasma membrane. These experiments were performed under bi-ionic conditions (luminal Ca^2+^, cytosolic Na^+^) as reported previously (Gerndt et al, 2020a; Saito et al, 2023; Yuan et al, 2022) to allow measurement of the small Ca^2+^ currents relative to the larger Na^+^ currents. As shown in Fig. 2A, TPC2-A1-N evoked both inward Ca^2+^ currents and outward Na^+^ currents in cells expressing PM TPC2. Similar experiments were performed using PM TPC2^E215A^. The Ca^2+^ currents to TPC2-A1-N were significantly enhanced consistent with the Ca^2+^ imaging experiments. So too were the Na^+^ currents. To determine whether enhanced currents were a feature of TPC2 stimulated with its natural agonist, we performed patch clamp experiments with NAADP. As shown in Fig. 2C, both the Ca^2+^ and Na^+^ currents were increased in the E215A mutant compared to the wild-type channel. Figure 2B,D quantifies the relative change in the currents upon mutation of E215 in response to TPC2-A1-N and NAADP. Both the Ca^2+^ and Na^+^ currents were increased approximately threefold in response to TPC2-A1-N and NAADP.

Electrophysiological analysis of TPC2 stimulated with TPC2-A1-P is shown in Fig. 2E. For the wild-type channel, TPC2-A1-P evoked small Ca^2+^ currents but large Na^+^ currents. These markedly different biophysical profiles for TPC2-A1-P compared to TPC2-A1-N are consistent with our previous analyses using both plasma membrane and vacuolar targeted TPC2 (Yuan et al, 2022). Robust Ca^2+^ currents were readily observed when PM TPC2^E215A^ was challenged with TPC2-A1-P thus confirming the robust Ca^2+^ signals (Fig. 2E). In stark contrast, the Na^+^ currents evoked by TPC2-A1-P were only minimally affected by mutation. Essentially similar results were obtained when PM TPC2^E215A^ was challenged with PI(3,5)P_2_ (Fig. 2G). Figure 2F,H quantifies the relative change in the currents upon mutation of E215 in response to TPC2-A1-P and PI(3,5)P_2_. Ca^2+^ currents were increased approximately tenfold whereas the Na^+^ currents were largely unchanged.

The differential effects of the mutation on the currents rendered the channel highly permeable regardless of the stimulating agonist. This was most apparent for the synthetic agonists where upon mutation the currents evoked by TPC2-A1-N and TPC2-A1-P were very similar (Fig. 2I). A summary of current amplitudes for the two permeant ions recorded from the wild-type and mutant channel in response to all four agonists is plotted in Fig. 2J. The Ca^2+^ currents were larger for NAADP and TPC2-A1-N (‘Ca^2+^ agonists’) than for PI(3,5)P_2_ and TPC2-A1-P in the wild-type channel. But they were similar in the mutant channel. Conversely, the Na^+^ currents for PI(3,5)P_2_ and TPC2-A1-P (‘Na agonists’) were larger than for NAADP and TPC2-A1-N in the wild-type channel. But they were again similar in the mutant channel. Thus, the E215A mutation substantially reduces the ability of TPC2 to discriminate its activators.

To further interrogate the properties of the mutant, we compared reversal potentials (E_rev_). As shown in Fig. 2K, E_rev_ for NAADP and TPC2-A1-N was ~−11 mV corresponding to a *P*_Ca_/*P*_Na_ of ~0.6. In contrast, the E_rev_ for PI(3,5)P_2_ and TPC2-A1-P was negatively shifted to ~−72 mV corresponding to a *P*_Ca_/*P*_Na_ of ~0.04. These data are consistent with the pronounced agonist-dependent plasticity for the wild-type channel (Gerndt et al, 2020a). Mutation did not affect the E_rev_ for the Ca^2+^ agonists but it caused a significant positive shift for the Na^+^ agonists (Fig. 2J). Thus, interfering with a residue remote from the selectivity filter alters the permeability of TPC2 much like its activators.

In sum, E215 controls the ability of TPC2 to change its cation permeability in an agonist-selective manner.

### Gain-of-function reduces synergistic activation of TPC2 by its agonists

**Figure 3:**
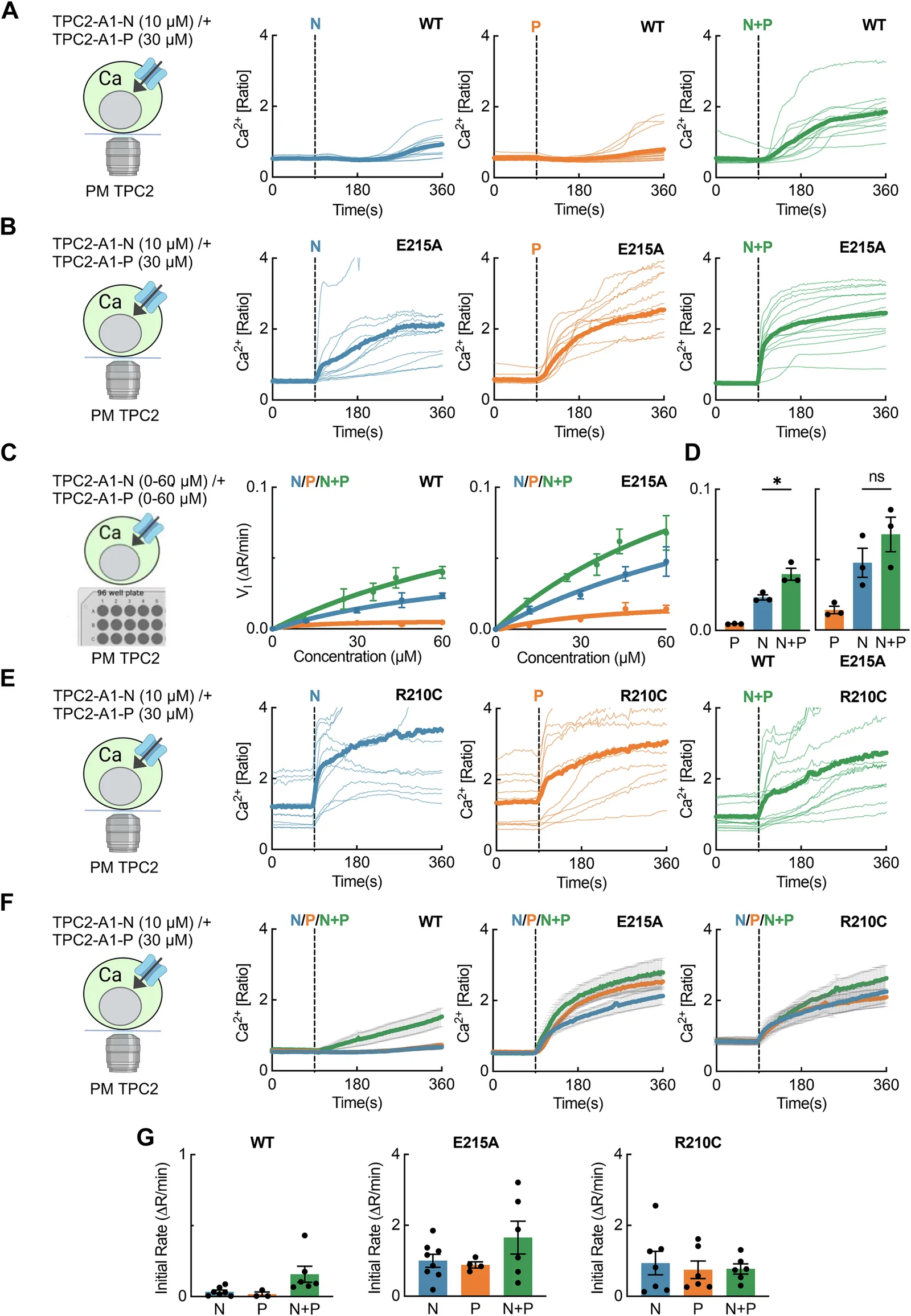
Gain-of-function reduces synergistic activation of TPC2 by its agonists. (**A**, **B**) Ca^2+^ signals to synthetic TPC2 activators recorded from HeLa cells expressing wild-type TPC2 (**A**) or the E215A mutant (**B**) at the plasma membrane. Cells were loaded with the ratiometric Ca^2+^ indicator, Fura-2 and stimulated with 10 µM TPC2-A1-N, 30 µM TPC2-A1-P or a combination of the two in the presence of 2 mM extracellular Ca^2+^. Each trace is the fluorescence ratio imaged from a single GFP-positive cell from a typical field of view. The thicker trace is the average of the population. (**C**) Effect of increasing concentrations of synthetic TPC2 activators and their combinations on the rate of Ca^2+^ entry in HeLa cells expressing wild-type TPC2 or the E215A mutant at the plasma membrane. Cells were loaded with the low affinity ratiometric Ca^2+^ indicator, Fura-FF and stimulated with TPC2-A1-N and TPC2-A1-P alone or together at the indicated concentrations using an automated plate reader in the presence of 2 mM extracellular Ca^2+^. Data are expressed as mean ± s.e.m. from three experiments. (**D**) Pooled data (mean ± s.e.m.) quantifying the initial rate of change of the Fura-FF ratio in response to 60 µM TPC2 agonist(s) from 3 independent experiments where each point represents the mean response from an individual plate. *P = 0.01 (one-way ANOVA followed by Dunn’s post hoc test). (**E**) Ca^2+^ signals to TPC2 agonists recorded from HeLa cells expressing the R210C mutant at the plasma membrane. Cells were loaded with the ratiometric Ca^2+^ indicator, Fura-2 and stimulated with 30 µM TPC2-A1-N, 30 µM TPC2-A1-P or a combination of the two in the presence of 2 mM extracellular Ca^2+^. Each trace is the fluorescence ratio imaged from a single GFP-positive cell from a typical field of view. The thicker trace is the average of the population. (**F**) Pooled time course data (mean ± s.e.m.) from three to eight independent experiments comparing agonist action in cells expressing the R210C mutant in TPC2 with E215A where each point combines averages from multiple independent cell populations. (**G**) Pooled data (mean ± s.e.m.) quantifying the initial rate of change of the Fura-2 ratio from 3 to 28 independent experiments where each point represents the mean response from an individual population expressing wild-type TPC2 or the indicated mutant. Source data are available online for this figure.

The selective effect of the mutation on Ca^2+^ currents for TPC2-A1-P and PI(3,5)P_2_ (Fig. 2E–G) is highly reminiscent of the effect of agonist combinations on the wild-type channel reported recently (Yuan et al, 2022) whereby co-activation with TPC2 agonists synergize to increase Ca^2+^ but not Na^+^ flux. So too is the selective effect of the mutation on E_rev_ for Na^+^ agonists versus the Ca^2+^ agonists (Fig. 2K). Only E_rev_ for the latter was significantly increased (less positive) again reminiscent of the intermediate relative permeability of the co-activated channel (Yuan et al, 2022). This congruency prompted us to compare the effect of agonist combinations on PM TPC2 and PM TPC2^E215A^. Consistent with our previous analysis, co-stimulation of TPC2 with a low concentration of TPC2-A1-N together with TPC2-A1-P markedly accelerated Ca^2+^ influx in cells expressing PM TPC2 (Fig. 3A). But in cells expressing PM TPC2^E215A^, the synergistic effect of the agonists combination was lost such that co-stimulation only modestly increased the rate of Ca^2+^ entry (Fig. 3B).

One potential concern was that synergism for the mutant may have been missed due to the robust nature of the Ca^2+^ influx precipitated by the gain-of-function. To address this, we used the low affinity Ca^2+^ indicator Fura-FF (to prevent possible probe saturation) and automated plate-reading in conjunction with microfluidics (to establish full concentration-effect relationships). Ca^2+^ influx was measured in response to TPC2-A1-N, TPC2-A1-P, and a combination of the two. As shown in Fig. 3C, TPC2-A1-N increased the rate of Ca^2+^ influx in a concentration-dependent manner in cell populations expressing PM TPC2. TPC2-A1-P effects were not readily detectable under these conditions. But the agonist combinations significantly increased Ca^2+^ influx. In cells expressing the PM TPC2^E215A^, TPC2-A1-N-induced Ca^2+^ influx was faster than in the wild-type cells and TPC2-A1-P effects were now resolvable. Agonist combinations again accelerated influx but the effects were more modest. At 60 µM for example, the rate of Ca^2+^ influx in response to agonist combination was approximately twofold greater than with TPC2-A1-N alone in the control cells. But in the mutant cells, the increase was < twofold, not statistically significant from TPC2-A1-N alone and could be accounted for influx mediated by TPC2-A1-P (Fig. 3D). In other words, Ca^2+^ influx in the mutant cells was not synergistic but additive.

Recent work linked a mutation in TPC2 to albinism (Wang et al, 2023). The mutation (R210C) is in the proximal S4-S5 linker embedded within the PI(3,5)P_2_ binding site and reportedly a gain-of-function. We hypothesized that this mutation perturbs agonist action in a similar way to the E215A mutant. To test this, we examined the effects of TPC2-A1-N and TPC2-A1-P, both singly and in combination in cells expressing PM TPC2^R210C^. Western blot analysis of cell populations indicated that the R210C mutant did not express well (Fig. EV1A). Nevertheless, the response to TPC2-A1-N or TPC2-A1-P in single cells was enhanced compared to cells expressing PM TPC2 at equivalent levels (Fig. 3E). These data confirm that R210C stimulates TPC2 activity. Importantly, the agonist combination did not further accelerate Ca^2+^ entry in cells relative to either agonist alone (Fig. 3E). These data, summarized in Fig. 3F,G, show that synergistic agonist action at TPC2 was again disrupted similar to TPC2 E215A. Like the M216A mutant, basal Ca^2+^ levels in cells expressing PM TPC2^R210C^ were elevated (Appendix Fig. S1). albeit more modestly and pointing to constitutive activity for both mutants.

In sum, loss of agonist-selective changes in cation permeability is associated with a co-activated like channel state unable to support synergistic activation.

### Functional interactions between the S4-S5 linker and channel activation gate

**Figure 4:**
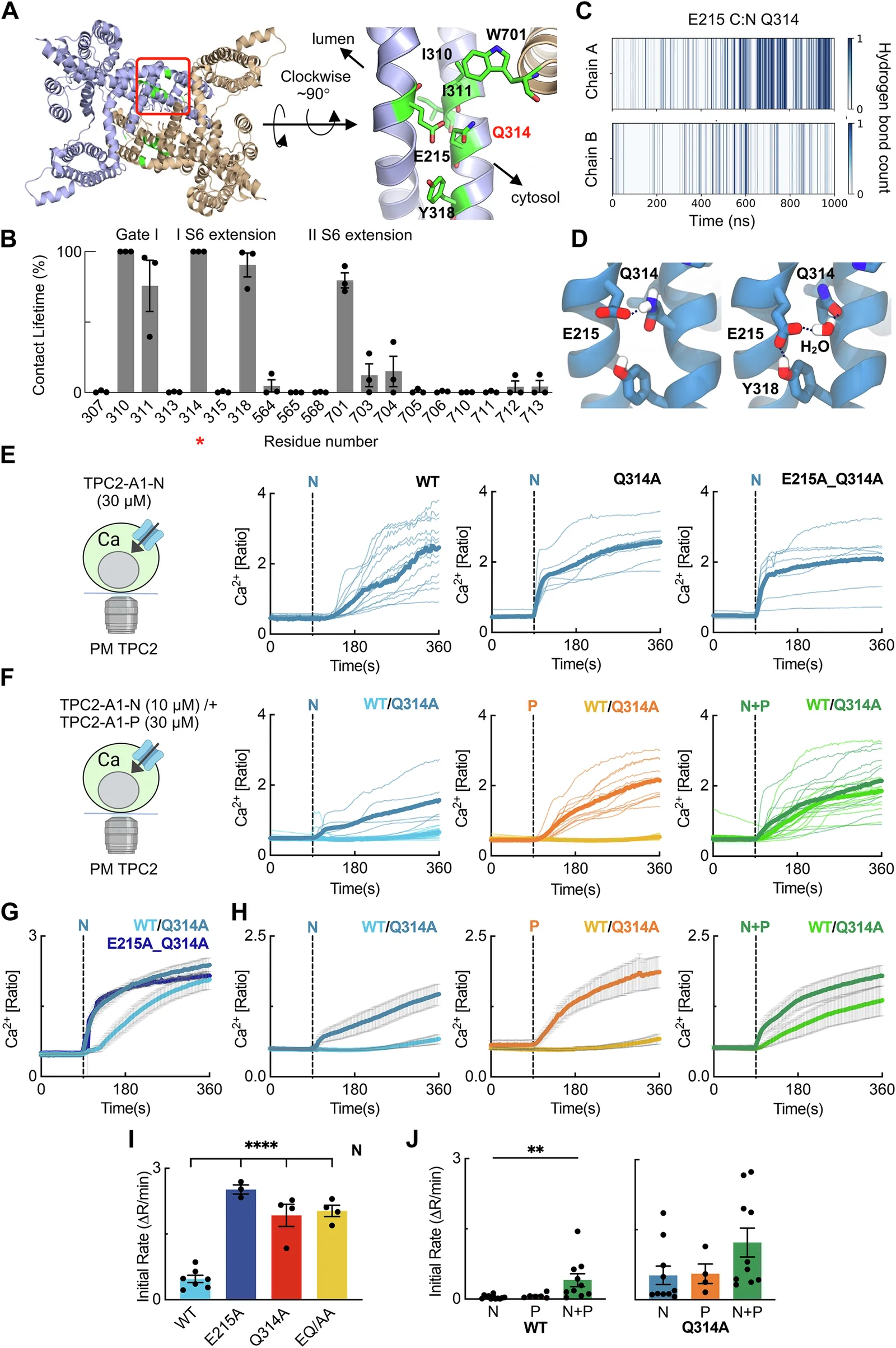
Functional interactions between the S4-S5 linker and channel gate control TPC2 activation. (**A**) Cryo EM structure of TPC2 highlighting E215, neighbouring gating residues (I310 and I311) and residues in the S6 extension of repeat I (Q314, W318) and repeat II (W701). (**B**) Contact lifetime analysis of E215 with the indicated residues in TPC2. A contact was recorded when the minimum distance between E215 and the nearby residue was within 4 Å. Data (mean ± s.e.m.) are from three independent 1 μs simulations. (**C**) Hydrogen bond formation between E215 and Q314 in TPC2. Shown is an exemplar 1 µs simulation (of three) quantifying the distance between the carboxy oxygen atoms of the E215 side chain and the hydrogen of the Q314 sidechain nitrogen with time. (**D**) Direct and indirect interactions between E215 and Q314 in TPC2. Shown are examples of direct hydrogen bonding (left) and indirect, water-mediated hydrogen bonding (right). (**E**) Ca^2+^ signals to TPC2-A1-N recorded from HeLa cells expressing wild-type TPC2 or the indicated single or double mutant at the plasma membrane. Cells were loaded with the ratiometric Ca^2+^ indicator, Fura-2 and stimulated with 30 µM agonist in the presence of 2 mM extracellular Ca^2+^. Each trace is the fluorescence ratio imaged from a single GFP-positive cell from a typical field of view. The thicker trace is the average of the population. (**F**) Ca^2+^ signals to synthetic TPC2 activators recorded from HeLa cells expressing wild-type TPC2 or the Q314A mutant at the plasma membrane. Cells were loaded with the ratiometric Ca^2+^ indicator, Fura-2 and stimulated with 10 µM TPC2-A1-N, 30 µM TPC2-A1-P or a combination of the two in the presence of 2 mM extracellular Ca^2+^. Each trace is the fluorescence ratio imaged from a single GFP-positive cell from a typical field of view. The thicker trace is the average of the population. (**G**, **H**) Pooled time-course data (mean ± s.e.m.) for responses to 30 µM TPC2-A1-N (**G**) or 10 µM TPC2-A1-N, 30 µM TPC2-A1-P or a combination of the two (**H**) in cells expressing the indicated mutant. Data are from three to ten independent experiments. (**I**, **J**) Pooled data (mean ± s.e.m.) quantifying the initial rate of change of the Fura-2 ratio in response to TPC2-A1-N (**I**) or TPC2-A1-N, TPC2-A1-P or a combination of the two (**J**) in cells expressing the indicated mutant. Data are from three to ten independent experiments where each point represents the mean response from an individual population. (**I**) ****P < 0.0001 (one-way ANOVA followed by Dunn’s post hoc test). (**J**) **P = 0.001 (Kruskal–Wallis test (left) or one-way ANOVA followed by Dunn’s post hoc test (right)). Source data are available online for this figure.

To further explore the mechanism underlying deregulated agonist activation, we inspected the cryo-EM structure of TPC2 (She et al, 2019). As shown in Fig. 4A, the sidechain of E215 in the S4-S5 linker projects toward the channel gate. To investigate residue interactions that might be important in the context of the E215A mutation, we used molecular dynamics (MD) simulations to identify the lifetimes of residue-residue interactions. E215 made putative contacts with residues centered around Q314 in S6 of domain I (Fig. 4B). This includes Y318 and residues within the helix bundle of domain I (I310 and I311) and domain II of the adjacent subunit (W701). Further analysis of the proximity between E215-Q314 identified a potentially stable H-bond between the two (Fig. 4C,D). Visualization of the trajectories revealed that when E215 and Q314 were not bonded directly through a H-bond, a water-mediated interaction was formed, where both residues form a H-bond with the same water molecule (Fig. 4D; Movie EV1).

**Figure EV3:**
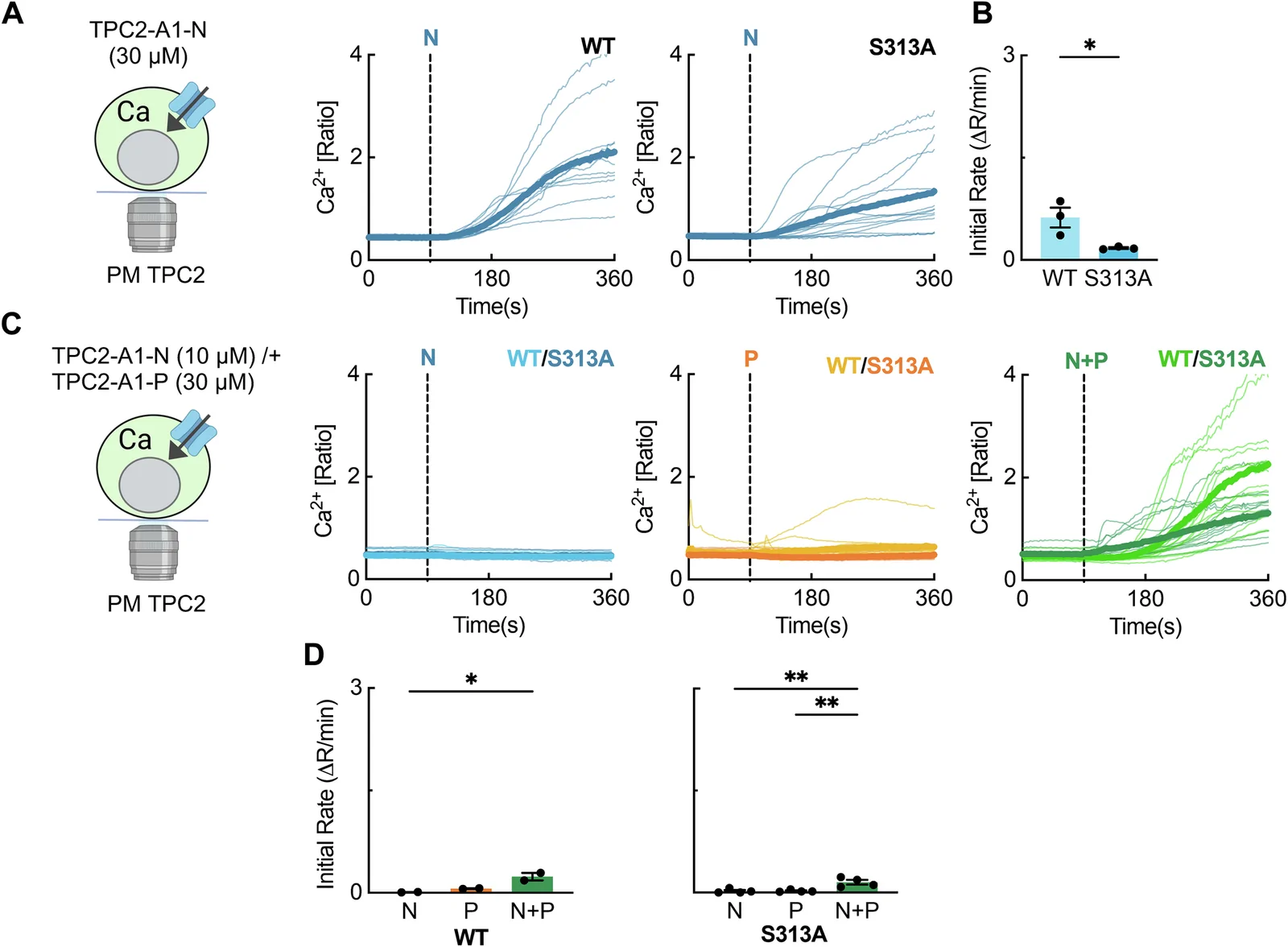
Effect of the S213A TPC2 mutation on Ca^2+^ flux. (**A**) HeLa cells expressing the indicated TPC2 construct were loaded with the ratiometric Ca^2+^ indicator, Fura-2 and stimulated with 30 µM TPC2-A1-N in the presence of 2 mM extracellular Ca^2+^. Each trace is the fluorescence ratio imaged from a single GFP-positive cell from a typical field of view. The thicker trace is the average of the population. (**B**) Pooled data (mean ± s.e.m.) quantifying the initial rate of change of the Fura-2 ratio from three independent experiments where each point represents the mean response from an individual population. *P = 0.04 (unpaired *t* test). (**C**) HeLa cells expressing the indicated TPC2 construct were loaded with the ratiometric Ca^2+^ indicator, Fura-2 and stimulated with 10 µM TPC2-A1-N, 30 µM TPC2-A1-P or a combination of the two in the presence of 2 mM extracellular Ca^2+^. Each trace is the fluorescence ratio imaged from a single GFP-positive cell from a typical field of view. The thicker trace is the average of the population. (**D**) Pooled data (mean ± s.e.m.) quantifying the initial rate of change of the Fura-2 ratio from four independent experiments where each point represents the mean response from an individual population. *P = 0.03, **P = 0.006 (one-way ANOVA followed by Dunnett’s post hoc test).

We examined the consequences of disrupting this putative interaction by mutating Q314 at the centre of the network. As shown in Fig. 4E, Ca^2+^ influx in response to TPC2-A1-N was accelerated in cells expressing the Q314A mutant relative to wild-type cells, similar to the E215A mutant. The effect was specific because mutation of the preceding residue (S313A) did not enhance Ca^2+^ entry (Fig. EV3A). To determine whether this was mechanistically linked to the effect of the E215A mutation, we examined channels harboring both mutations. As shown in Fig. 4E, TPC2-A1-N-mediated Ca^2+^ influx was accelerated in cells expressing PM TPC2^E215A/Q314A^. But the initial rate of the Ca^2+^ influx was not different to that in cells expressing the single mutations. This lack of additivity is consistent with the two residues acting in a common pathway.

To further probe the role of the Q314 mutation in TPC2 activation, we examined the effects of TPC2-A1-P alone and in combination with TPC2-A1-N. As shown in Fig. 4F, the response to TPC2-A1-P was markedly enhanced by the mutation similar to the response to TPC2-A1-N. In contrast, the response to the agonist combination was only modestly increased (Fig. 4F). Thus, like mutation of E215 and R210, mutation of Q314 also appears to prevent synergistic activation of the channel (Fig. 4G,H). Again, this effect was specific because agonists combination responses in cells expressing PM TPC2^S313A^ were similar to those of the wild-type PM channel (Fig. EV3B). Time courses and Ca^2+^ entry rates for the mutants are summarized in Fig. 4G,H and Fig. 4I,J, respectively.

**Figure EV4:**
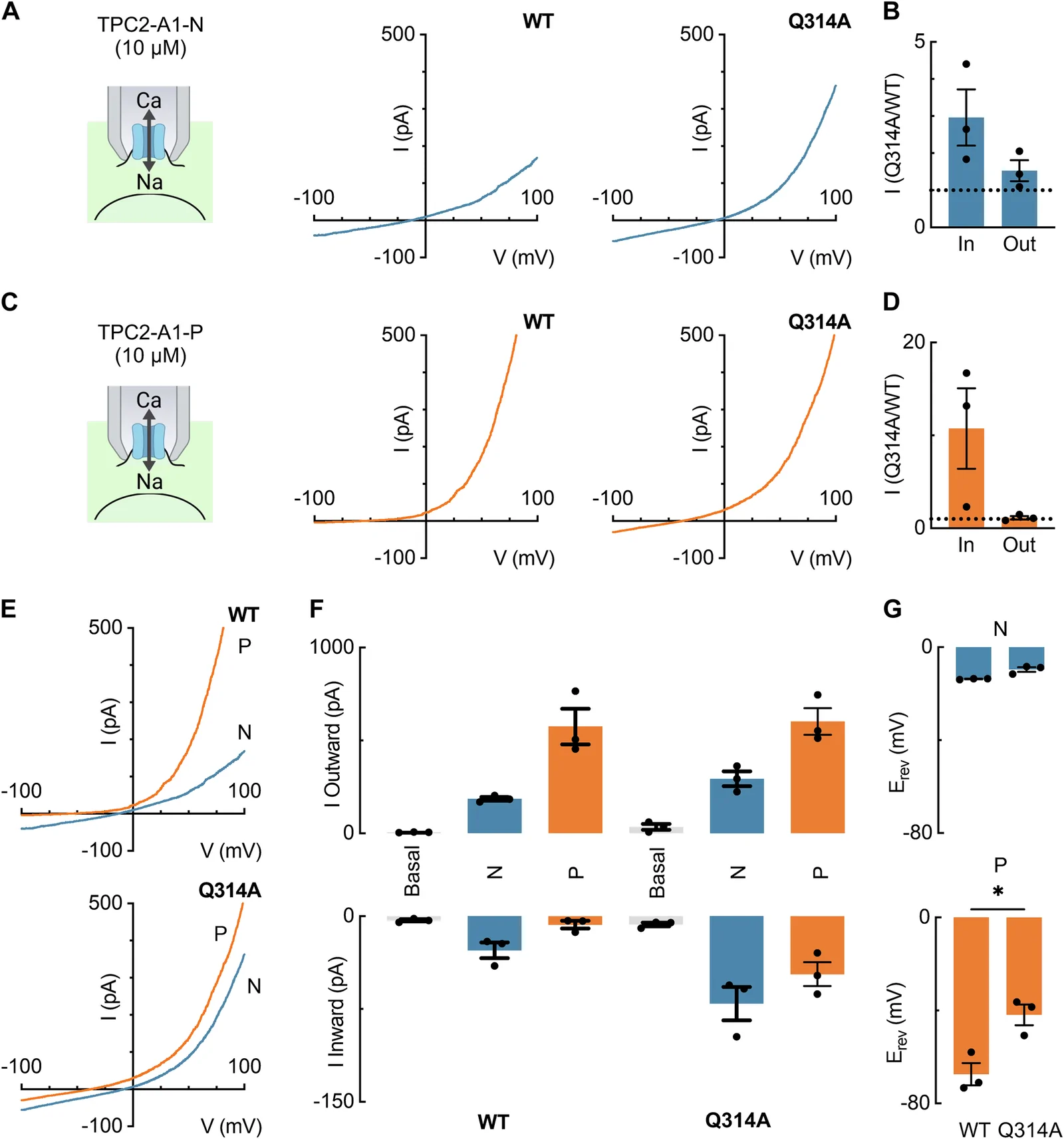
Effect of the Q314A TPC2 mutation on Na^+^ and Ca^2+^ currents. (**A**–**D**) Macropatches from HEK cells expressing the indicated TPC2 construct were stimulated with 10 µM TPC2-A1-N (**A**) or 10 µM TPC2-A1-P (**C**) under bi-ionic condition using a pipette (luminal) solution containing 105 mM CaCl_2_, 5 mM HEPES and 5 mM MES (pH 4.6) and a bath (cytosolic) solution containing 160 mM NaCl and 5 mM HEPES (pH 7.2). Exemplar recordings are shown. Pooled data (mean ± s.e.m.) quantifying the fold-increase in the inward Ca^2+^ currents at −100 mV or the outward Na^+^ currents at +100 mV in cells expressing the mutant channel versus the wild-type in response to TPC2-A1-N (**B**) or TPC2-A1-P (**D**). Data are from three independent experiments where each point represents the response of an individual patch. (**E**–**G**) Comparison of the effects of TPC2 activators on currents from HEK cells expressing wild-type or the Q314A mutant at the plasma membrane. Exemplar recordings for TPC2-A1-N and TPC2-A1-P overlaid in e are the same as those shown in (**A**, **C**), respectively. Pooled data (mean ± s.e.m.) quantifying the amplitudes of the inward Ca^2+^ currents at −100 mV and the outward Na^+^ currents at +100 mV (**F**) and the reversal potentials (**G**) in cells expressing the mutant channel versus the wild-type in response to the indicated agonist. Data are from three independent experiments where each point represents the response of an individual patch. *P = 0.02 (unpaired *t* test).

Electrophysiological analysis of PM TPC2^Q314A^ in macropatches excised from the plasma membrane is shown in Fig. EV4. As with the E215A mutation, Q314A supported larger Ca^2+^ currents to both TPC2-A1-N (Fig. EV4A,B) and TPC2-A1-P (Fig. EV4C,D) compared to the wild-type channel. TPC2-A1-P-evoked Na^+^ currents were unaffected by the mutation, thereby again rendering the channel less able to discriminate its agonists (Fig. EV4E,F). And the reversal potential for TPC2-A1-P currents was similarly positively shifted upon mutation (Fig. EV4G). Thus, PM TPC2^Q314A^ and PM TPC2^E215A^ behaved similarly at the channel level.

In sum, the channel gate harbors additional molecular determinants remote from the selectivity filter that control agonist-selective modulation of TPC2.

### Deregulated agonist activation of TPC2 perturbs lysosomal Ca^2+^ flux

Thus far, all our experiments relied on analysis of TPC2 re-routed to the plasma membrane to facilitate analysis. We next analyzed the properties of TPC2 targeted to its natural locale in the lysosome.

**Figure 5:**
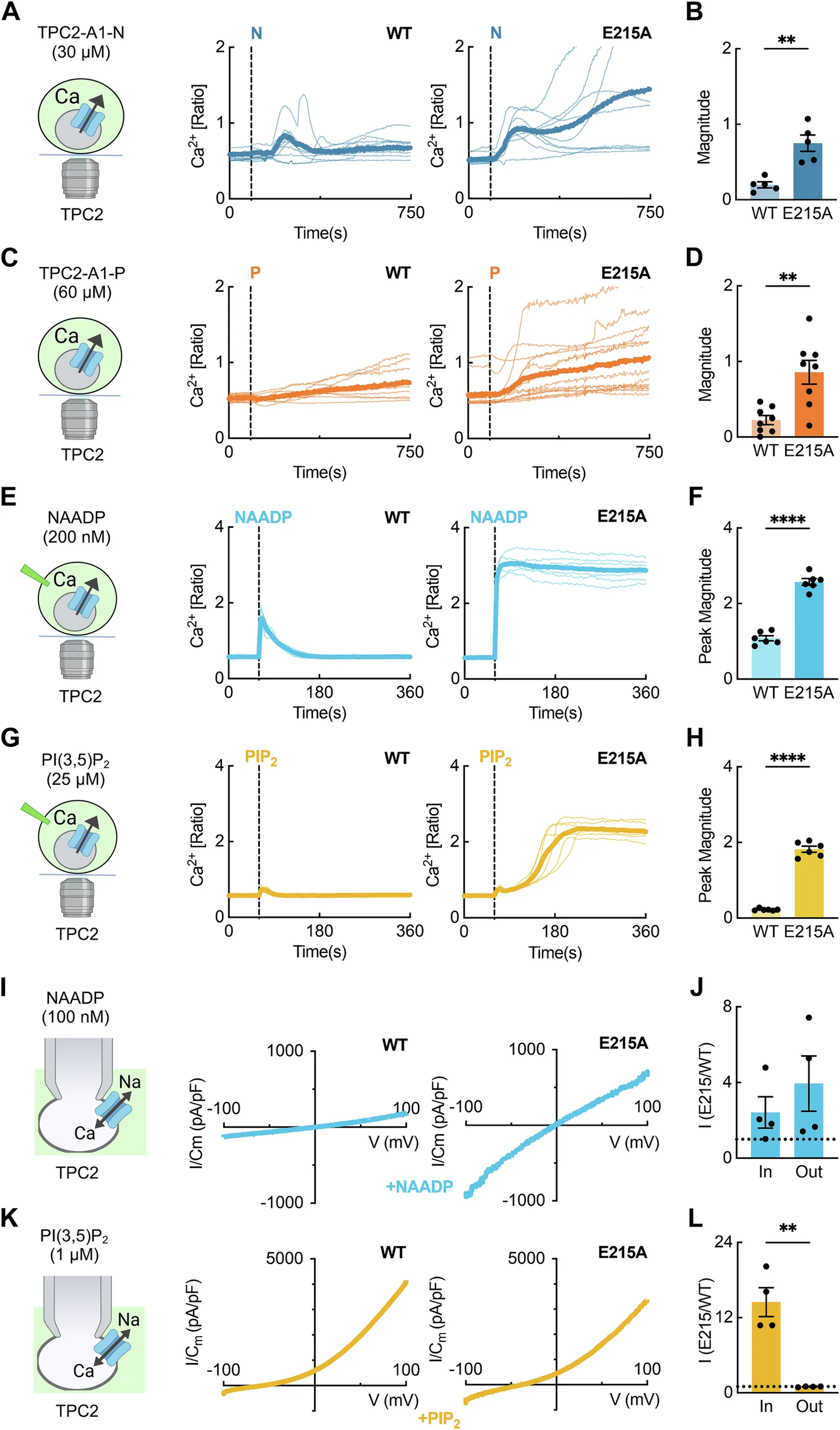
Deregulated agonist activation of TPC2 perturbs lysosomal Ca^2+^ flux. (**A**–**D**) Ca^2+^ signals to synthetic TPC2 activators recorded from HeLa cells expressing wild-type TPC2 or the E215A mutant in the lysosome. Cells were loaded with the ratiometric Ca^2+^ indicator, Fura-2 and stimulated with 30 µM TPC2-A1-N (**A**) or 60 µM TPC2-A1-P (**C**) in the presence of 2 mM extracellular Ca^2+^. Each trace is the fluorescence ratio imaged from a single mRFP-positive cell from a typical field of view. The thicker trace is the average of the population. Pooled data (mean ±  s.e.m.) quantifying the maximal change in Fura-2 ratio from five to eight independent experiments is shown in (**B**, **D**) where each point represents the mean response from an individual population. **P = 0.001, 0.002 (left to right; unpaired *t* test). (**E**–**H**) Ca^2+^ signals to endogenous TPC2 activators recorded from U2OS cells expressing wild-type TPC2 or the E215A mutant in the lysosome. Cells were loaded with the ratiometric Ca^2+^ indicator, Fura-2 and microinjected with NAADP (200 nM in the pipette, (**A**) or PI(3,5)P_2_ (25 µM in the pipette, (**C**) in the presence of 2 mM extracellular Ca^2+^. Each trace is the fluorescence ratio imaged from a single mRFP-positive cell from a typical field of view. The thicker trace is the average of all cells. Pooled data (mean ± s.e.m.) quantifying the maximal change in Fura-2 ratio from six independent experiments is shown in (**F**, **H**) where each point represents the response from an individual cell. ****P < 0.0001 (unpaired *t* test). (**I**–**L**) Vacuolar currents to endogenous TPC2 activators recorded from HEK cells expressing wild-type TPC2 or the E215A mutant in the lysosome. Enlarged vacuoles were stimulated with 100 nM NAADP (**I**) or 1 µM PI(3,5)P_2_ (**K**) under bi-ionic condition using a pipette (luminal) solution containing 105 mM CaCl_2_, 5 mM HEPES and 5 mM MES (pH 4.6) and a bath (cytosolic) solution containing 160 mM NaCl and 5 mM HEPES (pH 7.2). Exemplar recordings are shown. Pooled data (mean ± s.e.m.) quantifying the fold-increase in the inward Ca^2+^ currents at −60 mV and the outward Na^+^ currents at +100 mV in cells expressing the mutant channel versus the wild-type in response to NAADP (**J**) or PI(3,5)P_2_ (**L**). Data are from four independent experiments where each point represents the response of an individual patch. **P = 0.001 (unpaired *t* test). Source data are available online for this figure.

To measure channel activity, we compared cytosolic Ca^2+^ responses in cells expressing wild-type (non-PM-targeted) TPC2 and the corresponding E215A mutant tagged with mRFP. We first examined the effects of TPC2-A1-N and TPC2-A1-P (Fig. 5A–D). In mRFP-positive cells expressing wild-type TPC2, TPC2-A1-N evoked a modest response (Fig. 5A). This response was significantly enhanced in cells expressing TPC2^E215A^ (Fig. 5A,B). TPC2-A1-P did not evoke a response in cells expressing the wild-type channel under these conditions (Fig. 5C). But it did evoke a response comparable to that evoked by TPC2-A1-N in the mutant cells (Fig. 5C,D). These data extend Ca^2+^ flux measurements from the plasma membrane to the lysosome and confirm a gain of Ca^2+^ permeability to synthetic agonists upon mutation.

We also measured Ca^2+^ responses to its endogenous cues, NAADP and PI(3,5)P_2_ (Fig. 5E–H). For these experiments, the messengers were microinjected into live cells in real time. In control experiments expressing the wild-type channel, NAADP evoked a transient Ca^2+^ signal (Fig. 5E). In contrast, NAADP evoked a robust response in cells expressing the E215A mutant (Fig. 5E,F). Similar experiments with PI(3,5)P_2_ revealed that PI(3,5)P_2_ was without effect in cells expressing the wild-type channel. But in cells expressing TPC2^E215A^, PI(3,5)P_2_ evoked a readily detectable Ca^2+^ signal with a slow onset (Fig. 5G,H). These data further confirm the enhanced Ca^2+^ permeability of the mutant channel.

To corroborate the above measurements, we directly assessed TPC2 channel activity in the lysosome by performing vacuolar patch clamp recordings. For these experiments, lysosomes were enlarged with vacuolin to facilitate analysis (Chen et al, 2017). We used the same recording solutions as for excised plasma membrane patches with Ca^2+^ as the major permeant ion in the pipette solution (corresponding to the lysosome lumen) and Na^+^ as the major permeant ion in the bath (corresponding to the cytosol). In vacuoles isolated from cells expressing wild-type TPC2, NAADP induced both inward Ca^2+^ currents and outward Na^+^ currents (Fig. 5I). Both were increased by the E215A mutation (Fig. 5I,J; Appendix Fig. S2a). PI(3,5)P_2_ induced robust outward Na^+^ currents and much more modest inward Ca^2+^ currents (Fig. 5K). There was a significant increase in the Ca^2+^ current upon mutation in vacuoles expressing TPC2^E215A^ in response to PI(3,5)P_2_ but little effect on the Na^+^ currents (Fig. 5K,L; Appendix Fig. S2b). Mutation of E215 did not alter E_rev_ for the NAADP-mediated current whereas it positively shifted it for PI(3,5)P_2_ (Appendix Fig. S2c,d). These data are entirely consistent with the plasma membrane electrophysiology (Fig. 2), confirming a gain-of-function in mutated TPC2 and enhanced Ca^2+^ permeability even to a normally Na^+^-linked agonist in a lysosomal setting.

**Figure EV5:**
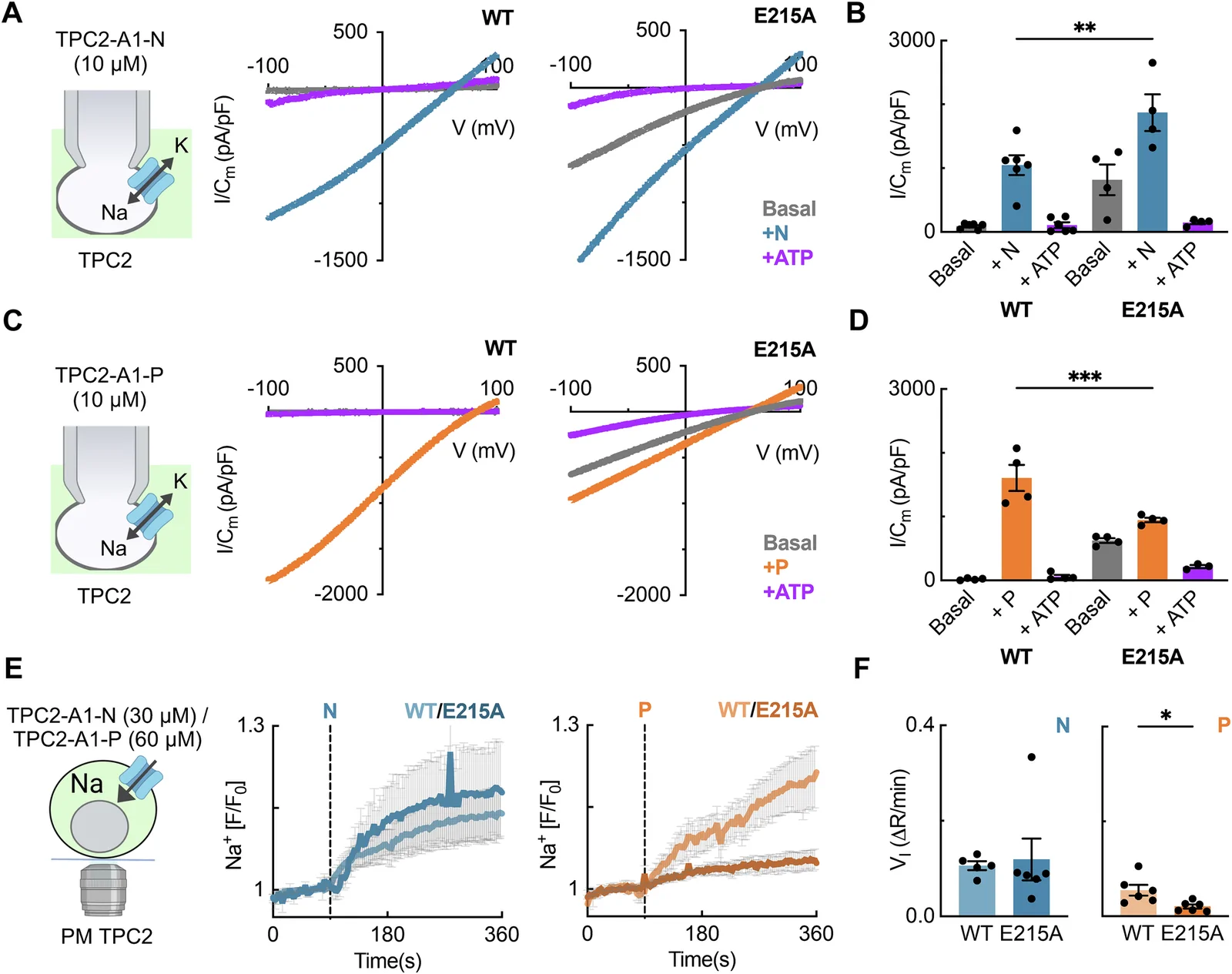
Effect of the E215A TPC2 mutation on Na^+^ currents and Na^+^ flux. (**A**–**D**) Vacuolar currents to TPC2 agonists recorded from HEK cells expressing wild-type or the E215A mutant in the lysosome using a Na^+^-rich luminal solution. Enlarged vacuoles were stimulated with TPC2-A1-N (**A**) or 10 µM TPC2-A1-P (**C**) followed by ATP (1 mM) using a pipette (luminal) solution containing 140 mM Na-MSA, 5 mM K-MSA, 2 mM Ca-MSA, 1 mM CaCl_2_, 10 mM HEPES and 10 mM MES (pH 4.6), and a bath (cytosolic) solution containing 140 mM K-MSA, 5 mM KOH, 4 NaCl mM, 0.39 mM CaCl_2_, 1 mM EGTA and 10 mM HEPES (pH 7.2). Exemplar recordings are shown. Pooled data (mean ± s.e.m.) quantifying the inward currents at −100 mV in response to TPC2-A1-N (b) or TPC2-A1-P (**D**). Data are from four to six independent experiments where each point represents the response of an individual patch. **P = 0.007, ***P = 0.001 (one-way ANOVA followed by Dunnett’s post hoc test). (**E**, **F**) HeLa cells expressing the indicated TPC2 construct were loaded with the Na^+^ indicator, ING-2 and stimulated with 30 µM TPC2-A1-N or 60 µM TPC2-A1-P in the presence of 2 mM extracellular Ca^2+^. Traces in e are pooled time course data (mean ± s.e.m.) where each point combines averages from five to six independent cell populations. Pooled data (mean ± s.e.m.) quantifying the initial rate of change of the ING-2 ratio from five to six independent experiments where each point represents the mean response from an individual population is shown in (**E**). *P = 0.02 (unpaired *t* test).

We performed an additional set of vacuolar patch-clamp experiments using more physiological solutions (high luminal Na^+^, high cytosolic K^+^). Interestingly, we noted a substantial increase in the basal inward (predominantly Na^+^) currents under these conditions (Fig. EV5A,B). Nevertheless, similar to the bi-ionic conditions, the E215A mutation increased currents to TPC2-A1-N but not to TPC2-A1-P (Fig. EV5A–D). Rather the latter appeared suppressed. A similar apparent reduction of the effects of TPC2-A1-P was obtained in Na^+^ imaging experiments using PM TPC2 (Fig. EV5E,F).

In sum, biased signaling through TPC2 is essential for lysosomal Ca^2+^ homeostasis.

### Deregulated agonist activation of TPC2 in vivo perturbs locomotion and viability of *C. elegans*

**Figure 6:**
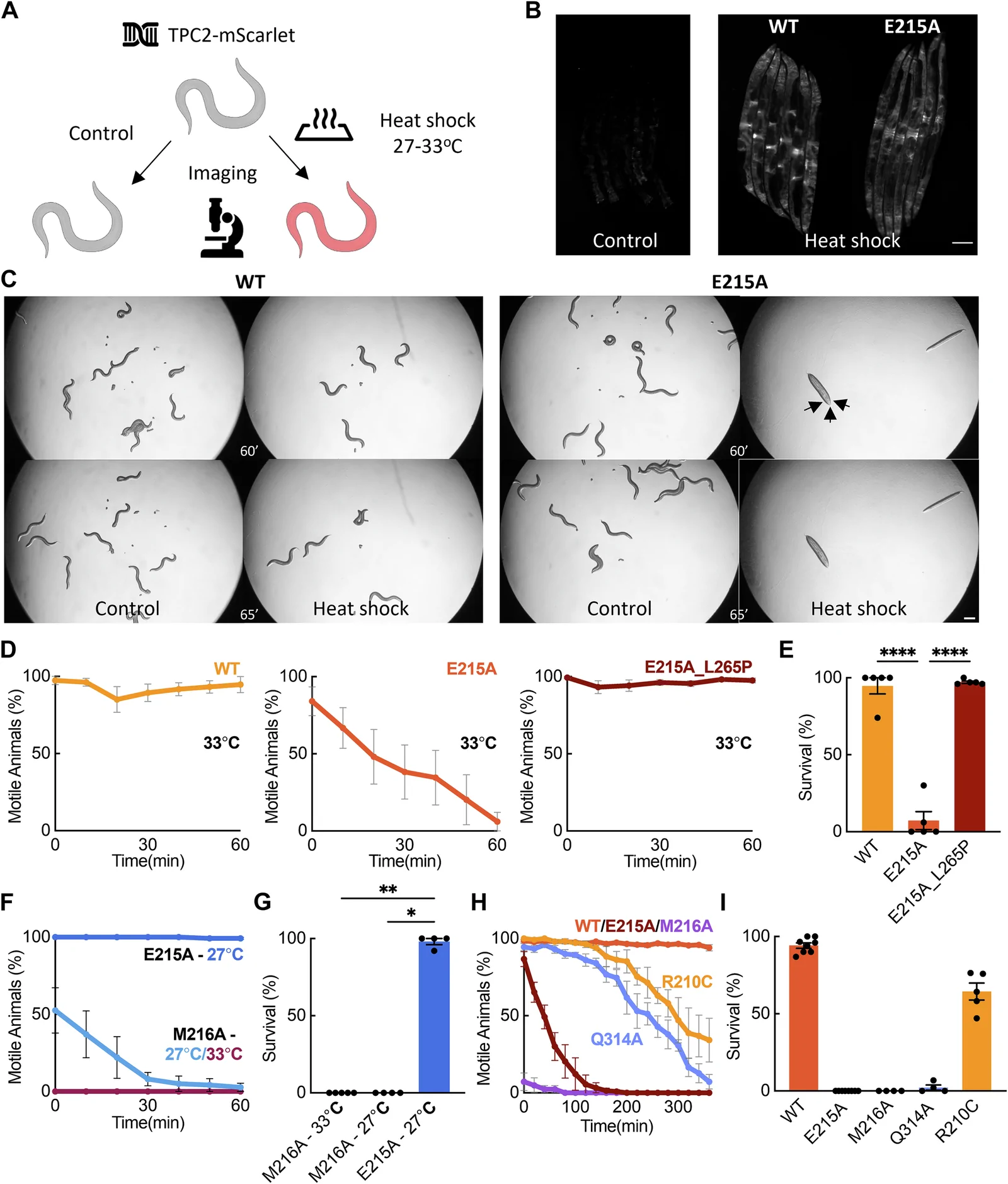
Deregulated agonist activation of TPC2 in vivo perturbs locomotion and viability of C. elegans. (**A**) Schematic showing generation of transgenic *C. elegans* expressing human TPC2 under the control of a heatshock promoter and the protocol used to observe behaviour upon protein induction. (**B**) Expression of human TPC2 and TPC2^E215A^ in transgenic *C. elegans*. Epifluorescence images of the mScarlet tag were acquired before or 60 min after heat shock (33 °C, 90 min). Results are representative of three experiments. Scale bar 100 µm. (**C**) Effect of TPC2^E215A^ on locomotion. Transmitted light images taken 5 min apart at the indicated times after heat shock at 33 °C was terminated. Arrows point to individual worms in a cluster. Results are representative of three experiments. Scale bar 500 µm. (**D**) Pooled data (mean ± s.e.m., *n* = 5) quantifying time-dependent effects of TPC2^E215A^ on motility upon protein induction. Data are compared to wild-type TPC2 and a double mutant (TPC2^E215A/L265P^) where the E215A mutation is combined with the pore-inactivating L265P mutation. (**E**) Effect of TPC2^E215A^ and TPC2^E215A/L265P^ on viability. Pooled data (mean ± s.e.m., *n* = 5) quantifying the effect of TPC2 on viability 24 h after protein induction where each point represents the results of an independent heat shock. ****P < 0.0001 (one-way ANOVA followed by Dunnett’s post hoc test). (**F**, **G**) Effect of TPC2^M216A^ on motility and viability. Pooled data (mean ±  s.e.m.) quantifying time-dependent effects of TPC2^M216A^ on motility upon protein induction (**F**). Pooled data (mean ±  s.e.m.) quantifying the effect of TPC2^M216A^ on viability 24 h after protein induction (**G**) where each point represents the results of an independent heat shock. Heat shock was performed at 33 °C and 27 °C as indicated. Data are from four to five experiments and compared to TPC2^E215A^. *P = 0.01, **P = 0.007 (Kruskal–Wallis test). (**H**, **I**) Effect of TPC2^Q314A^ and TPC2^R210C^ on motility and viability. Pooled data (mean ±  s.e.m.) quantifying time-dependent effects of the indicated mutant on motility upon protein induction (**H**). Pooled data (mean ± s.e.m.) quantifying the effect of TPC2 and the indicated mutant on viability 24 h after protein induction (**I**) where each point represents the results of an independent heat shock (33 °C). Data are from > four experiments and compared to wild-type TPC2, TPC2^E215A^ and TPC2^M216A^. Non-Unc-36, non-DpySte animals were selected for the motility/survival assays. Source data are available online for this figure.

To examine the functional consequences of perturbed agonist-regulation of TPC2, we generated transgenic roundworms (*Caenorhabditis elegans)* expressing wild-type and gain-of-function mutants. Like the fly, *Drosophila melanogaster*, this organism lacks TPC orthologues (Gunaratne et al, 2021), and thus can be considered a ‘natural knockout’. Their genetic tractability allows the properties of exogenously introduced TPC2 to be assessed in a genuine null background in vivo. TPC2 was tagged with mScarlet and placed under the control of a heat shock promoter (Fig. 6A). As shown in Fig. 6B, TPC2 expression was readily detectable in vivo after induction.

The locomotion activity of worms was monitored by time-lapse microscopy (Fig. 6C). Transgenic worms expressing wild-type TPC2 were motile throughout the observation period. Worms harboring TPC2^E215A^ were also motile immediately after heat shock. But motility was lost once protein expression was detectable (Fig. 6C). Paralysis for TPC2^E215A^ developed in a time-dependent manner post-heat shock, such that by 60 min, nearly all worms expressing mutant TPC2 were immobile (Fig. 6D; Movie EV2).

To determine whether paralysis was due to channel activity, we generated ’pore-dead’ animals by mutating L265 in the first pore-helix of TPC2. The resulting double transgenics (TPC2^E215A/L265P^) behaved similarly to the strain expressing wild-type TPC2 whereby heat shock had little effect on motility (Fig. 6D). Despite paralysis, worms expressing TPC2^E215A^ were viable following the observation period as evidenced by nose movements (Movie EV2). But after 24 h, all the mutant worms died following induction of TPC2 (Fig. 6E). Viability of the worms expressing wild-type TPC2 and the double mutant however was minimally compromised (Fig. 6E).

We leveraged the inducible nature of TPC2 expression in *C. elegans* to analyze the properties of the M216A mutant. This mutant did not express well in mammalian cells likely due to constitutive activity, elevated Ca^2+^ and cell death (Fig. EV1; Appendix Fig. S1). Transgenic worms carrying TPC2^M216A^ prior to induction appeared healthy and were motile (Appendix Fig. S3). Similar to the E215A mutant, the M216A mutant readily expressed upon induction and caused paralysis and subsequent death (Fig. 6F,G; Appendix Fig. S3). But notably, the paralyzing effect was immediately apparent following the heat shock period (Fig. 6F). To further characterize the functional effects of TPC2 mutants i*n vivo*, we reduced the temperature during heat shock (from 33 °C to 27 °C) to reduce protein expression. Under these conditions, the M216A mutant again reduced motility but its effects were slowed and developed progressively with time (Fig. 6E). In contrast, the effects of TPC2^E215A^ on motility and viability were both eliminated following induction at the lower temperature (Fig. 6E,F).

Finally, we compared the effects of the Q314A and R210C mutants. These mutants also reduced motility and viability (Fig. 6H,I). However, their effects were milder than the E215A and M216A mutants. Taken together, these data show that deregulated agonist action at TPC2 has significant time- and dose-dependent impact in vivo.

In sum, biased signaling through TPC2 is essential for organismal fitness.

## Discussion

Here we provide a molecular mechanism underpinning the unusual ability of an ion channel to switch its ionic profile upon ligand activation, and highlight the importance of this behaviour in maintaining lysosomal well-being.

Our previous work identified two surprising behaviors for TPC2. The first was the ability of TPC2 activators to switch the channel from a largely Na^+^-selective state upon activation with PI(3,5)P_2_ to a more Ca^2+^-permeable one upon activation with NAADP (Gerndt et al, 2020a). The second was the ability of these contrasting agonists to synergistically increase Ca^2+^ and only Ca^2+^ (not Na^+^) permeability when combined (Yuan et al, 2022). Here we link the two by showing that gain-of-function mutants effectively reduce both behaviors by converting the channel into a co-activated-like state that has negative impacts in vivo.

The discovery of cell-permeable TPC2 agonists has greatly facilitated analyses of TPC2. It was clear upon their identification that these mimetics had reciprocal effects on Na^+^ and Ca^2+^ permeability with TPC2-A1-P evoking larger Na^+^ currents than TPC2-A1-N and TPC2-A1-N evoking larger Ca^2+^ currents than TPC2-A1-P (Gerndt et al, 2020a). Our mutations go a long way in eliminating this highly unusual difference. It is noteworthy how the mutations similarly affected the synthetic agonist and its natural counterpart - perhaps most notably for TPC2-A1-N and NAADP given that NAADP binds not directly to TPC2 but instead to NAADP binding proteins that associate with the channel (Gunaratne et al, 2021; Gunaratne et al, 2023; Zhang et al, 2021). Agonist activation is decidedly complex especially when considering the significant overlap in molecular determinants. K204, for example, is central to PI(3,5)P_2_ binding and activation, but also contributes to activation by NAADP and TPC2-A1-P (in part), while being dispensable for TPC2-A1-N (Gerndt et al, 2020a; Saito et al, 2023; She et al, 2019; Yuan et al, 2022). An ‘intermediate’, PI(3,5)P_2_-driven conformational state has also recently been inferred drawing attention to the second voltage-sensing-like domain (Shimomura et al, 2023). What is important here, is that mutation of a single residue differentially affected the activity of all agonists such that TPC2 is converted into a ‘regular’ channel whereby both types of activators have more or less equivalent effects on permeability. This is worthy of remark given they normally have such contrasting effects.

It was also clear upon their identification, that TPC2-A1-N and TPC2-A1-P affected ion permeability just like NAADP and PI(3,5)P_2_ (Gerndt et al, 2020a). Selectivity filters are key to ion discrimination (Roux, 2017). And mutagenesis of these highly conserved structures has provided crucial insight into the fundamental workings of ion channels. Previous mutagenesis studies of the selectivity filter of TPC2 effectively converted it from a Na^+^- to a more Ca^2+^-permeable state upon PI(3,5)P_2_ activation by matching its sequence to that of plant TPC2, which is essentially non-selective to cations (Guo et al, 2017). The reported ~13-fold change in the relative permeability of Ca^2+^ to Na^+^ upon mutation is similar to the ~15-fold difference in relative permeability of TPC2 in the presence of NAADP and PI(3,5)P_2_. The E215A mutant reduced this difference down to ~5-fold when stimulated with PI(3,5)P_2_ or TPC2-A1-P. This was despite its positioning in the S4-S5 linker well away from the selectivity filter. This region thus appears critical not only for ligand activation but for tuning of ion permeability as well. Of note the equivalent linker in the second repeat appears disordered in the apo form but becomes ordered upon PI(3,5)P_2_ binding (She et al, 2019). This points to considerable plasticity, be it functional or structural, between the voltage-sensor and pore.

One of the most striking effects of the mutation was the substantial increase in the Ca^2+^ permeability of the channel upon stimulation with PI(3,5)P_2_ or its mimetic TPC2-A1-P. Although there was a corresponding increase in the reversal potential, the channel remained in a relatively Na^+^-selective state. This mismatch is highly reminiscent of the effect of co-stimulating TPC2 with its agonists (Yuan et al, 2022). Thus, the relative permeability of the mutant channel stimulated with PI(3,5)P_2_ or TPC2-A1-P (Fig. 2) matches that of the wild-type channel sequentially stimulated with PI(3,5)P_2_/NAADP or TPC2-A1-P/TPC2-A1-N, respectively (Yuan et al, 2022). Indeed, synthetic agonist combinations no longer mediate Ca^2+^ flux through the channel in a synergistic way. Rather, the agonists acted additively. This was true of both the E215A mutation in the linker and the Q314A mutation in the gate. Loss of agonist differentiation upon single stimulation appears closely linked to agonist integration upon co-stimulation.

Variations in the TPC2 gene are linked to pigmentation (Sulem et al, 2008). In particular, the M484L and G273E polymorphisms which associate with blonde hair are both gain-of-function (Chao et al, 2017). The Ca^2+^ permeability of these mutants has not been measured but the M484L mutant increased maximal Na^+^ currents and channel sensitivity to PI(3,5)P_2_ thereby distinguishing it from the E215A mutant (Chao et al, 2017). Indeed, TPC2-A1-P and TPC2-A1-N had differential effects on Na^+^ permeability similar to their effects on the wild-type channel (Gerndt et al, 2020a). Recent studies have identified a pathogenic variant (R210C) underlying hypopigmentation (Wang et al, 2023). Again the Ca^2+^ permeability of the mutant was not directly measured but there was a marked leftward shift in the concentration-effect relationships for PI(3,5)P_2_-mediated Na^+^ currents similar to the M484L variant although whether the Na^+^ currents were affected upon maximal stimulation was unclear. Our Ca^2+^ measurements confirm the gain-of-function effects of R210C and also its reported constitutive activity. Considerable constitutive activity was also noted for the M216A mutant. Importantly, pathogenic TPC2 lost its ability to be activated synergistically by its ligands. These data thus link plastic flux to disease.

Ca^2+^ signals recorded in cells expressing the E215A in the lysosome were notably sustained and thus indicative of Ca^2+^ entry (Fig. 5). This could result from  insertion of TPC2 channels into the plasma membrane as shown for the R210C mutation (Wang et al, 2023), perhaps through lysosomal exocytosis. Thus, defective Ca^2+^ fluxes might derive not only from the lysosome but also from the cell exterior. This could be functionally relevant and most likely contribute to the locomotion defects in vivo reported here that ultimately result in death of *C. elegans* (Fig. 6). This was particularly striking for the M216A mutant. Of relevance are exaggerated Ca^2+^ fluxes through TPCs upon loss of OCaR1, a novel negative regulator of TPC activity (Tsvilovskyy et al, 2024). This was linked to hyper-secretion (increased exocytosis of zymogen granules from pancreatic acinar cells) and disease (exacerbated phenotypes in pancreatitis models). Beyond TPCs, TRPML3 is a distinct lysosomal ion channel mutated in the varitint-waddler mouse (Grimm et al, 2007; Kim et al, 2007; Xu et al, 2007). This mouse has striking coloration and is deaf due to death of hair cells in the inner ear. This is thought to result from a gain-of-function in TRPML3 (Atiba-Davies and Noben-Trauth, 2007), further underscoring the importance of keeping lysosomal Ca^2+^ signals in check.

Overall, we suggest that strict agonist-dependent regulation of TPC2 is crucial to lysosomal homeostasis, and that its deregulation through molecular variation (whether experimental or through disease) has negative impacts. Such tempering of ionic fluxes through TPC2 thus emerges as an essential organellar safeguarding mechanism that could potentially be tapped to promote well-being.

## Methods

**Table:** Reagents and tools table

Reagent/resource	Reference or source	Identifier or catalog number
**Experimental models**		
HeLa cells	ATCC	CCL-2
HEK-293T cells	ATCC	CRL-3216
U2OS cells	ATCC	HTB-96
HEK-293 cells	DSMZ	ACC 305
*C. elegans*	This study	Appendix Table S2
**Recombinant DNA**		
TPC2 expression plasmids	This study	Methods
**Antibodies**		
Mouse anti-GFP	SantaCruz	sc-9996
Mouse anti-RFP	Proteintech	6g6
Rabbit anti-beta actin	Proteintech	20536-1-AP
IRDye® 800CW donkey anti-mouse IgG	LiCor	926-32212
IRDye® 680RD Donkey anti-rabbit IgG	LiCor	926-68073
**Oligonucleotides and other sequence-based reagents**		
Mutagenic primers	This study	Appendix Table S1
**Chemicals, enzymes and other reagents**		
Poly-L-lysine	Sigma	P6282
DMEM	Thermo Fisher	31966-021
Fetal Bovine Serum	Thermo Fisher	10500064
Penicillin-Streptomycin	Thermo Fisher	15140
Trypsin-EDTA	Thermo Fisher	25200056
Lipofectamine^TM^ 2000	Invitrogen	11668027
TurboFect	Thermo Fisher	R0531
TurboFectin 8.0	OriGene	TF81001
Phosphate buffered saline	Sigma	P4417
Tris-buffered saline	Merck	524750
Hanks’ balanced salt solution	Corning	21-023-CV
Triton X-100	Roche	10789704001
Halt™ inhibitor cocktail	Thermo Fisher	78441
Bicinchoninic acid	Sigma	B9643
Bovine serum albumin	Sigma	A7906
NuPAGE Protein Gels	Invitrogen	NP0321
NuPAGE™ Running Buffer	Invitrogen	NP0001
Fura-2 AM (HeLa)	Biotium	50033
Fura-2 AM (U2OS)	Invitrogen	F1221
Fura-FF AM	Cayman Chemical	20416
ING-2 AM	ION Biosciences	2011
Pluronic F-127	Biotium	BT59004
Paraformaldehyde	Thermo Fisher	J19943-K2
Fluoromount-G	SouthernBiotech	0100-01
TWEEN 20	Sigma	P7949
DAPI	Roche	10236276001
Nystatin, 85 + %	Thermo Scientific	455500250
Streptomycin Sulfate	Fisher	15424871
Agarose	Invitrogen	16500
PEG 8000	Sigma	P2139
Cholesterol	Sigma	C8503
M9 salt	Serva	48505.01
Apilimod	Sigma	SML2974
NAADP (macropatches)	MedChemExpress	HY-103317A
NAADP (microinjection)	Sigma	N5655
NAADP (vacuoles)	Tocris Bioscience	3905
PI(3,5)P_2_ (macropatches)	Echelon	P-3508
PI(3,5)P_2_ (microinjection)	Cayman Chemical	10008398
PI(3,5)P_2_ (vacuoles)	Avanti Research	850184 P
TPC2-A1-N and TPC2-A1-P	Synthesized as described in (Gerndt et al, 2020a)	
**Software**		
Image Studio Lite 5.2	LICOR	
MetaFluor 7.10.3.279	Molecular Devices	
NIS-Elements AR 3.1	Nikon	
pClamp 10	Molecular Devices	
PatchMaster v2x90.4	HEKA	
Fiji 2.16.0	Opensource	
Mars 3.43 R3	Molecular Devices	
AlphaFold2	Jumper et al, 2021	
PROPKA 3	Olsson et al, 2011	
GROMACS 2021	Abraham et al, 2015	
MDAnalysis package	https://www.mdanalysis.org/	
ZEN 2.6	Zeiss	
VMD	Humphrey et al, 1996	
CHARMM-GUI Membrane builder	Wu et al, 2014	
**Other**		
FemtotipsII	Eppendorf	EP5242957000

### Plasmids

For expression in mammalian cells, plasmids were based on TPC2^L11A/L12A^-GFP (Brailoiu et al, 2010), referred to here as PM TPC2, or TPC2 mRFP (Brailoiu et al, 2009) in pCS2 + . Additional mutations were introduced by site-directed mutagenesis using the primers listed in Appendix Table S1 or commercially by Genscript (Oxford, UK) in the case of the S218A, S313A and R210C mutants.

For expression in *C. elegans*, TPC2, TPC2^E215A^ or TPC2^M216A^ restriction digest fragments were cloned into pHygG3 (Nonet, 2023) along with the *hsp-16.2* promoter, *C. elegans* codon-optimized, intron-containing mScarlet, and the *tbb-2* 3’UTR. TPC2^E215A/L265P^ was generated commercially using the corresponding TPC2^E215A^ plasmid, and TPC2^Q314A^ and TPC2^R210C^ from the corresponding wild-type plasmid.

### Cell lines

HeLa cells, HEK-293T cells (up to passage 20), U2OS cells and HEK-293 cells were maintained in Dulbecco’s Modified Eagle Medium (DMEM), supplemented with 10% Fetal Bovine Serum (FBS), 100 μg/mL streptomycin and 100 units/mL penicillin (all from Invitrogen) at 37 °C in a humidified atmosphere with 5% CO_2_. Cells were passaged with trypsin and routinely tested for mycoplasma. For experimentation, cells were plated onto six-well plates for western blotting, coverslips coated with poly-L-lysine for imaging (20 μg/mL), cell surface electrophysiology (1 mg/mL) and lysosomal electrophysiology (100 μg/mL), and opaque-walled 96 well microplates for cell population measurements. For lysosomal electrophysiology, cells were treated with apilimod (1 µM) for 14 h to 18 h to enlarge endo-lysosomal organelles.

### *C. elegans* strains

TPC2 plasmids in pHygG3 were used to generate single copy integrant lines as described by (Nonet, 2023). Standard genetic methods were used to combine integrated transgenes with *aeaIs10*. All *C. elegans* stocks are listed in Appendix Table S2. They were maintained at 20 °C on nematode growth medium (Brenner, 1974) supplemented with 10 µg/ml nystatin and 50 µg/ml streptomycin, *using E. coli* AMA1004 (Casadaban et al, 1983) as the food source.

### Transfection

HeLa and U2OS cells used for imaging were transfected with Lipofectamine^TM^ 2000 or TurboFectin 8.0, respectively 18–26 h prior to analysis. HEK-293T cells used for cell surface electrophysiology were transfected with Lipofectamine^TM^ 2000 24–36 h prior to recording. HEK-293 cells used for vacuolar patch-clamp experiments were transfected with TurboFect^TM^ 36–48 h prior to recording.

### Western blotting

HeLa cells were harvested with trypsin-EDTA, washed once with phosphate-buffered saline by centrifugation and lysed in phosphate-buffered saline containing 1% Triton X-100 and Halt™ protease and phosphatase inhibitor cocktail for 30 min at 4 °C with rotation. The supernatant was collected after centrifugation (16,000 × *g* 10 min, 4 °C). The protein concentrations were measured using bicinchoninic acid and bovine serum albumin protein standards. The cell lysates (30 μg) were separated on NuPAGE 4–12% Bis-Tris Protein Gels and transferred to nitrocellulose membranes according to manufacturer’s instructions (XCell II^TM^ Blot Module, Invitrogen). The membranes were then blocked with 5% w/v dried skimmed milk in Tris-buffered saline containing 0.1% v/v Tween 20 (TBS-T) for 1 h at room temperature. Blots were sequentially incubated with primary and secondary antibodies in TBS-T supplemented with 2.5% (w/v) dried skimmed milk. The blot was cut above 50 kDa after blocking. The primary antibodies used were mouse anti-GFP (1 in 1000 dilution) or mouse anti-RFP (1 in 2000 dilution) for the top part (overnight 4 °C) and mouse anti-beta actin for the bottom part (1 in 5000 dilution, 1 h room temperature).

Secondary antibody used was IRDye 800CW donkey anti-mouse IgG (1 in 14,000 dilution, for 1 h room temperature). The fluorescence was detected using Odyssey CLx (LiCor) and images were analysed using Image Studio Lite version 5.2.

### Single cell cytosolic Ca^2+^ measurements

Cytosolic Ca^2+^ in individual HeLa and U2OS cells was measured using the fluorescent Ca^2+^ indicator, Fura-2.

For HeLa cells, Ca^2+^ imaging experiments were performed at room temperature in HEPES-buffered saline (HBS) containing 10 mM NaHEPES, 1.25 mM KH_2_PO_4_, 2 mM MgSO_4_, 3 mM KCl, 156 mM NaCl, 2 mM CaCl_2_ and 10 mM glucose (pH 7.4). Cells were incubated with Fura-2 AM (2.5 µM) and 0.005% (v/v) pluronic acid for 1 h in HBS. Cells were washed in HBS and were subsequently mounted in a 1 mL imaging chamber (Biosciences Tools) for microscopy. Epifluorescence images were acquired every 3 s using a Megapixel monochrome cooled coupled device camera attached to an Olympus IX73 inverted fluorescence microscope fitted with a CoolLED multiple wavelength LED source under the control of MetaFluor 7.10.3.279 software. Fura-2 was excited at 340/380 nm and emitted fluorescence was captured using a 425 nm long-pass filter with a ×20 objective. Cells were stimulated with TPC2-A1-N and TPC2-A1-P by bath additions.

For U2OS cells, Ca^2+^ imaging experiments were performed at room temperature in Hanks’ balanced salt solution (HBSS, Corning, Mediatech, Inc, Manassas, VA). Cells were incubated with Fura-2 AM (5 µM, Invitrogen) for 45 min, washed with dye-free HBSS, and incubated for an additional 45 min to allow dye de-esterification. Coverslips were mounted in an open bath chamber (RP-40LP, Warner Instruments, Hamden, CT) for microscopy. Epifluorescence images were acquired every 4 s using a Photometrics CoolSnap HQ2 CCD camera (Photometrics, Tucson, AZ) attached to an inverted microscope (Nikon Eclipse TiE, Nikon Inc., Melville, NY) fitted with a Perfect Focus System under the control of NIS-Elements AR version 3.1 (Nikon Inc.). Fura-2 was excited at 340/380 nm and emitted fluorescence was captured at 510 nm with a ×40 objective. Cells were stimulated with NAADP and PI(3,5)P_2_ by intracellular microinjection.

### Single cell cytosolic Na^+^ measurements

Cytosolic Na^+^ in HeLa cells was measured using the fluorescent Na^+^ indicator ING-2. Na^+^ imaging experiments were performed at room temperature in HBS. Cells were incubated with ING-2 AM (5 µg/ml) and 0.005% (v/v) pluronic acid for 1 h in HBS. Cells were washed in HBS and were subsequently mounted in a 1 mL imaging chamber (Biosciences Tools) for microscopy. Epifluorescence images were acquired every 3 s. Images were captured with a cooled coupled device camera (TILL photonics) attached to an Olympus IX71 inverted fluorescence microscope fitted with a monochromatic light source under the control of TillVision 4.0 software. ING-2 was excited at 488 nm and emitted fluorescence was captured using a 515 nm long-pass filter with a ×20 objective. Cells were stimulated with TPC2-A1-N and TPC2-A1-P by bath additions.

### Intracellular microinjection

Intracellular microinjections were performed using FemtotipsII, InjectManNI2 and FemtoJet systems (Eppendorf). Pipettes were back-filled with an intracellular solution containing, in mM: 110 KCl, 10 NaCl and 20 HEPES (pH 7.2) and supplemented with either NAADP (250 nM) or P(3,5)P_2_ (25 µM). The injection time was 0.4 s at 60 hPa with a compensation pressure of 20 hPa in order to maintain the microinjected volume to less than 1% of cell volume, as measured by microinjection of a fluorophore (Fura-2 free acid). The intracellular concentration was estimated based on the concentration in the pipette and the volume of injection. The cells to be injected were Z-scanned before injection and the cellular volume automatically calculated by the NIS-Elements AR 3.1 software (Nikon, Inc.).

### Confocal and TIRF microscopy

Cells were fixed with 4% w/v PFA in phosphate-buffered saline (PBS) for 10 min at room temperature. Coverslips were washed thrice with PBS, incubated with 1 µg/mL DAPI in PBS supplemented with 0.1% v/v Tween-20 (PBS-T) for 5 min and wash thrice with PBS-T prior to mounting on microscope slides using Fluoromount-G.

Confocal images were captured using a Zeiss 880 axio observer Z1, fitted with Plan-Apochromat ×63/1.4 Oil DIC objective. DAPI, GFP and mRFP were excited using laser lines of 405 nm, 488 nm and 561 nm, respectively. Emitted fluorescence was captured using 410–479 nm, 490–577 nm, or 578–696 nm bandpass filters, respectively. Confocal images were analysed using Fiji software.

TIRF images were captured using a Zeiss ELYRA7 (Carl Zeiss, AG) fitted with a ×63 oil immersion plan apochromat lens (N.A. 1.46). GFP was excited using a 488 nm laser line at an angle of 69° to achieve TIRF with a penetration depth of ~93 nm.

### Electrophysiology

Agonistevoked currents were recorded through TPC2 expressed at the cell surface or within enlarged lysosomes of HEK cells.

Currents at the cell surface were recorded in the inside-out configuration from macropatches excised from the plasma membrane of HEK-293T cells transiently expressing TPC2. Data were acquired using an AxoPatch 200 B amplifier (Molecular Devices) and pClamp10.2 suite (Molecular Devices). Records were filtered at 2 kHz and digitized at 10 kHz using Digidata 1440 A (Molecular Devices). ClampFit 10.2 was used for offline analysis of data. Currents were evoked by voltage ramps from −100 to +100 mV over 400 ms repeated at 5 s intervals from a holding potential of 0 mV.

Patch-pipettes were pulled from thick-walled, filamented borosilicate glass capillaries (Shutter Instrument) using Narishige PC-10 vertical puller, fire polished using a Narishige MF-830 microforge (Digitimer Ltd.). Agonists were applied to the bath solution of excised macropatches via an 8-channel pressurized perfusion system controlled by ValveLink 8.2 controller (AutoMate Scientific).

Currents at the lysosomes were recorded in the whole-vacuole configuration from enlarged lysosomes manually excised from HEK-293 cells transiently expressing TPC2 as described in (Chen et al). Data were acquired, digitized (40 kHz) and filtered (2.9 kHz) using an EPC-10 amplifier and PatchMaster software v2x90.4 (both HEKA, Lambrecht, Germany). During each recording fast and slow capacitive transients were cancelled by amplifier compensation circuit. Currents were evoked by voltage ramps from −100 to +100 mV repeated at 5 s intervals from a holding potential of 0 mV and normalized to organelle size.

Patch pipettes were pulled from borosilicate glass and polished to resistances in the range of 8–11 MΩ. Liquid junction potential was corrected as described (Chen et al). Agonists were applied by complete exchange of the cytoplasmic solution. All compounds were freshly diluted before experimentation.

For bi-ionic measurements, the pipette (luminal) solution contained (in mM) 105 CaCl_2_, 5 HEPES and 5 MES (pH adjusted to 4.6 using MSA) and the bath (cytosolic) solution 160 NaCl and 5 HEPES (pH adjusted to 7.2 using NaOH).

The permeability ratio (P_Ca_/P_Na_) was calculated from the reversal potential according to (Fatt and Ginsborg, 1958):

$$\frac{{P}_{{Ca}}}{{P}_{{Na}}}=\frac{{\gamma }_{{Na}}}{{\gamma }_{{Ca}}}* \frac{{\left[{Na}\right]}_{i}}{{4\left[{Ca}\right]}_{o}}* {exp }^{\left({E}_{{rev}}\frac{F}{{RT}}\right)}* ({exp }^{\left({E}_{{rev}}\frac{F}{{RT}}\right)}+1)$$

where *P*_*Ca*_ = Ca^2+^ permeability; *P*_*Na*_ = Na^+^ permeability; *γ*_*Ca*_ = Ca^2+^ activity coefficient (0.52); *γ*_*Na*_ = Na^+^ activity coefficient (0.75); [Ca]_o_ = concentration of Ca^2+^ in the lumen; [Na]_i_ = concentration of Na^+^ in the cytosol; *E*_*rev*_ = reversal potential; F – Faraday’s constant, R – gas constant; T – absolute temperature.

For Na^+^ permeability measurements, the pipette (luminal) solution contained (in mM) 140 Na-MSA, 5 K-MSA, 2 Ca-MSA, 1 CaCl_2_, 10 HEPES and 10 MES (pH was adjusted to 4.6 with MSA), and the bath (cytosolic) solution contained 140 K-MSA, 5 KOH, 4 NaCl, 0.39 CaCl_2_, 1 EGTA and 10 HEPES (pH was adjusted with KOH to 7.2).

All electrophysiological recordings were made at room temperature (21–23 °C).

### Population-based cytosolic Ca^2+^ measurements

Cytosolic Ca^2+^ in populations of HeLa cells transiently expressing PM TPC2 was measured using Fura-FF and a fluorescence plate reader (Clariostar, BMG Labtech) under the control of Mars 3.42 R3 software. Cells were incubated with Fura-FF (2.5 µM) and 0.005% (v/v) pluronic acid (from Invitrogen) for 1 h in HBS. A single measurement comprised 16 flashes at 335 nm and 380 nm (each at 8 nm bandpass) while recording fluorescence at 520 nm (90 nm bandpass). Measurements were repeated on an individual well at 60 s intervals with 15 wells being recorded in parallel using “plate mode”. Defined volumes of TPC2-A1-N and TPC2-A1-P were added simultaneously through two independent injector needles to achieve the indicated final concentrations. Background fluorescence was measured from wells containing cells that were incubated with HBS without Fura-FF.

### Molecular dynamics simulations

Human TPC2 with PI(3,5)P_2_-bound in an open state (PDB ID: 6NQ0) (She et al, 2019) was used as the starting point for MD simulations. The unresolved C-terminal region was built using AlphaFold2 (Jumper et al, 2021). The pKa values were calculated using PROPKA3.0 (Olsson et al, 2011), which predicted standard protonation states. The CHARMM-GUI Membrane Builder (Lee et al, 2016; Wu et al, 2014) was used to prepare the systems, whereby the protein was embedded in a POPC bilayer, solvated and 0.15 M NaCl added to neutralize the system (Huang et al, 2017). The CHARMM36m force field (Huang et al, 2017) was used for the protein and the TIP3P model was employed for the water. The CHARMM-GUI protocol (Lee et al, 2016; Wu et al, 2014) was adopted for system equilibration and semi-isotropic periodic boundary conditions were applied. The hydrogen-containing bonds were constrained using the LINCS algorithm. The time-step was 2 fs and the leap-frog algorithm was selected for integration of the equations of motion. Coordinates were saved every 100 ps. Particle-mesh Ewald (PME) summation was used for electrostatic interactions with a cubic interpolation and a grid spacing of 0.12 nm. The van der Waals interactions were smoothly switched off from 1.0 nm to 1.2 nm. The temperature was maintained at 310 K with the v-rescale thermostat (Bussi et al, 2007) and a coupling constant of 0.5 ps, whereas the target pressure was maintained at 1 bar with the Parrinello-Rahman barostat and a coupling constant of 5 ps. Three repeats of 1 μs were obtained using GROMACS version 2021 (Abraham et al, 2015). The analysis was performed with GROMACS tools and in-house scripts using the MDAnalysis package (https://www.mdanalysis.org/). VMD was used for visualization (Humphrey et al, 1996).

### Locomotion assays

To induce expression of *Phsp-16.2::TPC2::mScarlet* or the mutants in *C. elegans*, ~25–40 one-day-old hermaphrodites that had been raised at 20 °C were placed on 0.5 cm-thick agarose plates (2% Agarose, 5% v/v LB media, 0.1% PEG 8000, 0.8 µg/ml cholesterol in M9) (Brenner, 1974) and incubated at 27–33 °C for 90 min. After heat-shock, animals were followed during recovery at 20 °C. They were inspected at 10-minute intervals for the first hour, and then at 24 h to observe changes in phenotype. Animals were counted as paralysed if they did not respond when mechanically stimulated by repeated lifting and dropping of one side of the plate.

### In vivo imaging

Expression of TPC2-mScarlet was determined in *C. elegans* immobilised using 20 mM Levamisole. Images were captured using a Zeiss Axio Imager.M2 fitted with a Zeiss 10x A-Plan objective using Prime BSI camera connected to a PC running ZEN (2.6) software. mScarlet fluorescence was excited using a Colibri 7 light source (592/25 nm) and imaged with Zeiss filter set 90. Images were analysed and processed using Fiji software. Each image was converted to 8-bit format then background signal was measured and subtracted. Brightness was then increased by multiplying each image by 1.5.

### Analysis

All experiments were non-blinded and performed at least three times. Parametric tests were performed using an unpaired *t* test or one-way ANOVA. Non-parametric tests were performed using Kruskal–Wallis. All data were analyzed using Prism 9 (GraphPad Software) where *n* refers to the number independent experiments. *P < 0.05, **P < 0.01, ***P < 0.001, ****P < 0.0001. Where multiple mutant TPC2 constructs were analyzed in parallel within an individual experiment, common wild-type controls were used. Consequently, some of the corresponding wild-type control datasets are shared across the relevant figures.

## Supplementary information

Appendix

Peer Review File

Movie EV1

Movie EV2

Source data Fig. 1

Source data Fig. 2

Source data Fig. 3

Source data Fig. 4

Source data Fig. 5

Source data Fig. 6

Expanded View Figures

## Data Availability

Source data are provided with this paper. The source data of this paper are collected in the following database record: biostudies:S-SCDT-10_1038-S44318-026-00882-1.
