# Supplementary material for: Allosteric switching of Ca2+ permeability in the lysosomal ion channel TPC2 underpins biased agonist activation
Source: EMBO J. 2026 Aug 7;45(18):6501–26. doi: 10.1038/s44318-026-00882-1 (PMC13578460; doi:10.1038/s44318-026-00882-1)
Supplement: Supplementary file 1 [file 44318_2026_882_MOESM11_ESM.pdf]

Expanded View Figures

Figure EV1. Expression analysis of recombinant TPC2.

(**A–C**) HeLa cells were transiently transfected with GFP-tagged TPC2$^{L11A/L12A}$ (PM TPC2) or mRFP-tagged TPC2 (TPC2) with and without (WT) the additional indicated mutation. Western blot analyses of cell lysates expressing TPC2 using an antibody to GFP, mRFP or β-actin (**A**). Data are representative of three experiments. Fluorescence intensity (FI) analysis of individual cells expressing TPC2 (**B**). Pooled data (mean ± s.e.m.) quantifying GFP or mRFP fluorescence from 3 to 23 independent experiments where each point represents the mean intensity from an individual population. Confocal analyses of PM (green) and lysosomal (red)-targeted TPC2 constructs (**C**). Nuclei were stained with DAPI (blue). Scale bar 20 μm. Data are representative of three experiments.

© The Author(s)

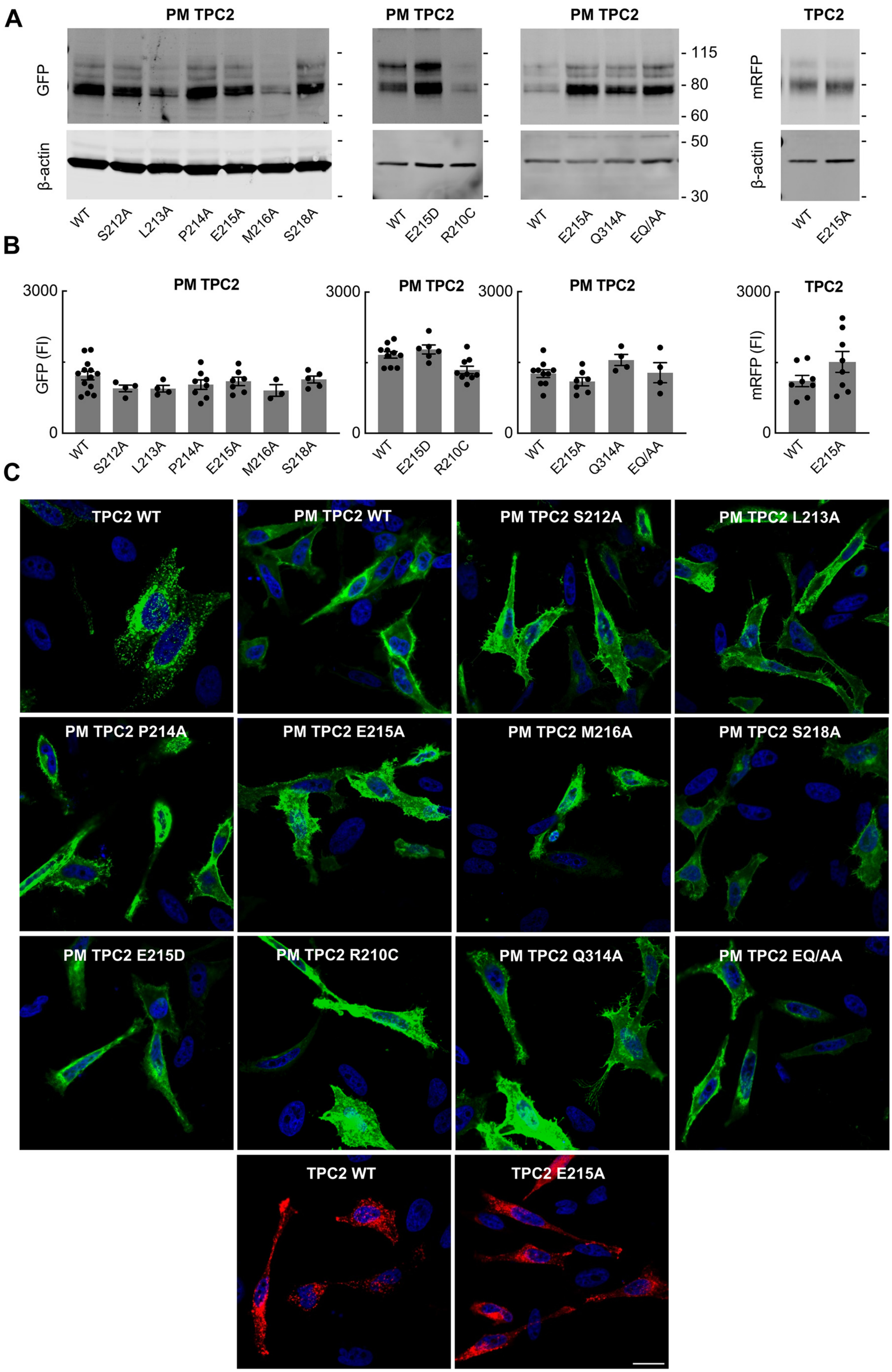
A
PM TPC2
PM TPC2
PM TPC2
TPC2
GFP
β-actin
mRFP
β-actin
- 115
- 80
- 60
- 50
- 30
WT S212A L213A P214A E215A M216A S218A
WT E215D R210C
WT E215A Q314A EQ/AA
WT E215A
B
PM TPC2
PM TPC2
PM TPC2
TPC2
3000
0
GFP (FI)
mRFP (FI)
WT S212A L213A P214A E215A M216A S218A
WT E215D R210C
WT E215A Q314A EQ/AA
WT E215A
C
TPC2 WT
PM TPC2 WT
PM TPC2 S212A
PM TPC2 L213A
PM TPC2 P214A
PM TPC2 E215A
PM TPC2 M216A
PM TPC2 S218A
PM TPC2 E215D
PM TPC2 R210C
PM TPC2 Q314A
PM TPC2 EQ/AA
TPC2 WT
TPC2 E215A

 © The Author(s)

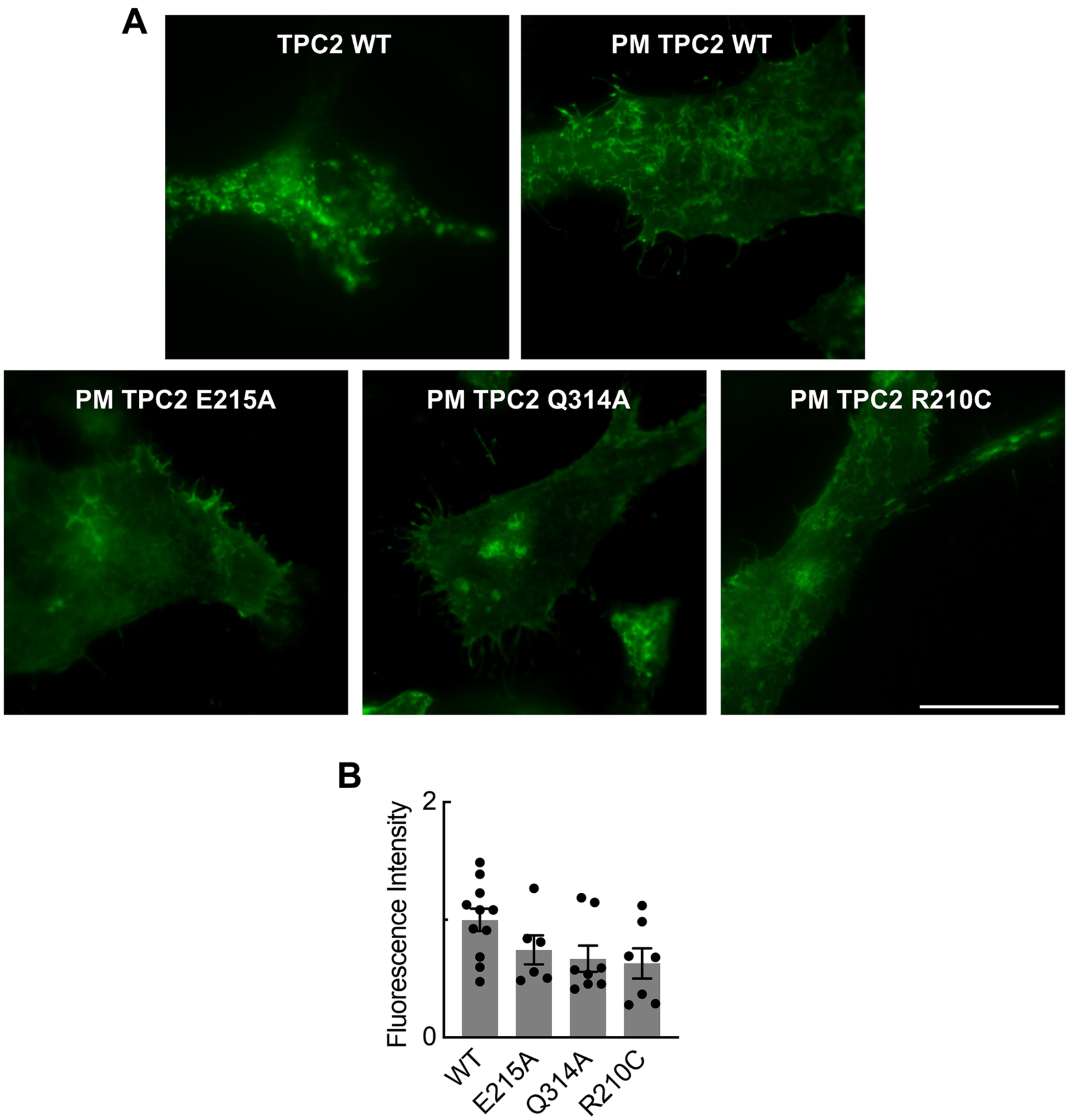

Figure EV2. Expression analysis of plasma membrane-targeted TPC2.

(**A**) Exemplar TIRF images of the indicated GFP-tagged TPC2 constructs expressed in HeLa cells. Scale bar 20 µm. (**B**) Pooled data (mean ± s.e.m.) quantifying fluorescence from two independent transfections where each point represents the intensity from an individual cell expressing the indicated plasma membrane-targeted mutant. Data are normalised to wild-type PM TPC2.

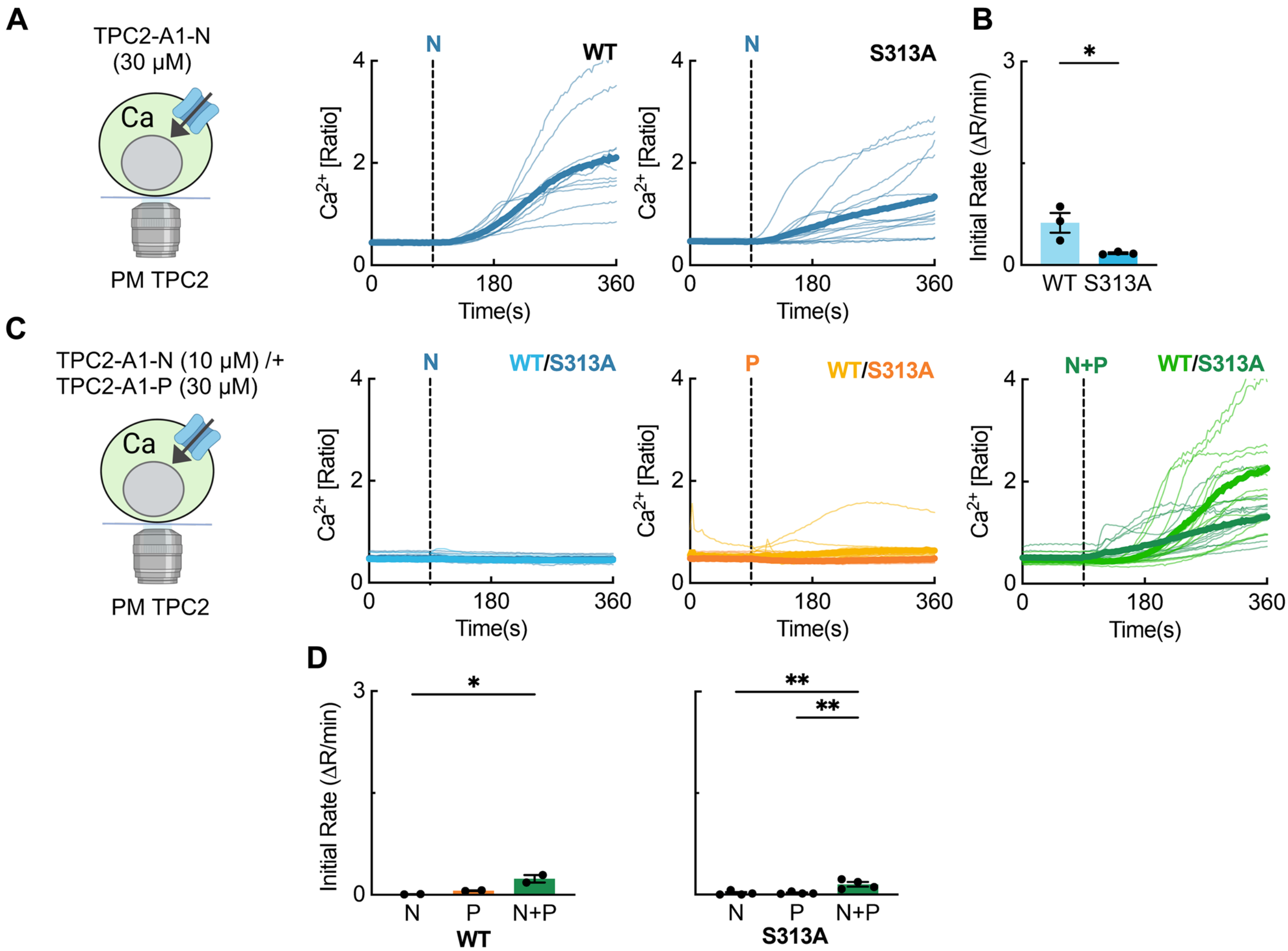

Figure EV3. Effect of the S213A TPC2 mutation on Ca^{2+} flux.

(**A**) HeLa cells expressing the indicated TPC2 construct were loaded with the ratiometric Ca^{2+} indicator, Fura-2 and stimulated with 30 μM TPC2-A1-N in the presence of 2 mM extracellular Ca^{2+}. Each trace is the fluorescence ratio imaged from a single GFP-positive cell from a typical field of view. The thicker trace is the average of the population. (**B**) Pooled data (mean ± s.e.m.) quantifying the initial rate of change of the Fura-2 ratio from three independent experiments where each point represents the mean response from an individual population. *$P = 0.04$ (unpaired *t* test). (**C**) HeLa cells expressing the indicated TPC2 construct were loaded with the ratiometric Ca^{2+} indicator, Fura-2 and stimulated with 10 μM TPC2-A1-N, 30 μM TPC2-A1-P or a combination of the two in the presence of 2 mM extracellular Ca^{2+}. Each trace is the fluorescence ratio imaged from a single GFP-positive cell from a typical field of view. The thicker trace is the average of the population. (**D**) Pooled data (mean ± s.e.m.) quantifying the initial rate of change of the Fura-2 ratio from four independent experiments where each point represents the mean response from an individual population. *$P = 0.03$, **$P = 0.006$ (one-way ANOVA followed by Dunnett's post hoc test).

 © The Author(s)

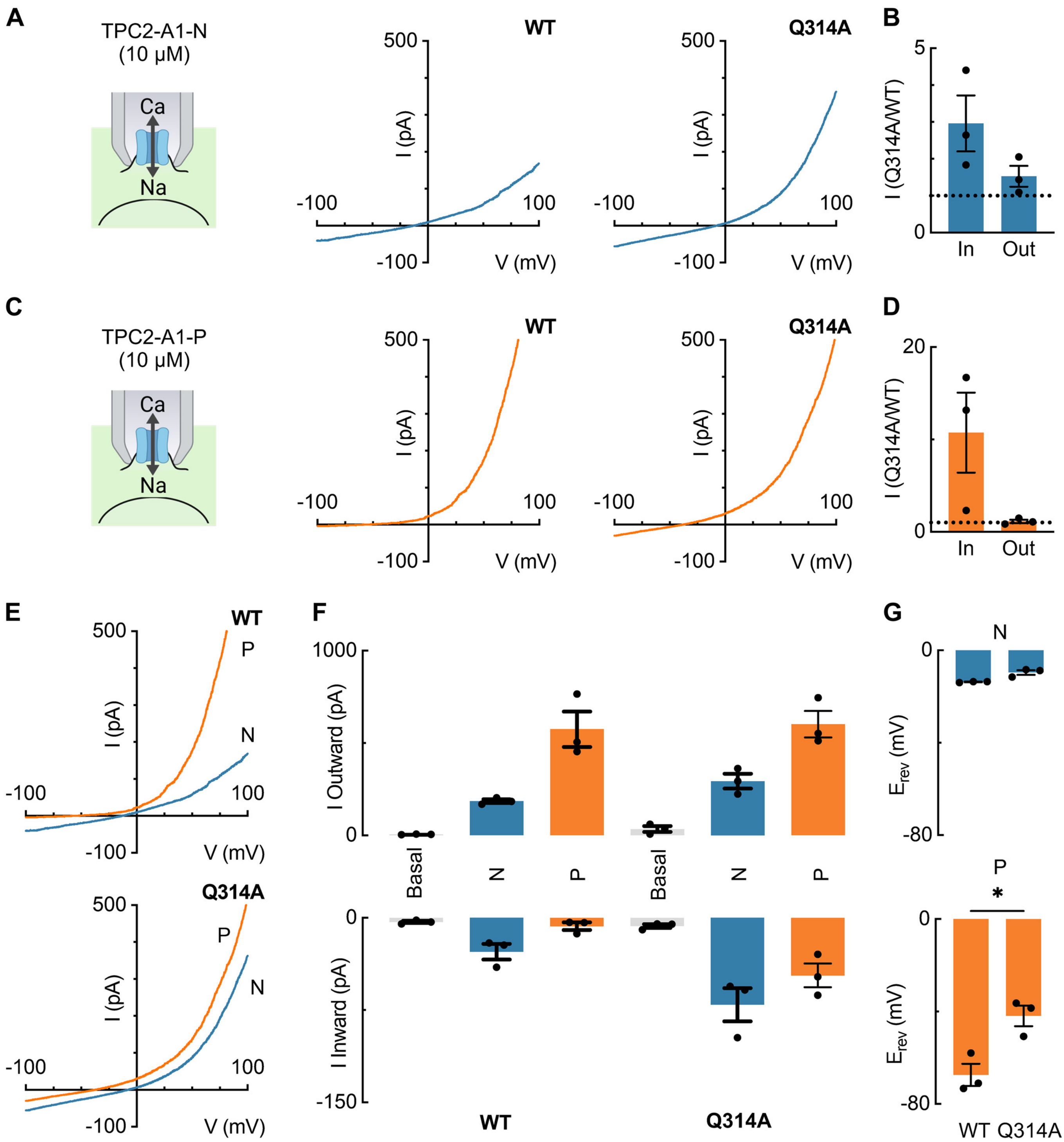

Figure EV4. Effect of the Q314A TPC2 mutation on Na^+ and Ca^{2+} currents.

(**A–D**) Macropatches from HEK cells expressing the indicated TPC2 construct were stimulated with 10 μM TPC2-A1-N (**A**) or 10 μM TPC2-A1-P (**C**) under bi-ionic condition using a pipette (luminal) solution containing 105 mM $CaCl_2$, 5 mM HEPES and 5 mM MES (pH 4.6) and a bath (cytosolic) solution containing 160 mM NaCl and 5 mM HEPES (pH 7.2). Exemplar recordings are shown. Pooled data (mean ± s.e.m.) quantifying the fold-increase in the inward Ca^{2+} currents at −100 mV or the outward Na^+ currents at +100 mV in cells expressing the mutant channel versus the wild-type in response to TPC2-A1-N (**B**) or TPC2-A1-P (**D**). Data are from three independent experiments where each point represents the response of an individual patch. (**E–G**) Comparison of the effects of TPC2 activators on currents from HEK cells expressing wild-type or the Q314A mutant at the plasma membrane. Exemplar recordings for TPC2-A1-N and TPC2-A1-P overlaid in e are the same as those shown in (**A**, **C**), respectively. Pooled data (mean ± s.e.m.) quantifying the amplitudes of the inward Ca^{2+} currents at −100 mV and the outward Na^+ currents at +100 mV (**F**) and the reversal potentials (**G**) in cells expressing the mutant channel versus the wild-type in response to the indicated agonist. Data are from three independent experiments where each point represents the response of an individual patch. *$P = 0.02$ (unpaired *t* test).

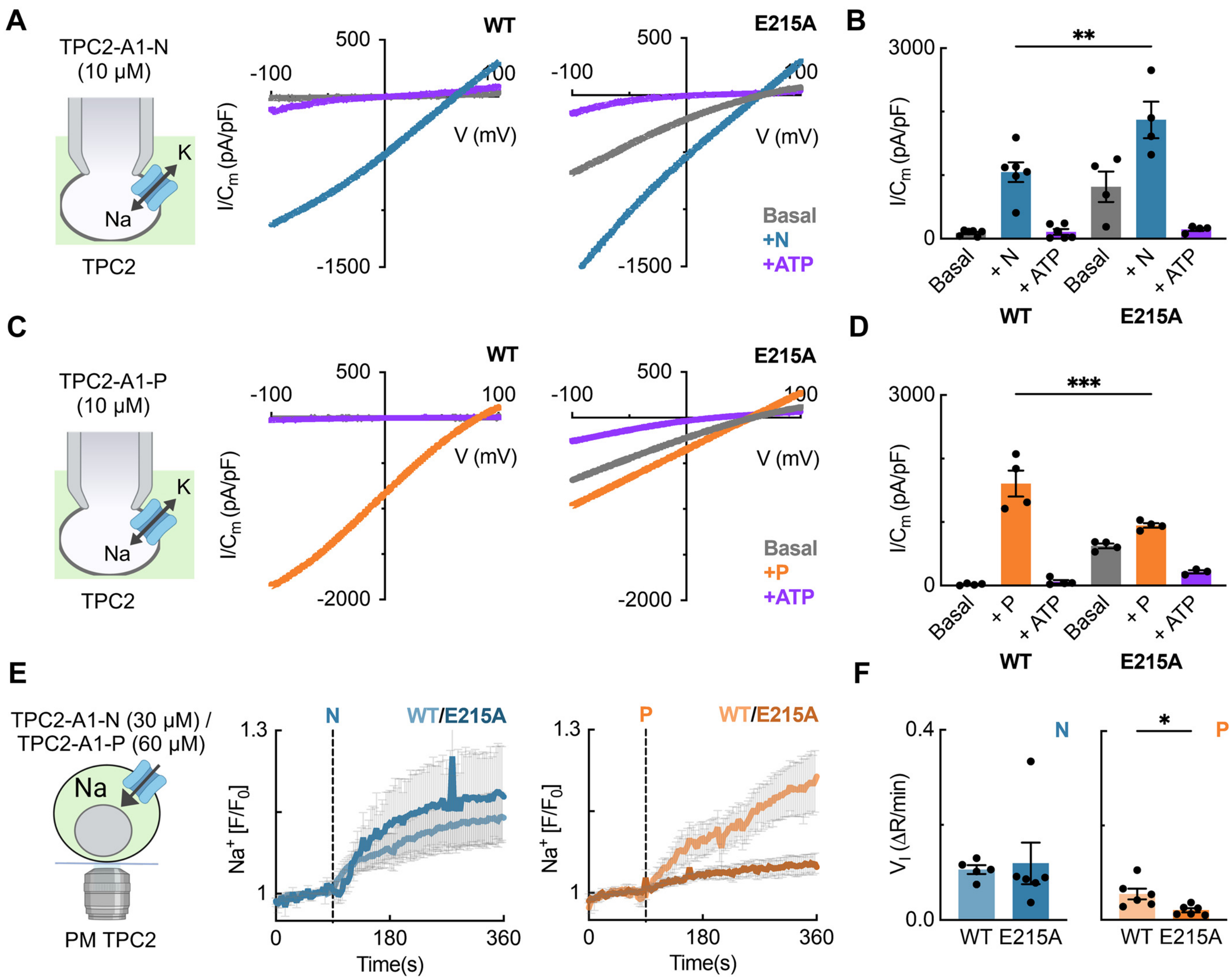

Figure EV5. Effect of the E215A TPC2 mutation on Na^+ currents and Na^+ flux.

(**A–D**) Vacuolar currents to TPC2 agonists recorded from HEK cells expressing wild-type or the E215A mutant in the lysosome using a Na^+-rich luminal solution. Enlarged vacuoles were stimulated with TPC2-A1-N (**A**) or 10 µM TPC2-A1-P (**C**) followed by ATP (1 mM) using a pipette (luminal) solution containing 140 mM Na-MSA, 5 mM K-MSA, 2 mM Ca-MSA, 1 mM $CaCl_2$, 10 mM HEPES and 10 mM MES (pH 4.6), and a bath (cytosolic) solution containing 140 mM K-MSA, 5 mM KOH, 4 NaCl mM, 0.39 mM $CaCl_2$, 1 mM EGTA and 10 mM HEPES (pH 7.2). Exemplar recordings are shown. Pooled data (mean ± s.e.m.) quantifying the inward currents at −100 mV in response to TPC2-A1-N (b) or TPC2-A1-P (**D**). Data are from four to six independent experiments where each point represents the response of an individual patch. $**P = 0.007$, $***P = 0.001$ (one-way ANOVA followed by Dunnett's post hoc test). (**E**, **F**) HeLa cells expressing the indicated TPC2 construct were loaded with the Na^+ indicator, ING-2 and stimulated with 30 µM TPC2-A1-N or 60 µM TPC2-A1-P in the presence of 2 mM extracellular Ca^{2+}. Traces in e are pooled time course data (mean ± s.e.m.) where each point combines averages from five to six independent cell populations. Pooled data (mean ± s.e.m.) quantifying the initial rate of change of the ING-2 ratio from five to six independent experiments where each point represents the mean response from an individual population is shown in (**E**). $*P = 0.02$ (unpaired t test).

 © The Author(s)
