# Supplementary material for: Allosteric switching of Ca2+ permeability in the lysosomal ion channel TPC2 underpins biased agonist activation
Source: EMBO J. 2026 Aug 7;45(18):6501–26. doi: 10.1038/s44318-026-00882-1 (PMC13578460; doi:10.1038/s44318-026-00882-1)
Supplement: Supplementary file 2 [file 44318_2026_882_MOESM1_ESM.pdf]

Appendix for Allosteric switching of Ca^{2+} permeability in the lysosomal ion channel TPC2 underpins biased agonist activation

Table of contents

Appendix Figure S1 2

Appendix Figure S2 3

Appendix Figure S3 4

Appendix Table S1 5

Appendix Table S2 6

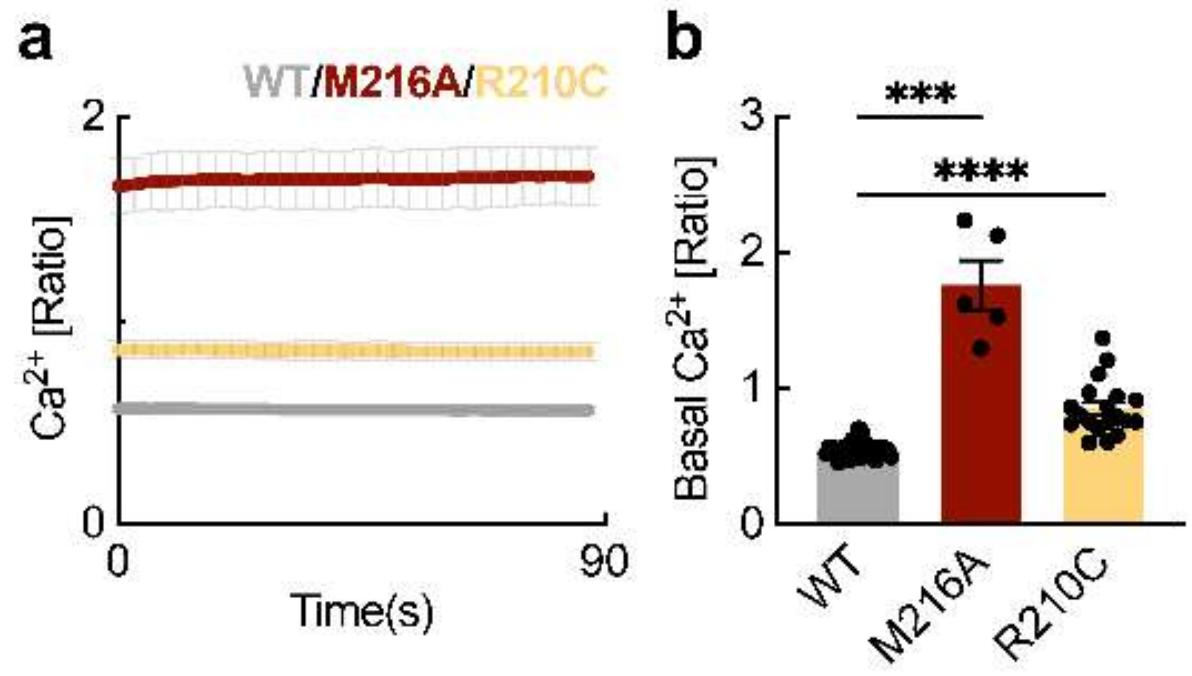

Appendix Figure S1. Effect of the M216A and R210C TPC2 mutations on basal Ca^{2+} levels. a, HeLa Cells expressing the indicated TPC2 construct were loaded with the ratiometric Ca^{2+} indicator, Fura-2 and time courses (mean ± s.e.m.) from >5 independent experiments compared in unstimulated cells where each point combines averages from multiple independent cell populations (n>5). **b**, Pooled data (mean ± s.e.m.) quantifying the basal Fura-2 ratio from >5 independent experiments where each point represents the average from an individual population expressing wild-type TPC2 or the indicated mutant. *** p = 0.0002, **** p < 0.0001 (one-way ANOVA followed by Dunnett's post hoc test).

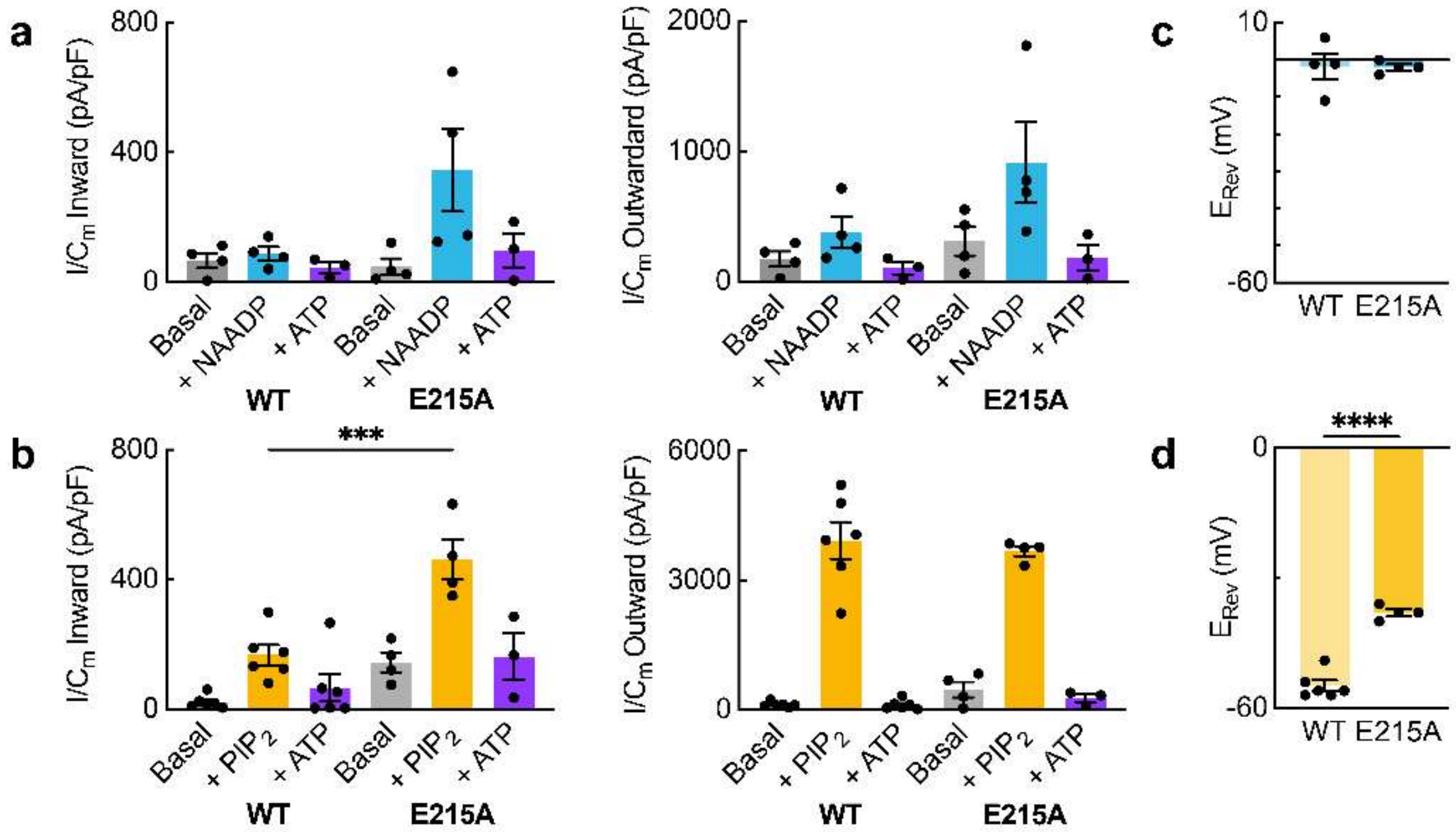

Appendix Figure S2. Effect of the E215A TPC2 mutation on Na^+ and Ca^{2+} currents. a – b, Pooled data (mean ± s.e.m.) quantifying the amplitudes of the inward Ca^{2+} currents at -60 mV and the outward Na^+ currents at +100 mV in vacuoles expressing the mutant channel versus the wild type in response to NAADP (a) and PI(3,5)P_2 (b). Data are from 4-6 independent experiments where each point represents the response of an individual patch. **** $p < 0.0001$ (One-way ANOVA followed by Dunnett's post hoc test). **c – d**, Pooled data (mean ± s.e.m.) quantifying reversal potentials in vacuoles expressing the mutant channel versus the wild type in response to NAADP (c) and PI(3,5)P_2 (d). Data are from 4-6 independent experiments where each point represents the response of an individual patch. **** $p < 0.0001$ (one-way ANOVA followed by Dunnett's post hoc test).

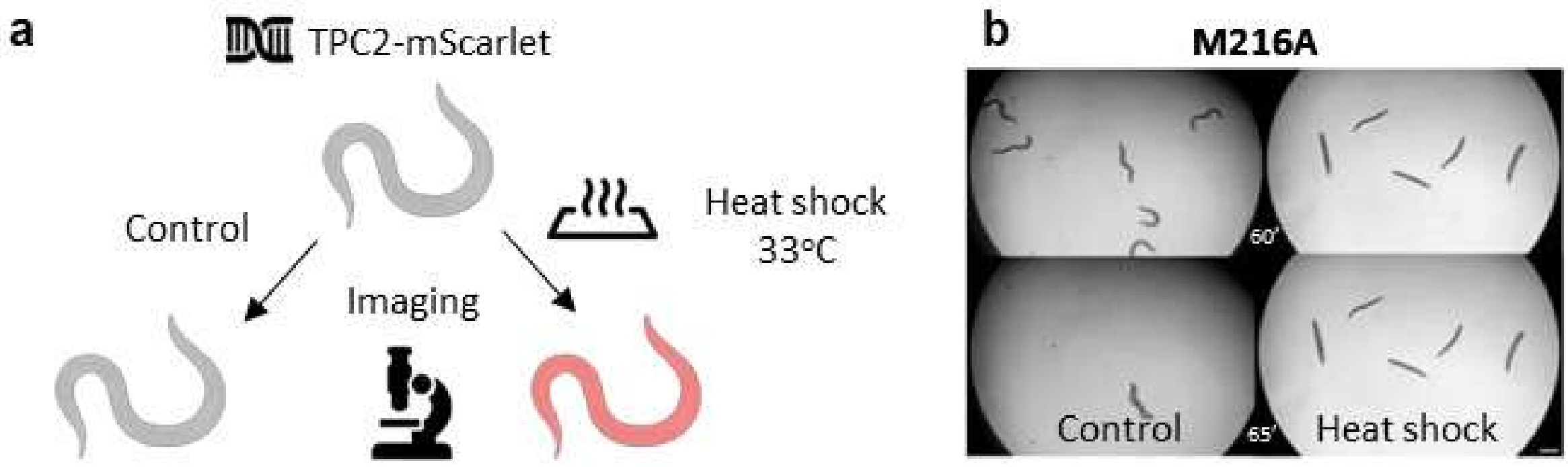

a, Schematic showing generation of transgenic *C. elegans* expressing human TPC2 under the control of a heatshock promoter and the protocol used to observe behaviour upon protein induction. **b**, Transmitted light images taken 5 min apart at the indicated times after heat shock at 33°C was terminated. Results are representiative of 3 experiments. Scale bar 500 µm.

Clone	Forward primer	Reverse primer
$TPC2^{S212A}$	CATCCGCTGGgcgCTGCCGGAAA	CATTTCAAGGTCTTCTTCATCATAGAGGAGTTCTGCAGC
$TPC2^{L213A}$	CCGCTGGTCGgcgCCGGAAATGG	ATGCATTTCAAGGTCTTCTTCATC
$TPC2^{P214A}$	CTGGTCGCTGgcgGAAATGGCCA	CGGATGCATTTCAAGGTCTTCTTCATCATAGAG
$TPC2^{E215A}$	GTCGCTGCCGgcaATGGCCAGCG	CAGCGGATGCATTTCAAGGTCTTCTTCATCATAG
$TPC2^{M216A}$	GCTGCCGGAAgcgGCCAGCGTCG	GACCAGCGGATGCATTTCAAG
$TPC2^{Q314A}$	CCGCTTCCTGgctATCATCCCCAG	AACACGATGAGCATGTTC

Appendix Table S1. Mutagenic primers used in this study.

Strain	Genotype	Origin	Description
NM5548	*jsSi1726 [loxP myo-2p::FRT::nlsCyOFP::myo-2 3' + mex-5p::FLP D5::glh-2 3' FRT3]* II	(Nonet, 2023)	rRMCE exchange site on chromosome II
ADS1002	*aeaIs10 [rgef-1p::GCaMP6s::3xNLS + lin-15(+)]*	(Susoy *et al*, 2021)	Pan-neuronal GCaMP6s
EJ1388	*jsSi1726 [loxP myo-2p::FRT::nlsCyOFP::myo-2 3' + mex-5p::FLP D5::glh-2 3' FRT3]* II; *unc-36(e251) cup-5(n3194)/qC1* III	This study	rRMCE exchange site on chromosome II with balanced *cup-5(n3194)*
EJ1427	*dxSi1 [hsp-16.2p::TPC2(+)::mScarlet::tbb-2 3']* II*; aeaIs10 [rgef-1p::GCaMP6s::3xNLS]*	This study	TPC2 and pan-neuronal GCaMP6s
EJ1428	*dxSi7 [hsp-16.2p::TPC2(E215A)::mScarlet::tbb-2 3']* II*; aeaIs10 [rgef-1p::GCaMP6s::3xNLS]*	This study	$TPC2^{E215A}$ and pan-neuronal GCaMP6s
EJ1443	*dxSi13 [hsp-16.2p::TPC2(E215A L265P)::mScarlet::tbb-2 3']* II*; aeaIs10 [rgef-1p::GCaMP6s::3xNLS]*	This study	$TPC2^{E215A/L265P}$ and pan-neuronal GCaMP6s
EJ1441	*dxSi11 [hsp-16.2p::TPC2(M216A)::mScarlet::tbb-2 3']* II*; aeaIs10 [rgef-1p::GCaMP6s::3xNLS]*	This study	$TPC2^{M216A}$ and pan-neuronal GCaMP6s
EJ1389	*dxSi1 [hsp-16.2p::TPC2(+)::mScarlet::tbb-2 3']* II*; unc-36(e251) cup-5(n3194)/qC1* III	This study	TPC2(+) with balanced *cup-5(n3194)*
EJ1425	*dxSi7 [hsp-16.2p::TPC2 E215A)::mScarlet::tbb-2 3']* II; *unc-36(e251) cup-5(n3194)/qC1* III	This study	$TPC2^{E215A}$ with balanced *cup-5(n3194)*
EJ1434	*dxSi11 [hsp-16.2p::TPC2(M216A)::mScarlet::tbb-2 3']* II*; unc-36(e251) cup-5(n3194)/qC1* III	This study	$TPC2^{M216A}$ with balanced *cup-5(n3194)*
EJ1508	*dxSi23 [hsp-16.2p::TPC2(Q314A)::mScarlet::tbb-2 3']* II*; unc-36(e251) cup-5(n3194)/qC1* III	This study	$TPC2^{Q314A}$ with balanced *cup-5(n3194)*
EJ1466	*dxSi22 [hsp-16.2p::TPC2(R210C)::mScarlet::tbb-2 3']* II;*; unc-36(e251) cup-5(n3194)/qC1* III	This study	$TPC2^{R210C}$ with balanced *cup-5(n3194)*

Appendix Table S2. *C. elegans* strains used in this study.
