# Supplementary material for: Allosteric switching of Ca2+ permeability in the lysosomal ion channel TPC2 underpins biased agonist activation
Source: EMBO J. 2026 Aug 7;45(18):6501–26. doi: 10.1038/s44318-026-00882-1 (PMC13578460; doi:10.1038/s44318-026-00882-1)
Supplement: Supplementary file 3 [file 44318_2026_882_MOESM2_ESM.pdf]

THE EMBO JOURNAL

Allosteric switching of Ca^{2+} permeability in the lysosomal ion channel TPC2 underpins biased agonist activation

Qianru Mu, Ryo Saito, Franceska Ozola, Dawid Jaslan, Pak Nin Chu, Afroditi-Maria Zaki, Marcos Rubio-Alarcon, Marco Keller, Yu Yuan, Alan Greig, Virginia Silio, Franz Bracher, Philip Biggin, Gabriela Brailoiu, Eugen Brailoiu, Jonathan Marchant, Christian Grimm, Eric Lambie, Taufiq Rahman, and Sandip Patel

Corresponding author(s): Sandip Patel (patel.s@ucl.ac.uk) , Taufiq Rahman (mtur2@cam.ac.uk)

Review Timeline:

Submission Date:	3rd Nov 25
Editorial Decision:	22nd Jan 26
Revision Received:	14th Apr 26
Editorial Decision:	8th May 26
Revision Received:	10th Jun 26
Editorial Decision:	6th Jul 26
Revision Received:	10th Jul 26
Accepted:	13th Jul 26

Editor: William Teale

Transaction Report:

(Note: With the exception of the correction of typographical or spelling errors that could be a source of ambiguity, letters and reports are not edited. Depending on transfer agreements, referee reports obtained elsewhere may or may not be included in this compilation. Referee reports are anonymous unless the Referee chooses to sign their reports.)

Dear Sandip,

Thank you again for the submission of your manuscript entitled "Remote control of ion selectivity switching in a lysosomal ion channel tempers Ca2+ permeability" (EMBOJ-2025-122930) and for your patience during the review process; please accept my apologies for the unusually long time this process has taken. We have now received reports from two referees, which I copy below.

As you can see from their comments, both thought that your manuscript could represent a useful and timely development to our understanding of selectivity switching in TPC2. That said, both of them point out a few areas in which your data will need to be strengthened, potentially requiring additional experimentation. This will require your attention before your manuscript can be published in The EMBO Journal.

Based on the overall interest expressed in the reports, however, I would like to invite you to address the comments of all referees in a revised version of the manuscript. I should add that it is The EMBO Journal policy to allow only a single major round of revision and that it is therefore important to resolve the main concerns at this stage. I believe the concerns of the referees are reasonable and addressable, but please contact me if you have any questions, need further input on the referee comments or if you anticipate any problems in addressing any of their points. Please, follow the instructions below when preparing your manuscript for resubmission.

I would also like to point out that as a matter of policy, competing manuscripts published during this period will not be taken into consideration in our assessment of the novelty presented by your study ("scooping" protection). We have extended this 'scooping protection policy' beyond the usual 3 month revision timeline to cover the period required for a full revision to address the essential experimental issues. Please contact me if you see a paper with related content published elsewhere to discuss the appropriate course of action.

When preparing your letter of response to the referees' comments, please bear in mind that this will form part of the Review Process File, and will therefore be available online to the community. For more details on our Transparent Editorial Process, please visit our website: https://link.springer.com/journal/44318/submission-guidelines#cms-Revised-submissions

Again, please contact me at any time during revision if you need any help or have further questions.

Thank you very much again for the opportunity to consider your work for publication. I look forward to your revision.

Best regards,

William

William Teale, Ph.D.
Editor
The EMBO Journal

When submitting your revised manuscript, please carefully review the instructions below and include the following items:

1) a .docx formatted version of the manuscript text (including legends for main figures, EV figures and tables). Please make sure that the changes are highlighted to be clearly visible.

2) individual production quality figure files as .eps, .tif, .jpg (one file per figure).

3) a .docx formatted letter INCLUDING the reviewers' reports and your detailed point-by-point response to their comments. As part of the EMBO Press transparent editorial process, the point-by-point response is part of the Review Process File (RPF), which will be published alongside your paper.

4) a complete author checklist, which you can download from our author guidelines (https://link.springer.com/journal/44318/submission-guidelines#cms-Revised-submissions). Please insert information in the checklist that is also reflected in the manuscript. The completed author checklist will also be part of the RPF.

5) Please note that all corresponding authors are required to supply an ORCID ID for their name upon submission of a revised manuscript.

6) We require a 'Data Availability' section after the Materials and Methods. Before submitting your revision, primary datasets

produced in this study need to be deposited in an appropriate public database, and the accession numbers and database listed under 'Data Availability'. Please remember to provide a reviewer password if the datasets are not yet public (see https://link.springer.com/partners/embo-press/editorial-policies#Data%20deposition). If no data deposition in external databases is needed for this paper, please then state in this section: This study includes no data deposited in external repositories. Note that the Data Availability Section is restricted to new primary data that are part of this study.

Note - All links should resolve to a page where the data can be accessed.

7) When assembling figures, please refer to our figure preparation guideline in order to ensure proper formatting and readability in print as well as on screen:
https://link.springer.com/journal/44318/submission-guidelines#cms-Figure-and-data-presentation

Please remember: Digital image enhancement is acceptable practice, as long as it accurately represents the original data and conforms to community standards. If a figure has been subjected to significant electronic manipulation, this must be noted in the figure legend or in the 'Materials and Methods' section. The editors reserve the right to request original versions of figures and the original images that were used to assemble the figure.

8) For data quantification: please specify the name of the statistical test used to generate error bars and P values, the number (n) of independent experiments (specify technical or biological replicates) underlying each data point and the test used to calculate p-values in each figure legend. The figure legends should contain a basic description of n, P and the test applied. Graphs must include a description of the bars and the error bars (s.d., s.e.m.).

9) We would also encourage you to include the source data for figure panels that show essential data. Numerical data can be provided as individual .xls or .csv files (including a tab describing the data). For 'blots' or microscopy, uncropped images should be submitted (using a zip archive or a single pdf per main figure if multiple images need to be supplied for one panel). Additional information on source data and instruction on how to label the files are available at
https://link.springer.com/journal/44318/submission-guidelines#cms-Source-data

10) We replaced Supplementary Information with Expanded View (EV) Figures and Tables that are collapsible/expandable online (see examples in https://www.embopress.org/doi/10.15252/embj.201695874). A maximum of 5 EV Figures can be typeset. EV Figures should be cited as 'Figure EV1, Figure EV2" etc. in the text and their respective legends should be included in the main text after the legends of regular figures.

- For the figures that you do NOT wish to display as Expanded View figures, they should be bundled together with their legends in a single PDF file called *Appendix*, which should start with a short Table of Content. Appendix figures should be referred to in the main text as: "Appendix Figure S1, Appendix Figure S2" etc. See detailed instructions regarding expanded view here: < https://link.springer.com/journal/44318/submission-guidelines#cms-Expanded-View-data >.

- Additional Tables/Datasets should be labelled and referred to as Table EV1, Dataset EV1, etc. Legends have to be provided in a separate tab in case of .xls files. Alternatively, the legend can be supplied as a separate text file (README) and zipped together with the Table/Dataset file.

11) At EMBO Press we ask authors to provide source data for the main manuscript figures. Our source data coordinator will contact you to discuss which figure panels we would need source data for and will also provide you with helpful tips on how to upload and organize the files.

12) Our journal encourages inclusion of *data citations in the reference list* to directly cite datasets that were re-used and obtained from public databases. Data citations in the article text are distinct from normal bibliographical citations and should directly link to the database records from which the data can be accessed. In the main text, data citations are formatted as follows: "Data ref: Smith et al, 2001" or "Data ref: NCBI Sequence Read Archive PRJNA342805, 2017". In the Reference list, data citations must be labeled with "[DATASET]". A data reference must provide the database name, accession number/identifiers and a resolvable link to the landing page from which the data can be accessed at the end of the reference. Further instructions are available at < https://link.springer.com/journal/44318/submission-guidelines#cms-Reference-guidelines >.

13) In order to increase the reproducibility and reach of your work, The EMBO Journal includes a table of reagents that were used in the study. Please provide this along with your revisions.

Further instructions for preparing your revised manuscript:

Read our guidance for manuscript revisions and related editorial policies: https://link.springer.com/journal/44318/submission-guidelines#cms-Revised-submissions

Please make sure you upload a letter of response to the referees' comments together with the revised manuscript.

Please also check that the title and abstract of the manuscript are brief, yet explicit, even to non-specialists.

When assembling figures, please refer to our figure preparation guideline in order to ensure proper formatting and readability in print as well as on screen:
https://media.springernature.com/original/springer-cms/rest/v1/content/27825798/data/v1

At EMBO Press we ask authors to provide source data for the main manuscript figures. You will receive a separate email with instructions for providing source data with your revised manuscript, including how to upload and organize the files.

IMPORTANT: When you send the revision we will require
- a point-by-point response to the referees' comments, with a detailed description of the changes made (as a word file).
- a word file of the manuscript text.
- individual production quality figure files (one file per figure)
- a complete author checklist
- Expanded View files (replacing Supplementary Information)
- a Reagents and Tools Table as part of the Methods section

Please remember: Digital image enhancement is acceptable practice, as long as it accurately represents the original data and conforms to community standards. If a figure has been subjected to significant electronic manipulation, this must be noted in the figure legend or in the 'Materials and Methods' section. The editors reserve the right to request original versions of figures and the original images that were used to assemble the figure.

We realize that it is difficult to revise to a specific deadline. In the interest of protecting the conceptual advance provided by the work, we recommend a revision within 3 months (22nd Apr 2026). Please discuss the revision progress ahead of this time with the editor if you require more time to complete the revisions. Use the link below to submit your revision:

https://emboj.msubmit.net/cgi-bin/main.plex

Referee #1:

TPC2 is an unusual channel in which the physiological agonists NAADP and PI(3,5)P2 dictate the channel selectivity. In previous studies, the authors reported that NAADP set the channel pore permeable to Na+ and Ca2+ while PI(3,5)P2 set the pore as Na+ permeable channel. They also showed that the agonists act synergistically to specifically increase Ca2+ permeability. The mechanism and channel domains controlling the activator-specific pore properties and their combination are not known. In the present interesting studies, the authors identified an S4-S5 linker and key residues within the domain that appears to determine the selectivity filter ionic selectivity and in particular Ca2+ flux. The authors then show that when the channel is mutated to increase Ca2+ selectivity it becomes toxic in vivo in the model animals C. Elegans.

The findings are very informative and important because ion selectivity switching to this extent is unprecedented. I have several comments that the authors might consider to improve their study.

1. The authors rely on measurand of synergy between N+P in increasing cytoplasmic Ca2+ as a reporter of change in reversal potential and thus pore selectivity. It is not clear that loss of synergism is equivalent to changes in pore selectivity. The evidence that the S4-S5 linker indeed controls pore selectivity and the central role of E215 can be strengthened by measuring reversal potential by mutating residues that appears to contact E215 such as Q314A or other residues reported in this study in the linker or that contact E215.

2. The authors rely on total expression and confocal images in concluding similar surface expression of the wild-type and mutants TPC2. Because of the large and variable confocal images, to better quantify effect of surface expression on extent of current, the authors could use either biotinylation or TIRF to do so.

3. Do N+P treatment of C. Elegans expressing wild-type TPC2 affect motility similar to E215A? This should be independent verification that change in channel selectivity and not just the extent of lysosomal Ca2+ release causes toxicity and death.

Minor

1. Page 4, correct TPC2213A to TPCL2213A

2. Page 6, change Fig. 3f to 3e and with no panel h change 3g-h to 3g.
3. The synergy in panel f is lacking while in panel h it is substantial. What is the reason for the difference between the two sets of data and why not combine them?
4. Fig 6d: The double mutant E215A_L265P is not mentioned or discussed in the text.

Referee #2:

Review report of manuscript entitled "Remote control of ion selectivity switching in a lysosomal ion channel tempers Ca2+ permeability"

- general summary and opinion about the principle significance of the study, its questions and findings

Selectivity filters of ion channels are considered to be hardwired, however, two pore channels (TPCs) are thought to be able to perform agonist-specific switching of ion selectivity. Such a concept has not been reported for other known ion channels. TPC2 is permeable to Ca2+, can be activated via NAADP and seems to be selective to Na+ when activated with the lipid PI(3,5)P2, while co-stimulation increases Ca2+ but not Na+ permeability. Here, authors describe a mutation in the distal cytosolic linker connecting the first voltage-sensing-like domain and the pore region which results in a TPC2 channel only permeable to Ca2+, independent of the agonist. Functionally this results in exaggerated lysosomal Ca2+ flux and disrupts locomotion and viability of C. elegans in an in vivo model. A disease-liked proximal mutation in TPC2 results in similar behaviour.

Authors used site-directed mutagenesis, Ca2+ imaging, patch-clamp, molecular dynamics simulations, and transgenic C. elegans experiments to reveal key residues close to the selectivity filter to control ionic selectivity and flux in TPC2. Thereby, they claim to convert TPC2 into a "regular" ion channel rendering it unable to distinguish agonists and with properties resembling the co-activated state. Further authors conclude that agonist-dependent ion selectivity switching in TPC2 is an essential organellar safeguard mechanism.

This manuscript covers a timely topic, utilizing state of the art methodologies, and addresses a relevant research question in the field. I only have one main concern and a few minor comments stated below.

- specific concerns essential to be addressed to support the conclusions

The study is highly interesting, well written and results are clearly depicted. However, to me it appears the claim made regarding ion channel selectivity "switching" cannot directly be supported by the experiments presented.

What was the justification for the solutions chosen in bi-ionic conditions? Particularly, it remains unclear, why the electrophysiologic patch-clamp experiments to study selectivity of TPC2 were performed in bi-ionic conditions only testing one condition (luminal Ca2+, cytosolic Na+) - no selectivity sequence was established. Regarding permeability, it might be a valid approach, but this point needs to be clarified throughout the manuscript.

Additionally, I would suggest, testing the main claims using patch solutions as close to the physiologic conditions as possible, also including potassium (e.g. K-MSA) on the cytosolic side and NaCl or Na-MSA in the luminal solution. With the experimental conditions used, one cannot conclude about Na+ flux into the cytosol. Using 150 mM NaCl in the cytosolic solution does not reflect the concentration of Na+ in the cytosol which usually is around 12 mM, while on the luminal side of the lysosome it is much higher around 100 mM.

Authors may also address effects of the agonists on Na+ fluxes by measuring cytosolic Na+ concentrations, using e.g. the ratiometric Na+ indicator SBFI in intact cells.

- minor concerns that should be addressed

Please indicate the ionic conditions used for the experiments more clearly either in the figures or legends. For instance, it is not stated in Fig. 1 or Fig. 5 that 2 mM Ca2+ were present in the bath solution during whole-cell calcium imaging experiments.

Fig. 2: As mentioned above authors may specify, why the specific conditions were analyzed.

In Fig. 3 c and d, authors utilized Fura-FF, which is a low affinity Ca2+ indicator, used to record high Ca2+ concentrations. Such high concentrations would also be detectable in intact lysosomes or other organelles e.g. the ER. How did the authors exclude a contribution from these organelles in intact cells? This is not clearly described in the methods or results sections.

Fig. 5: Authors performed the analysis of all mutations in the background of L11A and L12A to re-direct the channel to the plasma membrane. However, the lipid composition of the PM is markedly distinct from the lysosomal membrane and does not contain PI(3,5)P2. Thus, the lipid environment is evidently different from the natural environment of TPC2 and the stimulation

with PI(3,5)P2 or synthetic mimicries may be affected. Therefore, authors patched TPC2 in enlarged lysosomes (vacuoles) for the E215A mutant. What were the experimental conditions - which solutions were applied? How did the authors ensure the presence of NAADP accessory proteins (LMS12 or JPT2) in excised plasma membrane patches when applying NAADP? Did authors consider using this method or excised patches from enlarged lysosomes to confirm their obtained results in a more physiologic solution?

Why were outward current amplitudes extracted at different voltages (+80 mV versus +85 mV) in vacuole patching in Fig. 5 and Fig. S4?

Response to Referees

Referee #1:

TPC2 is an unusual channel in which the physiological agonists NAADP and PI(3,5)P2 dictate the channel selectivity. In previous studies, the authors reported that NAADP set the channel pore permeable to Na+ and Ca2+ while PI(3,5)P2 set the pore as Na+ permeable channel. They also showed that the agonists act synergistically to specifically increase Ca2+ permeability. The mechanism and channel domains controlling the activator-specific pore properties and their combination are not known. In the present interesting studies, the authors identified an S4-S5 linker and key residues within the domain that appears to determine the selectivity filter ionic selectivity and in particular Ca2+ flux. The authors then show that when the channel is mutated to increase Ca2+ selectivity it becomes toxic in vivo in the model animals C. Elegans.

The findings are very informative and important because ion selectivity switching to this extent is unprecedented. I have several comments that the authors might consider to improve their study.

RESPONSE: We thank the Reviewer for their positive comments.

1. The authors rely on measurand of synergy between N+P in increasing cytoplasmic Ca2+ as a reporter of change in reversal potential and thus pore selectivity. It is not clear that loss of synergism is equivalent to changes in pore selectivity. The evidence that the S4-S5 linker indeed controls pore selectivity and the central role of E215 can be strengthened by measuring reversal potential by mutating residues that appears to contact E215 such as Q314A or other residues reported in this study in the linker or that contact E215.

RESPONSE: We have performed electrophysiological analysis of the Q314A mutant. These data are shown in **Figure EV4**. Similar to the E215A mutant, we find an increase in the Na^+ current for TPC2-A1-N but not TPC2-A1-P, an increase in Ca^{2+} current for both and a positive shift in the reversal potential for TPC2-A1-P. The only slight difference between the mutants is that the increase in Na^+ current for TPC2-A1-N is more modest for the Q314A than E215A.

This data is described on **page 7** as follows:

Electrophysiological analysis of PM $TPC2^{Q314A}$ in macropatches excised from the plasma membrane is shown in Fig. EV4. As with the E215A mutation, Q314A supported larger Ca^{2+} currents to both TPC1-A1 N (Fig. EV4a-b) and TPC2-A1-P (Fig. EV4c-d) compared to the wild-type channel. TPC2-A1-P-evoked Na^+ currents were unaffected by the mutation, thereby again rendering the channel less able to discriminate its agonists (Fig. EV4e-f). And the reversal potential for TPC2-A1-P currents was similarly positively shifted upon mutation (Fig. EV4g). Thus, PM $TPC2^{Q314A}$ and PM $TPC2^{E215A}$ behaved similarly at the channel level.

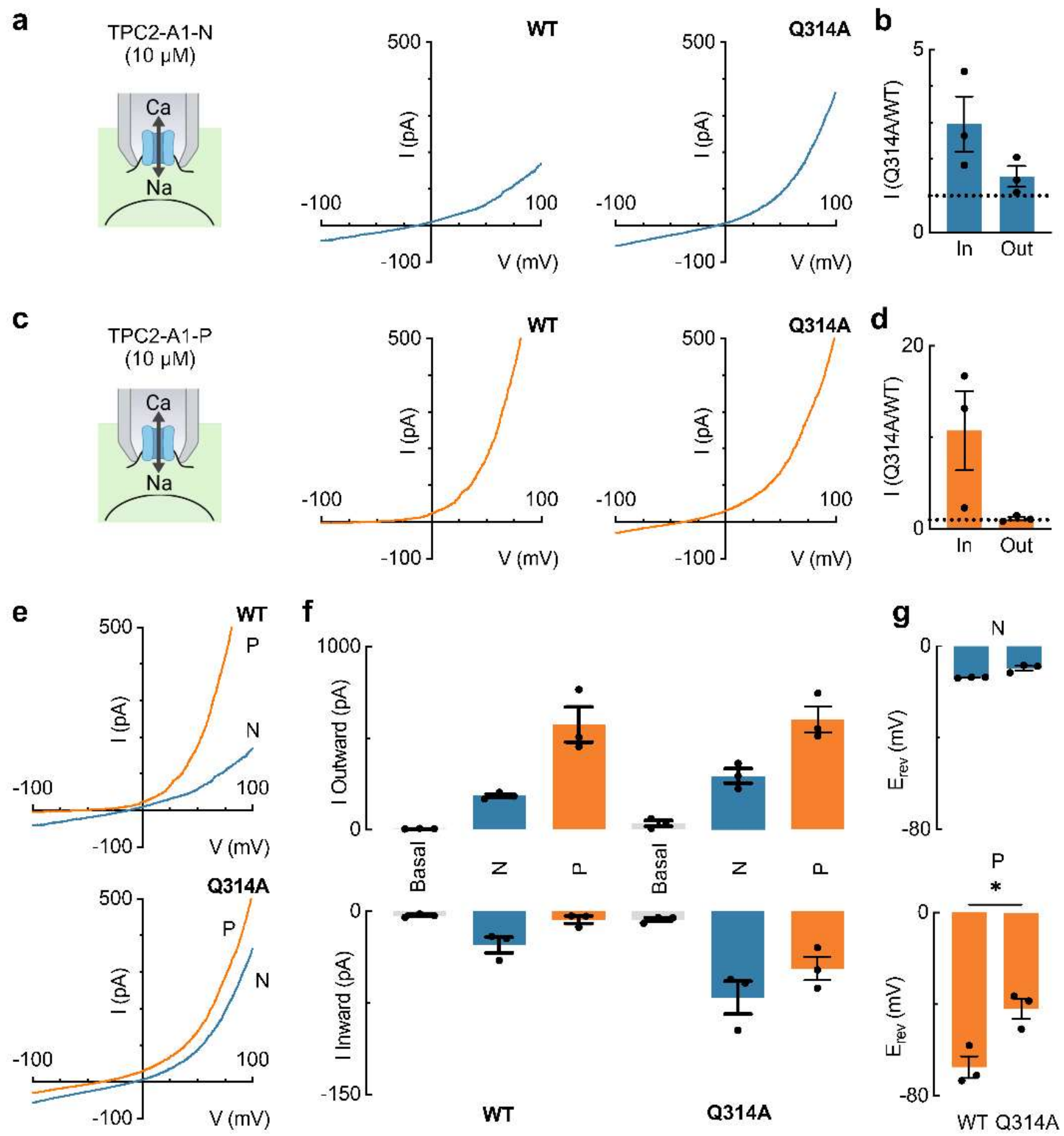

Figure EV4. Effect of the Q314A TPC2 mutation on Na^+ and Ca^{2+} currents.

a - d, Macropatches from HEK cells expressing the indicated TPC2 construct were stimulated with 10 µM TPC2-A1-N (a) or 10 µM TPC2-A1-P (c) under bi-ionic conditions using a pipette (luminal) solution containing 105 mM $CaCl_2$, (pH 4.6) and a bath (cytosolic) solution containing 160 mM NaCl (pH 7.2). Exemplar recordings are shown**.** Pooled data (mean ± s.e.m.) quantifying the fold-increase in the inward Ca^{2+} currents at -100 mV or the outward Na^+ currents at +100 mV in cells expressing the mutant channel versus the wild type in response to TPC2-A1-N (b) or TPC2-A1-P (d). Data are from 3 independent experiments where each point represents the response of an individual patch.

e – g, Comparison of the effects of TPC2 activators on currents from HEK cells expressing wild type or the Q314A mutant at the plasma membrane. Exemplar recordings for TPC2-A1-N and TPC2-A1-P overlaid in e are the same as those shown in panel a and c, respectively. Pooled data (mean ± s.e.m.) quantifying the amplitudes of the inward Ca^{2+} currents at -100 mV and the outward Na^+ currents at +100 mV (f) and the reversal potentials (g) in cells expressing the mutant channel versus the wild type in response to the indicated agonist. Data are from 3 independent experiments where each point represents the response of an individual patch. * $p < 0.05$ (Unpaired t-test).

2. The authors rely on total expression and confocal images in concluding similar surface expression of the wild-type and mutants TPC2. Because of the large and variable confocal images, to better quantify effect of surface expression on extent of current, the authors could use either biotinylation or TIRF to do so.

RESPONSE: We have performed TIRF microscopy in cells expressing the wild-type channel and the most important gain-of-function mutants (E215A, Q314A and R210C). These data are shown in

Figure EV2. We do not find any quantitative difference in expression. If anything, there is a tendency for expression to be reduced which would underestimate the reported potentiating effect of the mutation. These data are described on **page 5** as follows:

No significant differences were found in expression of PM TPC2 and PM TPC2^{E215A} using TIRF microscopy to assess expression at the cell surface (Fig. EV2).

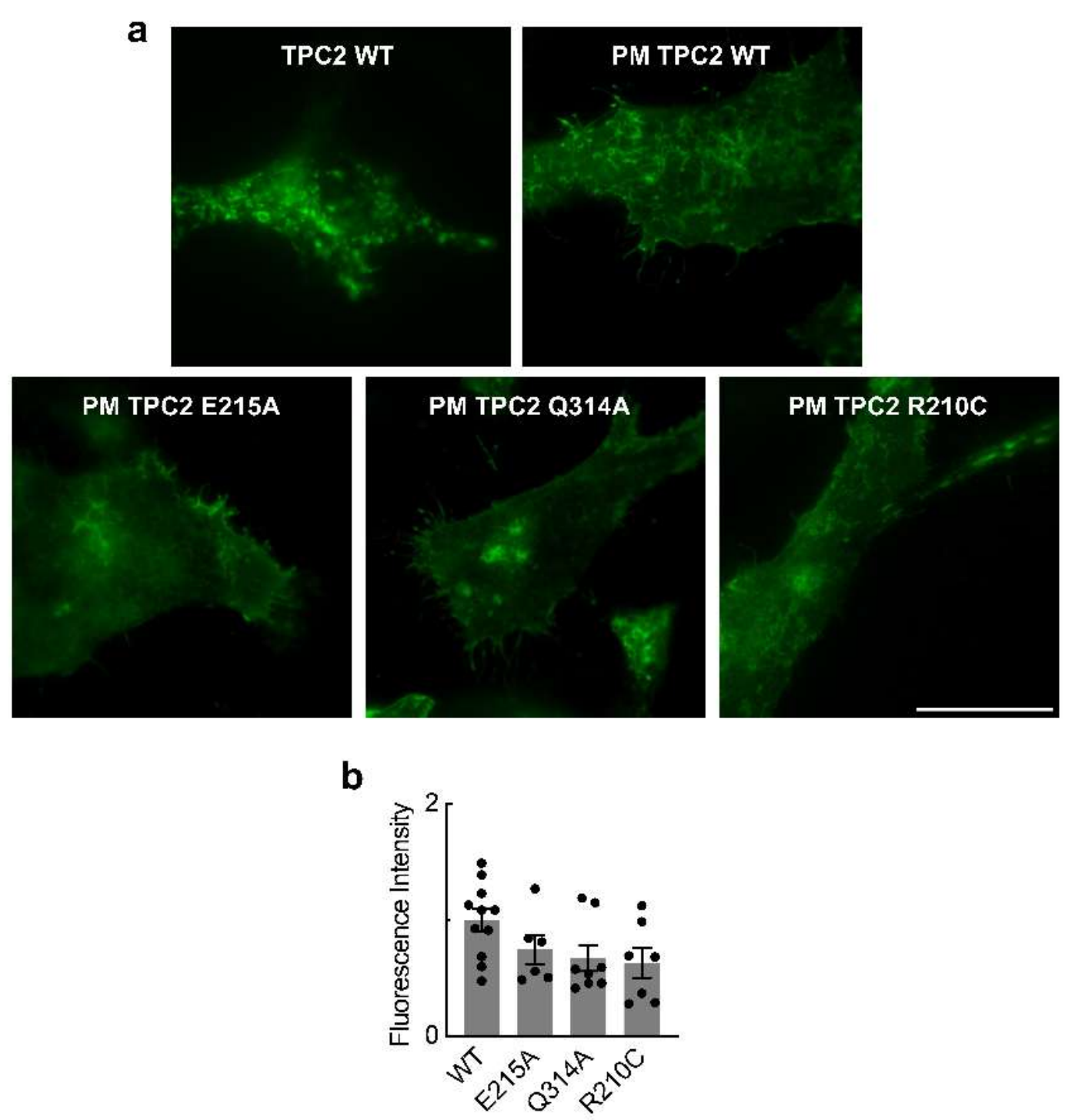

Figure EV2. Expression analysis of plasma membrane targeted TPC2.

a, Exemplar TIRF images of the indicated GFP-tagged TPC2 constructs expressed in HeLa cells. Scale bar 20 µm.

b, Pooled data (mean ± s.e.m.) quantifying fluorescence from 2 independent transfections where each point represents the intensity from an individual cell expressing the indicated plasma membrane-targted mutant. Data are normalised to wild type PM TPC2.

Methods have been updated accordingly.

3. Do N+P treatment of C. Elegans expressing wild-type TPC2 affect motility similar to E215A? This should be independent verification that change in channel selectivity and not just the extent of lysosomal Ca2+ release causes toxicity and death.

RESPONSE: This is a great suggestion. But compound penetration is an issue in these *in vivo* experiments. We attempted microinjecting the agonists into the intestine but ran into problems with precipitation at the high concentrations required to ensure even distribution throughout the animal.

Minor

1. Page 4, correct TPC2213A to TPCL2213A

RESPONSE: Corrected.

2. Page 6, change Fig. 3f to 3e and with no panel h change 3g-h to 3g.

RESPONSE: Corrected.

3. The synergy in panel f is lacking while in panel h it is substantial. What is the reason for the difference between the two sets of data and why not combine them?

RESPONSE: We presume the reviewer is referring to Figure 4. Please note that panel f shows a representative experiment whereas panel h is the pooled data. We do not think there is evidence for synergy in panel h as the Q314A mutation substantially increases the response to TPC2-A1-N (left panel) and TPC2-A1-P (middle panel) but only modestly increases the response to the combination (right panel) relative to the wild-type channel. The increase in the response to the combination upon mutation can be accounted for by the effect of the mutation on the agonists alone i.e. an additive effect. This is quantified in panel j.

4. Fig 6d: The double mutant E215A_L265P is not mentioned or discussed in the text.

RESPONSE: This was mentioned on page 9 as follows:

To determine whether paralysis was due to channel activity, we generated ’pore-dead’ animals by mutating L265 in the first pore-helix of TPC2. The resulting double transgenics ($TPC2^{E215A/L265P}$) behaved similarly to the strain expressing wild-type TPC2 whereby heat shock had little effect on motility (Fig. 6d).

Referee #2:

Review report of manuscript entitled "Remote control of ion selectivity switching in a lysosomal ion channel tempers Ca2+ permeability"

- general summary and opinion about the principle significance of the study, its questions and findings

Selectivity filters of ion channels are considered to be hardwired, however, two pore channels (TPCs) are thought to be able to perform agonist-specific switching of ion selectivity. Such a concept has not been reported for other known ion channels. TPC2 is permeable to Ca2+, can be activated via NAADP and seems to be selective to Na+ when activated with the lipid PI(3,5)P2, while co-stimulation increases Ca2+ but not Na+ permeability. Here, authors describe a mutation in the distal cytosolic linker connecting the first voltage-sensing-like domain and the pore region which results in a TPC2 channel only permeable to Ca2+, independent of the agonist. Functionally this results in exaggerated lysosomal Ca2+ flux and disrupts locomotion and viability of C. elegans in an in vivo model. A disease-liked proximal mutation in TPC2 results in similar behaviour.

Authors used site-directed mutagenesis, Ca2+ imaging, patch-clamp, molecular dynamics simulations, and transgenic C. elegans experiments to reveal key residues close to the selectivity filter to control ionic selectivity and flux in TPC2. Thereby, they claim to convert TPC2 into a "regular" ion channel rendering it unable to distinguish agonists and with properties resembling the co-activated state. Further authors conclude that agonist-dependent ion selectivity switching in TPC2 is an essential organellar safeguard mechanism.

This manuscript covers a timely topic, utilizing state of the art methodologies, and addresses a relevant research question in the field. I only have one main concern and a few minor comments stated below.

- specific concerns essential to be addressed to support the conclusions

The study is highly interesting, well written and results are clearly depicted. However, to me it appears the claim made regarding ion channel selectivity "switching" cannot directly be supported by the experiments presented.

RESPONSE: We thank the Reviewer for their positive comments

What was the justification for the solutions chosen in bi-ionic conditions? Particularly, it remains unclear, why the electrophysiologic patch-clamp experiments to study selectivity of TPC2 were performed in bi-ionic conditions only testing one condition (luminal Ca2+, cytosolic Na+) - no selectivity sequence was established. Regarding permeability, it might be a valid approach, but this point needs to be clarified throughout the manuscript.

RESPONSE: We chose Ca^{2+} and Na^{+} here and in our previous analyses (Gerndt *et al*, 2020; Saito *et al*, 2023; Yuan *et al*, 2022) because they are considered most physiologically relevant. These ions represent an initial focus of the TPC field on the actions of the Ca^{2+} mobilizing messenger NAADP moving to a more Na^{+}-centric view upon discovery of channel activation by $PI(3,5)P_2$. Bi-ionic tests with luminal Ca^{2+} also allowed us to accurately measure Ca^{2+} currents. These currents are small and thus challenging to characterize.

Please note that we have previously distinguished the TPC2 agonists using a luminal solution containing both Ca^{2+} and Na^{+} (Gerndt *et al.*, 2020)(reproduced below). This highlights the challenges of measuring Ca^{2+} currents reflected by the modest changes in reversal potentials. Never-the-less, this analysis confirmed the very different actions of TPC agonists with the bionic solutions containing only Ca^{2+} or Na^{+} used here.

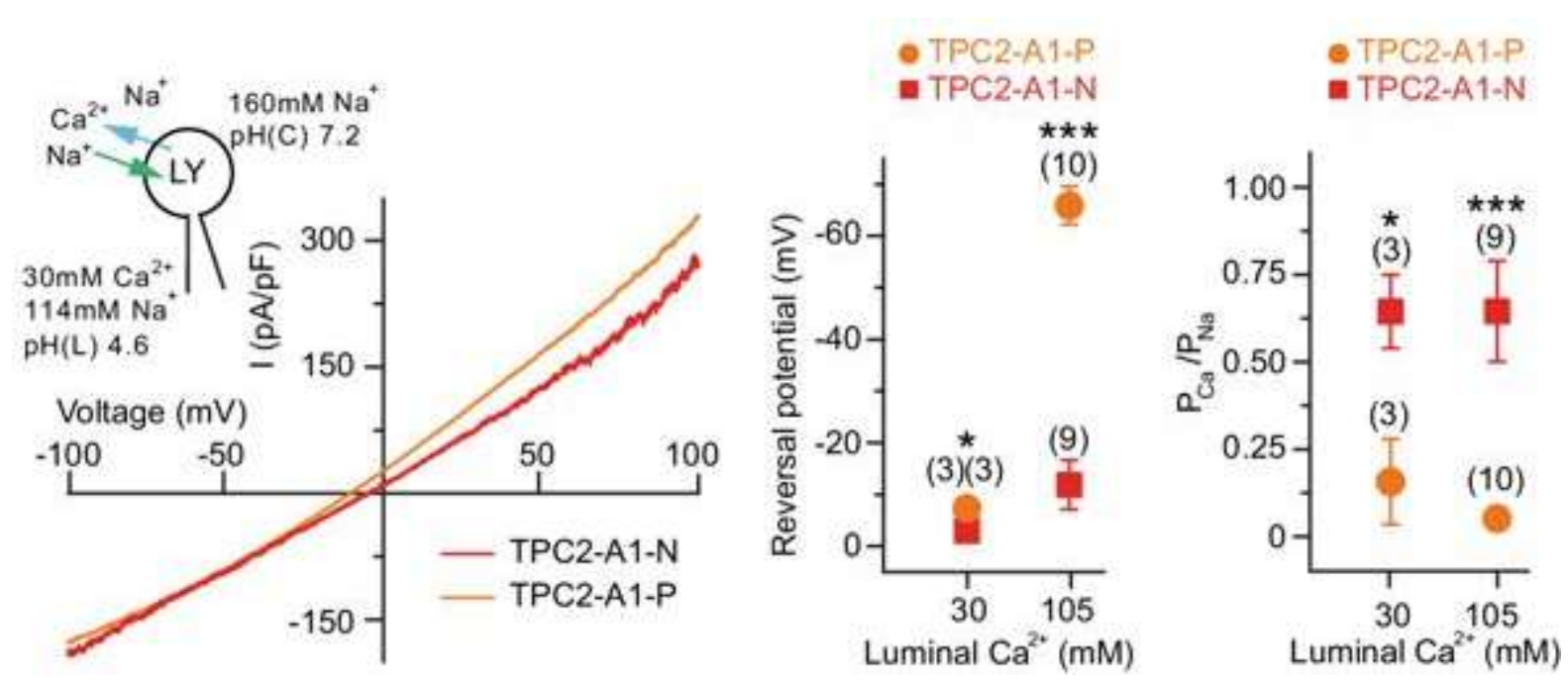

Endo-lysosomal patch-clamp analysis of compound effects under different bi-ionic conditions. Left, Agonist-evoked cation currents from enlarged endo-lysosomes isolated from HEK293 cells stably expressing human TPC2 using a luminal solution containing 114 mM Na^{+} and 30 mM Ca^{2+}, pH 4.6; and a bath solution containing 160 mM Na^{+}, pH 7.2 (n = 3, each). Statistical analyses of E_{rev} (**middle**) and permeability ratio (P_{Ca}/P_{Na}) (**right**) compared to those derived under bi-ionic conditions used in the present study (105 mM $CaCl_2$). *p<0.05 and ***p<0.001, unpaired t-test. Data are from Figure 2—figure supplement 1 in (Gerndt *et al.*, 2020).

This justification is now included on page 5 as follows:

These experiments were performed under bi-ionic conditions (luminal Ca^{2+}, cytosolic Na^{+}) as reported previously (Gerndt *et al.*, 2020; Saito *et al.*, 2023; Yuan *et al.*, 2022) to allow measurement of the small Ca^{2+} currents relative to the larger Na^{+} currents.

Relative permeability ratios measured using bi-ionic conditions are often equated with ion selectivity (for example see: (Guo *et al*, 2017)). Never-the-less, we have refrained as best we can in using the term ion selectivity in the context of distinct agonist actions. This includes a revised title:

Remote control of Ca^{2+} permeability in a lysosomal ion channel underpins biased agonist activation

And a number of other (tracked) changes throughout.

Additionally, I would suggest, testing the main claims using patch solutions as close to the physiologic conditions as possible, also including potassium (e.g. K-MSA) on the cytosolic side and NaCl or Na-MSA in the luminal solution. With the experimental conditions used, one cannot conclude about Na+ flux into the cytosol. Using 150 mM NaCl in the cytosolic solution does not reflect the concentration of Na+ in the cytosol which usually is around 12 mM, while on the luminal side of the lysosome it is much higher around 100 mM.

RESPONSE: Thank you for this suggestion. We have performed additional vacuolar patch clamp analyses for the wild type and the E215A mutant using a luminal solution containing high Na^{+} (in mM: 140 Na-MSA, 5 K-MSA, 2 Ca-MSA, 1 $CaCl_2$, 10 HEPES and 10 MES, pH 4.6) and a cytosolic solution containing hight K^{+} (in mM: 140 K-MSA, 5 KOH, 4 NaCl, 0.39 $CaCl_2$, 1 EGTA and 10 HEPES, pH 7.2). These data are shown in **Figure EV5a-d**. Interestingly, we noted a substantial increase in the basal inward current upon mutation (Fig. EV5a-b). Nevertheless, similar to the bi-ionic conditions, the E215A mutation increased currents to TPC2-A1-N but not to TPC2-A1P (Fig. EV5a-d). Rather the latter appeared suppressed.

Authors may also address effects of the agonists on Na+ fluxes by measuring cytosolic Na+ concentrations, using e.g. the ratiometric Na+ indicator SBFI in intact cells.

RESPONSE: We have performed Na^{+} imaging experiments using ING-2 for the wild type and the E215A mutant. Experiments were performed using plasma membrane-targeted TPC2 and our standing HEPES-buffered saline solution containing 140 mM NaCl. These data are shown in **Figure EV5e-f**. Again, we find differential effects of the mutation on TPC2-A1-N and TPC2-A1P (Fig. EV5e-f) similar to the vacuolar patch-clamp experiments using more physiological solutions (Fig, EV5a-d).

Both the new electrophysiological data and Na^{+} imaging are described on **page 8** as follows:

We performed an additional set of vacuolar patch-clamp experiments using more physiological solutions (high luminal Na^{+}, high cytosolic K^{+}). Interestingly, we noted a substantial increase in the basal inward (predominantly Na^{+}) currents under these conditions (Fig. EV5a-b). Nevertheless, similar to the bi-ionic conditions, the E215A mutation increased currents to TPC2-A1-N but not to TPC2-A1P (Fig. EV5a-d). Rather the latter appeared suppressed. Similar apparent reduction of the

effects of TPC2-A1-P were obtained in Na^+ imaging experiments using PM TPC2 (Fig. EV5e-f).

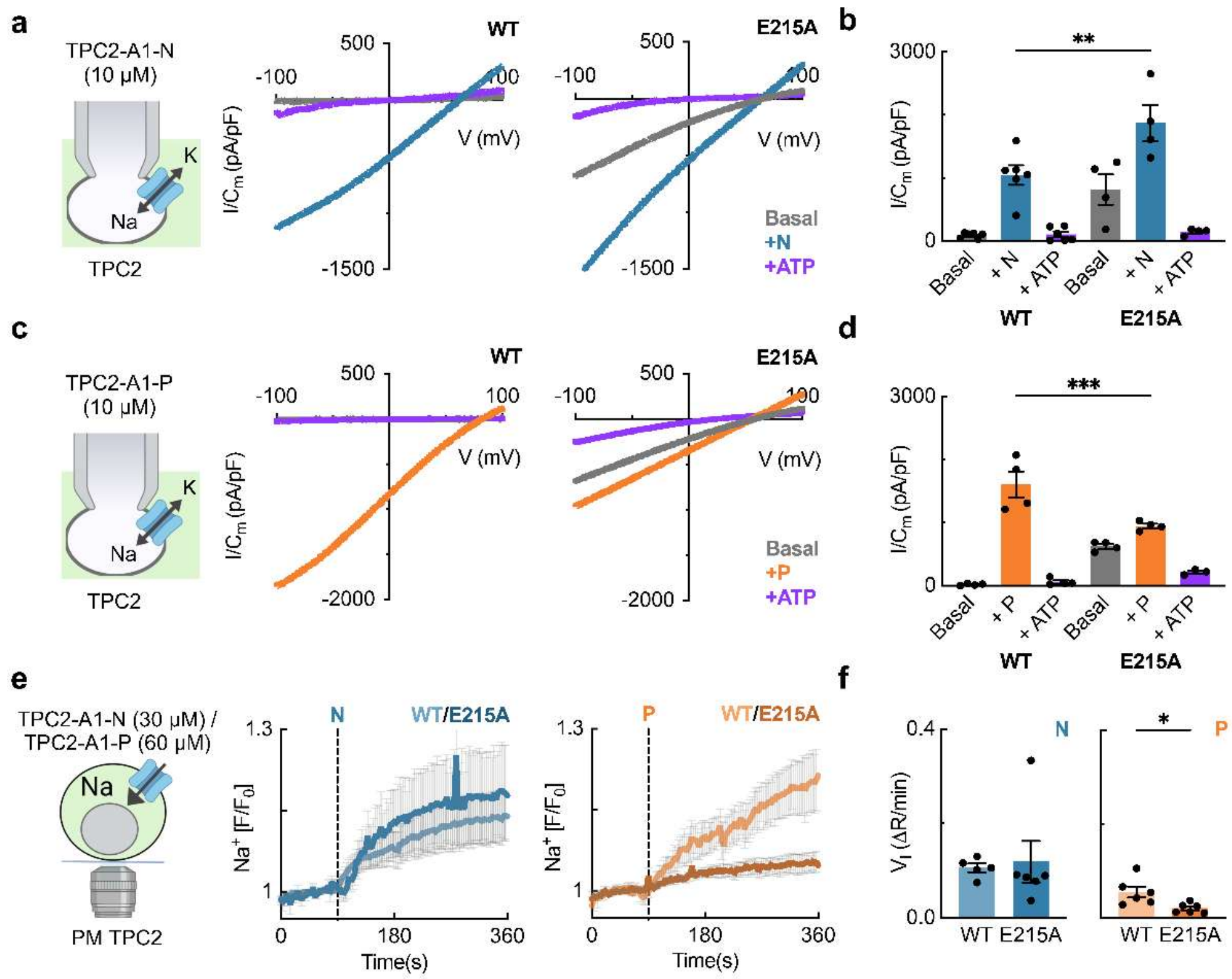

Figure EV5. Effect of the E215A TPC2 mutation on Na^+ currents and Na^+ flux.

a - d, Vacuolar currents to TPC2 agonists recorded from HEK cells expressing wild type or the E215A mutant in the lysosome using a Na^+-rich luminal solution. Enlarged vacuoles were stimulated with TPC2-A1-N (a) or 10 µM TPC2-A1-P (c) followed by ATP (1 mM) using a pipette (luminal) solution containing 140 mM Na-MSA, 5 mM K-MSA, 2 mM Ca-MSA, 1 mM $CaCl_2$, 10 mM HEPES and 10 mM MES (pH 4.6), and a bath (cytosolic) solution containing 140 mM K-MSA, 5 mM KOH, 4 NaCl mM, 0.39 mM $CaCl_2$, 1 mM EGTA and 10 mM HEPES (pH 7.2). Exemplar recordings are shown. Pooled data (mean ± s.e.m.) quantifying the inward currents at -100 mV in response to TPC2-A1-N (b) or TPC2-A1-P (d). Data are from 4 -6 independent experiments where each point represents the response of an individual patch. ** $p < 0.01$ (One-way ANOVA).

e – f, HeLa cells expressing the indicated TPC2 construct were loaded with the Na^+ indicator, ING-2 and stimulated with 30 µM TPC2-A1-N or 60 µM TPC2-A1-P in the presence of 2 mM extracellular Ca^{2+}. Traces in e are pooled time course data (mean ± s.e.m.) where each point combines averages from 5-6 independent cell populations. Pooled data (mean ± s.e.m.) quantifying the initial rate of change of the ING-2 ratio from 5-6 independent experiments where each point represents the mean response from an individual population is shown in e. *$p < 0.05$ (Unpaired t-test).

Methods have been updated accordingly.

- minor concerns that should be addressed

Please indicate the ionic conditions used for the experiments more clearly either in the figures or legends. For instance, it is not stated in Fig. 1 or Fig. 5 that 2 mM Ca2+ were present in the bath solution during whole-cell calcium imaging experiments.

RESPONSE: All legends describing Ca^{2+} imaging data have been updated stating the concentration of extracellular Ca^{2+}.

Fig. 2: As mentioned above authors may specify, why the specific conditions were analyzed.

RESPONSE: The Legend to Figure 2 (and also Figure EV4) has been updated as follows:

Macropatches were stimulated with 10 µM TPC2-A1-N (b) or 100 nM NAADP (c) under bi-ionic conditions under bi-ionic condition using a pipette (luminal) solution containing 105 mM $CaCl_2$, 5 mM HEPES and 5 mM MES (pH 4.6) and a bath (cytosolic) solution containing 160 mM NaCl and 5 mM HEPES (pH 7.2).

In Fig. 3 c and d, authors utilized Fura-FF, which is a low affinity Ca2+ indicator, used to record high Ca2+ concentrations. Such high concentrations would also be detectable in intact lysosomes or other organelles e.g. the ER. How did the authors exclude a contribution from these organelles in intact cells? This is not clearly described in the methods or results sections.

RESPONSE: We did not exclude any contribution of compartmentalized dye in high Ca^{2+} compartments. We do not think compartmentalization into lysosomes would be a problem because the acidic pH would further lower the affinity of the dye for Ca^{2+} rendering it effectively Ca^{2+}-independent. Compartmentalization into the neutral ER is a potential concern. But in our experience of cell lines, Fura-2 and Fura-FF tend to accumulate predominantly in the cytosol.

Fig. 5: Authors performed the analysis of all mutations in the background of L11A and L12A to re-direct the channel to the plasma membrane. However, the lipid composition of the PM is markedly distinct from the lysosomal membrane and does not contain PI(3,5)P2. Thus, the lipid environment is evidently different from the natural environment of TPC2 and the stimulation with PI(3,5)P2 or synthetic mimicries may be affected. Therefore, authors patched TPC2 in enlarged lysosomes (vacuoles) for the E215A mutant. What were the experimental conditions - which solutions were applied? How did the authors ensure the presence of NAADP accessory proteins (LMS12 or JPT2) in excised plasma membrane patches when applying NAADP? Did authors consider using this method or excised patches from enlarged lysosomes to confirm their obtained results in a more physiologic solution?

RESPONSE: The solutions for the vacuolar patch clamp experiments were the same as those for the macropatches. This was stated in the Methods but has been reiterated in the Legend to Figure 6 as follows:

Enlarged vacuoles were stimulated with 100 nM NAADP (i) or 1 µM PI(3,5)$P_{2(}$ (k) under bi-ionic condition using a pipette (luminal) solution containing 105 mM $CaCl_2$, 5 mM HEPES and 5 mM MES (pH 4.6) and a bath (cytosolic) solution containing 160 mM NaCl and 5 mM HEPES (pH 7.2).

We did not control for the potential loss of NAADP-binding proteins. And are working towards recording under more physiological conditions (see Figure EV5 and response above).

Why were outward current amplitudes extracted at different voltages (+80 mV versus +85 mV) in vacuole patching in Fig. 5 and Fig. S4?

RESPONSE: This is an error. The outward current amplitudes were extracted at +100mV (and the inward currents at -60 mV) as stated in Fig. 5. Fig. S4 (now Appendix Figure S2) has been corrected accordingly.

Gerndt S, Chen CC, Chao YK, Yuan Y, Burgstaller S, Scotto Rosato A, Krogsaeter E, Urban N, Jacob K, Nguyen ONP *et al* (2020) Agonist-mediated switching of ion selectivity in TPC2 differentially promotes lysosomal function. *Elife* 9: e54712

Guo J, Zeng W, Jiang Y (2017) Tuning the ion selectivity of two-pore channels. *Proceedings of the National Academy of Sciences of the United States of America* 114: 1009-1014

Saito R, Mu Q, Yuan Y, Rubio-Alarcón M, Eznarriaga M, Zhao P, Gunaratne G, Kumar S, Keller M, Bracher F *et al* (2023) Convergent activation of Ca(2+) permeability in two-pore channel 2 through distinct molecular routes. *Science signaling* 16: eadg0661

Yuan Y, Jaślan D, Rahman T, Bolsover SR, Arige V, Wagner LE, 2nd, Abrahamian C, Tang R, Keller M, Hartmann J *et al* (2022) Segregated cation flux by TPC2 biases Ca(2+) signaling through lysosomes. *Nature communications* 13: 4481

Dear Sandip,

We have now received re-review reports from both referees, which I have included below. As you will see, you have addressed their concerns satisfactorily. Before I can finally accept the manuscript, there are some remaining editorial points which need to be addressed. In this regard would you please:

- use a heading for the abstract, and remove bold type,
- use a heading for the reference section,
- enter author Afroditi-Maria Zaki in our online submission system in Roman script,
- declare employment in a biotech company in the disclosure and competing interests statement,
- acknowledge the following funding in our online submission system: DFG grants GR4315/2-2, ANR/DFG, GR4315/6-1, SFB/TRR152 P04, SFB1328 A21, GRK2338 P08 and BR 1034/7-1, and NIH grants GM088790, DA 054921 and DA 056729,
- rename the conflict of interest statement as the "Disclosure and competing interests statement",
- remove the author credit section from the text,
- list figure callouts in the text sequentially; callouts for Fig. S1-S3 and Table S1-S2 should be corrected to Appendix Figure S1-S3 and Appendix Table S1-S2,
complete the general info table in the author checklist file and select 'data availability' responses to for the last paragraph,
- upload the appendix file in PDF format; Appendix figures and tables need to be removed from the main manuscript and compiled in the Appendix PDF; its title page should contain "Appendix for Remote control of Ca2+ permeability in a lysosomal ion channel underpins biased agonist activation" and ToC with the page numbers for the listed items; nomenclature correct (Appendix Figure/Table Sx),
- remove the reagents and tools table from the main manuscript file and upload as a separate file using the template from our guide to authors,
- save source data files in a scheme of one figure per folder and then upload as .zip files. E.g. all the Source data files for figure 1 need to be saved in a single folder and this needs to be zipped and then uploaded as "SD figure 1.zip" file. For EV and/or appendix figures, ZIP together all source data,
- state exact p values in the legends of figures 1C, E, H; 2F, H, K; 3D; 4I, J; 5B, D, F, H, L; 6E, G; EV3 B, D; EV4 G, EV5 B, D, F,
- define the scale bar in figures 6B, C,
- rename movies as Movie EV1-EV2 with the corresponding callouts in the main manuscript file; remove their legends from the manuscript text and zip with each movie file, and
- use the following section order: Title page - Abstract - Introduction - Results - Discussion - Methods - Data Availability - Acknowledgements - Disclosure and Competing Interests Statement - References - Figure Legends - Table(s) - Expanded View Figure Legends.

We include a synopsis of the paper (see http://emboj.embopress.org/). Please provide me with a general summary image (measuring 550x300 pixels), a two sentence statement and 3-5 bullet points that capture the key findings of the paper.

I am looking forward to receiving your revised manuscript.

EMBO Press is an editorially independent publishing platform for the development of EMBO scientific publications.

Best wishes,

William

William Teale, PhD
Editor
The EMBO Journal
w.teale@embojournal.org

Instructions for preparing your revised manuscript:

Read our guidance for manuscript revisions and related editorial policies: https://link.springer.com/journal/44318/submission-guidelines#cms-Revised-submissions

Please also check that the title and abstract of the manuscript are brief, yet explicit, even to non-specialists.

When assembling figures, please refer to our figure preparation guideline in order to ensure proper formatting and readability in print as well as on screen:
https://media.springernature.com/original/springer-cms/rest/v1/content/27825798/data/v1

IMPORTANT: When you send the revision we will require
- a point-by-point response to the referees' comments, with a detailed description of the changes made (as a word file).
- a word file of the manuscript text.
- individual production quality figure files (one file per figure)
- a complete author checklist
- Expanded View files (replacing Supplementary Information)
- a Reagents and Tools Table as part of the Methods section

Please remember: Digital image enhancement is acceptable practice, as long as it accurately represents the original data and conforms to community standards. If a figure has been subjected to significant electronic manipulation, this must be noted in the figure legend or in the 'Methods' section. The editors reserve the right to request original versions of figures and the original images that were used to assemble the figure.

We realize that it is difficult to revise to a specific deadline. In the interest of protecting the conceptual advance provided by the work, we recommend a revision within 3 months (6th Aug 2026). Please discuss the revision progress ahead of this time with the editor if you require more time to complete the revisions. Use the link below to submit your revision:

https://emboj.msubmit.net/cgi-bin/main.plex

Referee #1:

The authors have adequately addressed my concerns, and I support the publication of this manuscript in EMBO.

Referee #2:

The authors convincingly answered all questions and included additional experiments to the original draft. I have no further comments or concerns. The manuscript covers a novel topic and is well written; results are clearly presented. In my opinion, it is ready for publication.

Point by point response

- use a heading for the abstract, and remove bold type,

DONE

- use a heading for the reference section,

DONE

- enter author Afroditi-Maria Zaki in our online submission system in Roman script,

UNABLE TO CHANGE DUE TO EMAIL ADDRESS CLASHES

- declare employment in a biotech company in the disclosure and competing interests statement,

N/A

- acknowledge the following funding in our online submission system: DFG grants GR4315/2-2, ANR/DFG, GR4315/6-1, SFB/TRR152 P04, SFB1328 A21, GRK2338 P08 and BR 1034/7-1, and NIH grants GM088790, DA 054921 and DA 056729,

DONE

- rename the conflict of interest statement as the "Disclosure and competing interests statement",

DONE

- remove the author credit section from the text,

DONE

- list figure callouts in the text sequentially; callouts for Fig. S1-S3 and Table S1-S2 should be corrected to Appendix Figure S1-S3 and Appendix Table S1-S2,

DONE

complete the general info table in the author checklist file and select 'data availability' responses to for the last paragraph,

DONE

- upload the appendix file in PDF format; Appendix figures and tables need to be removed from the main manuscript and compiled in the Appendix PDF; its title page should contain "Appendix for Remote control of Ca2+ permeability in a lysosomal ion channel underpins biased agonist activation" and ToC with the page numbers for the listed items; nomenclature correct (Appendix Figure/Table Sx),

DONE

- remove the reagents and tools table from the main manuscript file and upload as a separate file using the template from our guide to authors,

TABLE HAS BEEN UPLOADED SPERATELY BUT LEFT IN MANUSCRIPT FILE AS REMOVAL MESSES UP REFERENCES.

- save source data files in a scheme of one figure per folder and then upload as .zip files. E.g. all the Source data files for figure 1 need to be saved in a single folder and this needs to be zipped and then uploaded as "SD figure 1.zip" file. For EV and/or appendix figures, ZIP together all source data,

DONE

- state exact p values in the legends of figures 1C, E, H; 2F, H, K; 3D; 4I, J; 5B, D, F, H, L; 6E, G; EV3 B, D; EV4 G, EV5 B, D, F,

DONE EXCEPT FOR p VALUES <0.00001 WHICH ARE NOT RETURNED AS EXACT VALUES IN THE STATISTICS PACKAGE WE USE.

- define the scale bar in figures 6B, C,

DONE

- rename movies as Movie EV1-EV2 with the corresponding callouts in the main manuscript file; remove their legends from the manuscript text and zip with each movie file, and

DONE

- use the following section order: Title page - Abstract - Introduction - Results - Discussion - Methods - Data Availability - Acknowledgements - Disclosure and Competing Interests Statement - References - Figure Legends - Table(s) - Expanded View Figure Legends.

DONE

We include a synopsis of the paper (see http://emboj.embopress.org/). Please provide me with a general summary image (measuring 550x300 pixels), a two sentence statement and 3-5 bullet points that capture the key findings of the paper.

GENERAL SUMMARY IMAGE UPLOADED.

TWO SENTENCE SUMMARY:

Lysosomal TPC2 channels are unusual in switching between Ca^{2+}- and Na^{+}-permeable states upon ligand activation. We show that residues remote from the selectivity filter govern this plasticity by controlling agonist discrimination, synergistic channel activation, and lysosomal Ca^{2+} flux, thereby safeguarding organismal fitness.

KEY FINDINGS:

- Ion selectivity of TPC2 can be controlled by residues outside the canonical selectivity filter.
- Gain-of-function mutations reduce discrimination between NAADP- and PI(3,5)P_2-dependent signalling modes.
- Mutant channels exhibit enhanced Ca^{2+} permeability and adopt a co-activated-like state.

- Gain-of-function TPC2 mutants drive excessive lysosomal Ca^{2+} flux that impairs locomotion and viability in vivo.
- Agonist discrimination acts as a safeguarding mechanism to constrain pathological lysosomal Ca^{2+} signalling.

2nd Revision - Editorial Decision 6th Jul 2026

Dear Sandip,

This email is to confirm that your manuscript is now available for you to make minor editorial changes.

Best wishes,

William

William Teale, PhD
Editor
The EMBO Journal
w.teale@embojournal.org

All editorial and formatting issues were resolved by the authors.

Dear Sandip,

I am pleased to inform you that your manuscript has been accepted for publication in the EMBO Journal.

Congratulations to you and all involved!

Your manuscript will be processed for publication by EMBO Press. It will be copy edited and you will receive page proofs prior to publication. Please note that you will be contacted by Springer Nature Author Services to complete licensing and payment information.

You may qualify for financial assistance for your publication charges - either via a Springer Nature fully open access agreement or an EMBO initiative. Check your eligibility: https://link.springer.com/journal/44318/how-to-publish-with-us

Should you be planning a Press Release on your article, please notify the production team via this form in order to coordinate publication and release dates: https://forms.cloud.microsoft/e/eH4A8JVP6g

If you have any questions, please do not hesitate to contact the Editorial Office. Thank you for your contribution to The EMBO Journal.

Best wishes,

Will

William Teale, PhD
Editor
The EMBO Journal
w.teale@embojournal.org

*****
Please note that it is The EMBO Journal policy for the transcript of the editorial process (containing referee reports and your response letters) to be published as an online supplement to each paper. If you should prefer removal of any referee-only figures included in the point-by-point response(s), e.g. because they may still be used for future publication or because they have been reproduced from published work by others, please do let us know immediately via response email.
More information is available here: https://link.springer.com/partners/embo-press/editorial-policies#Peer%20review

** Click here to be directed to your login page: https://emboj.msubmit.net

EMBO Press Author Checklist

Corresponding Author Name: Sandip Patel and Taufiq Rahman	
Journal Submitted to: The EMBO Journal	
Manuscript Number: EMBOJ-2025-122930	

USEFUL LINKS FOR COMPLETING THIS FORM
The EMBO Journal - Author Guidelines
EMBO Reports - Author Guidelines
Molecular Systems Biology - Author Guidelines
EMBO Molecular Medicine - Author Guidelines

Reporting Checklist for Life Science Articles (updated January
This checklist is adapted from Materials Design Analysis Reporting (MDAR) Checklist for Authors. MDAR establishes a minimum set of requirements in transparent reporting in the life sciences (see Statement of Task: 10.31222/osf.io/9sm4x). Please follow the journal's guidelines in preparing your
Please note that a copy of this checklist will be published alongside your article.

Abridged guidelines for figures
1. Data
The data shown in figures should satisfy the following conditions:
- the data were obtained and processed according to the field's best practice and are presented to reflect the results of the experiments in an accurate and unbiased manner.
- ideally, figure panels should include only measurements that are directly comparable to each other and obtained with the same assay.
- plots include clearly labeled error bars for independent experiments and sample sizes. Unless justified, error bars should not be shown for technical
- if n<5, the individual data points from each experiment should be plotted. Any statistical test employed should be justified.
- Source Data should be included to report the data underlying figures according to the guidelines set out in the authorship guidelines on Data

2. Captions
Each figure caption should contain the following information, for each panel where they are relevant:
- a specification of the experimental system investigated (eg cell line, species name).
- the assay(s) and method(s) used to carry out the reported observations and measurements.
- an explicit mention of the biological and chemical entity(ies) that are being measured.
- an explicit mention of the biological and chemical entity(ies) that are altered/varied/perturbed in a controlled manner.
- the exact sample size (n) for each experimental group/condition, given as a number, not a range;
- a description of the sample collection allowing the reader to understand whether the samples represent technical or biological replicates (including how many animals, litters, cultures, etc.).
- a statement of how many times the experiment shown was independently replicated in the laboratory.
- definitions of statistical methods and measures:
  - common tests, such as t-test (please specify whether paired vs. unpaired), simple χ2 tests, Wilcoxon and Mann-Whitney tests, can be unambiguously identified by name only, but more complex techniques should be described in the methods section;
  - are tests one-sided or two-sided?
  - are there adjustments for multiple comparisons?
  - exact statistical test results, e.g., P values = x but not P values < x;
  - definition of 'center values' as median or average;
  - definition of error bars as s.d. or s.e.m.

Please complete ALL of the questions below.
Select "Not Applicable" only when the requested information is not relevant for your study.

Materials

Newly Created Materials	Information included in the manuscript?	In which section is the information available? (Reagents and Tools Table, Materials and Methods, Figures, Data Availability Section)
New materials and reagents need to be available; do any restrictions apply?	Not Applicable	

Antibodies	Information included in the manuscript?	In which section is the information available? (Reagents and Tools Table, Materials and Methods, Figures, Data Availability Section)
For **antibodies** provide the following information:<br>- Commercial antibodies: RRID (if possible) or supplier name, catalogue number and or/clone number<br>- Non-commercial: RRID or citation	Yes	Reagent Table

DNA and RNA sequences	Information included in the manuscript?	In which section is the information available? (Reagents and Tools Table, Materials and Methods, Figures, Data Availability Section)
**Short novel DNA or RNA including primers, probes**: provide the sequences.	Yes	Table S1

Cell materials	Information included in the manuscript?	In which section is the information available? (Reagents and Tools Table, Materials and Methods, Figures, Data Availability Section)
**Cell lines:** Provide species information, strain. Provide accession number in repository **OR** supplier name, catalog number, clone number, and/**OR** RRID.	Yes	Reagent Table
**Primary cultures:** Provide species, strain, sex of origin, genetic modification status.	Not Applicable	
Report if the cell lines were recently **authenticated** (e.g., by STR profiling) and tested for mycoplasma contamination.	Yes	Cell line section in Methods

Experimental animals	Information included in the manuscript?	In which section is the information available? (Reagents and Tools Table, Materials and Methods, Figures, Data Availability Section)
**Laboratory animals or Model organisms:** Provide species, strain, sex, age, genetic modification status. Provide accession number in repository **OR** supplier name, catalog number, clone number, **OR** RRID.	Yes	C. elegans section in Methods
**Animal observed in or captured from the field:** Provide species, sex, and age where possible.	Not Applicable	
Please detail **housing and husbandry conditions**.	Not Applicable	

Plants and microbes	Information included in the manuscript?	In which section is the information available? (Reagents and Tools Table, Materials and Methods, Figures, Data Availability Section)
**Plants:** provide species and strain, ecotype and cultivar where relevant, unique accession number if available, and source (including location for collected wild specimens).	Not Applicable	
**Microbes:** provide species and strain, unique accession number if available, and source.	Not Applicable	

Human research participants	Information included in the manuscript?	In which section is the information available? (Reagents and Tools Table, Materials and Methods, Figures, Data Availability Section)
If collected and within the bounds of privacy constraints report on age, sex and gender or ethnicity for all study participants.	Not Applicable	

Core facilities	Information included in the manuscript?	In which section is the information available? (Reagents and Tools Table, Materials and Methods, Figures, Data Availability Section)
If your work benefited from core facilities, was their service mentioned in the acknowledgments section?	Not Applicable	

Design

Study protocol	Information included in the manuscript?	In which section is the information available? (Reagents and Tools Table, Materials and Methods, Figures, Data Availability Section)

If study protocol has been **pre-registered, provide DOI in the manuscript**. For clinical trials, provide the trial registration number **OR** cite DOI.	Not Applicable	
Report the **clinical trial registration number** (at ClinicalTrials.gov or equivalent), where applicable.	Not Applicable	

Laboratory protocol	Information included in the manuscript?	In which section is the information available? (Reagents and Tools Table, Materials and Methods, Figures, Data Availability Section)
Provide DOI OR other citation details if **external detailed step-by-step protocols** are available.	Not Applicable	

Experimental study design and statistics	Information included in the manuscript?	In which section is the information available? (Reagents and Tools Table, Materials and Methods, Figures, Data Availability Section)
Include a statement about **sample size** estimate even if no statistical methods were used.	Yes	Statistics section in Methods
Were any steps taken to minimize the effects of subjective bias when allocating animals/samples to treatment (e.g. **randomization procedure**)? If yes, have they been described?	Not Applicable	
Include a statement about **blinding** even if no blinding was done.	Yes	Statistics section in Methods
Describe **inclusion/exclusion criteria** if samples or animals were excluded from the analysis. Were the criteria pre-established?<br><br>If sample or data points were omitted from analysis, report if this was due to attrition or intentional exclusion and provide justification.	Not Applicable	
For every figure, are **statistical tests** justified as appropriate? Do the data meet the assumptions of the tests (e.g., normal distribution)? Describe any methods used to assess it. Is there an estimate of variation within each group of data? Is the variance similar between the groups that are being statistically compared?	Yes	Figure Legends

Sample definition and in-laboratory replication	Information included in the manuscript?	In which section is the information available? (Reagents and Tools Table, Materials and Methods, Figures, Data Availability Section)
In the figure legends: state number of times the experiment was **replicated** in laboratory.	Yes	
In the figure legends: define whether data describe **technical or biological replicates**.	Yes	

Ethics

Ethics	Information included in the manuscript?	In which section is the information available? (Reagents and Tools Table, Materials and Methods, Figures, Data Availability Section)
Studies involving **human participants**: State details of **authority granting ethics approval** (IRB or equivalent committee(s), provide reference number for approval.	Not Applicable	
Studies involving **human participants**: Include a statement confirming that **informed consent** was obtained from all subjects and that the experiments conformed to the principles set out in the WMA Declaration of Helsinki and the Department of Health and Human Services Belmont Report.	Not Applicable	
Studies involving **human participants:** For publication of **patient photos**, include a statement confirming that consent to publish was obtained.	Not Applicable	
Studies involving experimental **animals**: State details of **authority granting ethics approval** (IRB or equivalent committee(s), provide reference number for approval. Include a statement of compliance with ethical regulations.	Not Applicable	
Studies involving **specimen and field samples:** State if relevant **permits** obtained, provide details of authority approving study; if none were required, explain why.	Not Applicable	

Dual Use Research of Concern (DURC)	Information included in the manuscript?	In which section is the information available? (Reagents and Tools Table, Materials and Methods, Figures, Data Availability Section)
Could your study fall under dual use research restrictions? Please check biosecurity documents and list of **select agents and toxins** (CDC): https://www.selectagents.gov/sat/list.htm	Not Applicable	
If you used a select agent, is the security level of the lab appropriate and reported in the manuscript?	Not Applicable	
If a study is subject to dual use research of concern regulations, is the name of the **authority granting approval and reference number** for the regulatory approval provided in the manuscript?	Not Applicable	

Reporting

The MDAR framework recommends adoption of discipline-specific guidelines, established and endorsed through community initiatives. Journals have their own policy about requiring specific guidelines and recommendations to complement MDAR.

Adherence to community standards	Information included in the manuscript?	In which section is the information available? (Reagents and Tools Table, Materials and Methods, Figures, Data Availability Section)
State if relevant guidelines or checklists (e.g., **ICMJE, MIBBI, ARRIVE, PRISMA**) have been followed or provided.	Not Applicable	
For **tumor marker prognostic studies,** we recommend that you follow the **REMARK** reporting guidelines (see link list at top right). See author guidelines, under 'Reporting Guidelines'. Please confirm you have followed these guidelines.	Not Applicable	
For **phase II and III randomized controlled trials**, please refer to the **CONSORT** flow diagram (see link list at top right) and submit the CONSORT checklist (see link list at top right) with your submission. See author guidelines, under 'Reporting Guidelines'. Please confirm you have submitted this list.	Not Applicable	

Data Availability

Data availability	Information included in the manuscript?	In which section is the information available? (Reagents and Tools Table, Materials and Methods, Figures, Data Availability Section)
Have **primary datasets** been deposited according to the journal's guidelines (see 'Data Deposition' section) and the respective accession numbers provided in the Data Availability Section?	Not Applicable	
Were **human clinical and genomic datasets** deposited in a public access-controlled repository in accordance to ethical obligations to the patients and to the applicable consent agreement?	Not Applicable	
Are **computational models** that are central and integral to a study available without restrictions in a machine-readable form? Were the relevant accession numbers or links provided?	Not Applicable	
If publicly available data were reused, provide the respective **data citations in the reference list**.	Not Applicable	
