# Supplementary material for: Allosteric switching of Ca2+ permeability in the lysosomal ion channel TPC2 underpins biased agonist activation
Source: EMBO J. 2026 Aug 7;45(18):6501–26. doi: 10.1038/s44318-026-00882-1 (PMC13578460; doi:10.1038/s44318-026-00882-1)
Supplement: Supplementary file 5 [file 44318_2026_882_MOESM3_ESM.zip › SD Movie EV1/Movie EV1 Legend.docx]

**Movie EV1. Interaction between the S4-S5 linker and the channel gate in TPC2.**
